# Supplementary material for: A cluster randomised controlled trial of community groups using Participatory Learning and Action to prevent and control diabetes and intermediate hyperglycaemia in rural Bangladesh
Source: PLOS Glob Public Health. 2025 Aug 14;5(8):e0005049. doi: 10.1371/journal.pgph.0005049 (PMC12352636; doi:10.1371/journal.pgph.0005049)
Supplement: S1 Table — (DOCX) [file pgph.0005049.s001.docx]

**S1 Table: Participatory Learning and Action (PLA) group design and adaptations in response to COVID-19**

| **Design parameter** | **Original design** | **Adaptation** |
| --- | --- | --- |
| Group composition | Open to all community members, with no restrictions on numbers in meetings. DMagic’s average group size was 32. | Open to all community members, but meeting size restricted to maximum of 20 participants or less per meeting. |
| Meeting frequency | Once a month per village | Twice a month per village, to allow for smaller groups but with the same population coverage |
| Meeting conduct | Participants sit in a circle, and active participation is encouraged. | Participants were asked to wash their hands on arrival/departure, to wear a mask, and try to keep a minimum of 1-meter distance from group members outside of their households. All materials used during the meeting were wiped with alcohol between meetings. |
| Facilitator role | Facilitators hold 9 meetings per month | Facilitators held 18 meetings per month. Facilitators travelled using well-ventilated transport (e.g. rickshaw) and adhered to infection control practices. |
| Group communication | Meetings arranged in-person | Phone numbers of group members were collected (after consent) to facilitate communication around meetings, and if needed, to cancel meetings. Numbers were stored locally by facilitators. |
| Total number of meetings | 18 meetings per group delivered over 18 months | Minimum of 13 meetings per group delivered over 20 months. This required more topics to be covered in each meeting. |
| Community meetings | Large community meetings with several hundred participants held | Smaller stakeholder meetings and use of alternative methods to disseminate group information to wider community, e.g. loudspeaker announcements. |
