# Supplementary material for: A cluster randomised controlled trial of community groups using Participatory Learning and Action to prevent and control diabetes and intermediate hyperglycaemia in rural Bangladesh
Source: PLOS Glob Public Health. 2025 Aug 14;5(8):e0005049. doi: 10.1371/journal.pgph.0005049 (PMC12352636; doi:10.1371/journal.pgph.0005049)
Supplement: S2 Table — (DOCX) [file pgph.0005049.s002.docx]

**S2 Table: Glycaemic definitions and diagnostic criteria used in the D:Clare trial, adapted from WHO 2006**

| **Definition** | | **Diagnostic Criteria** |
| --- | --- | --- |
| *Normoglycaemia* | | Fasting plasma glucose <6.0 mmol/L |
| *Intermediate Hyperglycaemia* | Impaired Fasting Glucose (IFG) | Fasting plasma glucose >6.1 mmol/L to <7.0 mmol/L **AND**  two-hour post ingestion of 75g glucose load plasma glucose <7.8 mmol/L |
|  | Impaired Glucose Tolerance (IGT) | Fasting plasma glucose <7.0 mmol/L  **AND**  two-hour post ingestion of 75g glucose load plasma glucose >7.8 mmol/L to <11.1 mmol/L |
| *Type 2 Diabetes Mellitus (T2DM)* | | Fasting plasma glucose >7.0 mmol/L  **OR***  two-hour post ingestion of 75g glucose load plasma glucose >11.1 mmol/L  **OR**  Self-reported prior medical diagnosis of diabetes |

**Diabetes cannot be excluded without 2-h post oral glucose load test*

*Source:* [*http://apps.who.int/iris/bitstream/10665/43588/1/9241594934_eng.pdf*](http://apps.who.int/iris/bitstream/10665/43588/1/9241594934_eng.pdf)
