# Supplementary material for: A cluster randomised controlled trial of community groups using Participatory Learning and Action to prevent and control diabetes and intermediate hyperglycaemia in rural Bangladesh
Source: PLOS Glob Public Health. 2025 Aug 14;5(8):e0005049. doi: 10.1371/journal.pgph.0005049 (PMC12352636; doi:10.1371/journal.pgph.0005049)
Supplement: S3 Table — (DOCX) [file pgph.0005049.s003.docx]

**S3 Table: D:Clare trial secondary outcome measures and definitions**

| Outcome | Definition | Denominator | Data type |
| --- | --- | --- | --- |
| awareness of diabetic status | self-reported diabetes | endline cross-sectional sample with valid endline blood glucose measurements that meet the classification of diabetes and who complete the endline questionnaire | Binary |
| physical activity | average time spent engaged in physical activity per week | endline cross-sectional sample with valid self-reported physical activity data | Continuous |
| blood pressure | diastolic and systolic blood pressure | endline cross-sectional sample with valid blood pressure measurements | Continuous |
| Hypertension | systolic blood pressure ≥ 140 mmHg or a diastolic blood pres­sure ≥ 90 mmHg or current treatment with antihypertensive medication | endline cross-sectional sample with valid blood pressure measurements | Binary |
| Body Mass Index | body mass index (weight (kg)/height(m)^2^ | endline cross-sectional sample with valid weight and height measurements | Continuous |
| abdominal obesity | waist:hip ratio (WHR) | endline cross-sectional sample with valid waist and hip measurements | Continuous |
| dietary diversity | Dietary Diversity Score (DDS) | endline cross-sectional sample with valid DDS data | Continuous |
| knowledge about diabetes symptoms and complications (five separate outcomes) | proportion of adults aged 30 years and above who are able to: a) name at least one cause of diabetes; b) report at least one symptom of diabetes; c) report at least one complication of diabetes; d) report at least one way to reduce the risk of getting diabetes; e) report at least one way to control diabetes if diagnosed. | endline cross-sectional sample of with valid knowledge data | Binary |
| utilisation of services for treatment or advice for diabetes | proportion of diabetics with self-reported diagnosis of diabetes reporting current or ever receipt of care or advice from a medical professional | endline cross-sectional sample of known (pre-diagnosed) diabetic individuals | Binary |
| depression | proportion of adults aged 30 years and above with a PHQ score ~~>~~10 (i.e. moderate to severe depressive symptoms). | endline cross-sectional sample of with valid PHQ data. | Binary |
| anxiety | proportion of adults aged 30 years and above with a GAD-7 score >10. | endline cross-sectional sample of with valid GAD-7 data | Binary |
| 18 month cumulative incidence of T2DM among individuals with intermediate hyperglycaemia at baseline | proportion of adults aged 30 years or older with plasma glucose cut-off categorisations for intermediate hyperglycaemia at baseline who are categorised as T2DM at endline | cohort sample of adults aged 30 years or older with plasma glucose cut-off intermediate hyperglycaemia at baseline and followed-up to endline | Binary |
