# Supplementary material for: A cluster randomised controlled trial of community groups using Participatory Learning and Action to prevent and control diabetes and intermediate hyperglycaemia in rural Bangladesh
Source: PLOS Glob Public Health. 2025 Aug 14;5(8):e0005049. doi: 10.1371/journal.pgph.0005049 (PMC12352636; doi:10.1371/journal.pgph.0005049)
Supplement: S4 Table — (DOCX) [file pgph.0005049.s004.docx]

### S4 Table: Sensitivity analysis of the primary outcome showing frequencies, proportions, estimated difference (intervention minus control) and 95% confidence interval comparing prevalence of intermediate hyperglycaemia and diabetes between trial arms using different outcome definitions.

| **OUTCOME** | **Baseline** | | **Endline** | | **Crude Difference (95% CI)*** | **Adjusted Difference (95% CI)**** |
| --- | --- | --- | --- | --- | --- | --- |
|  | **Control** | **Intervention** | **Control** | **Intervention** |  |  |
| **Sensitivity analysis 1: All cases of self-reported diabetes excluded** | 237 (38.8%) | 222 (33.7%) | 260 (44.0%) | 260 (41.1%) | 0.01 (-10.27, 10.40); p=0.9887 | 0.18 (-9.86, 10.22); p=0.9682 |
| **Sensitivity analysis 2: All cases defined based on blood glucose measures only (disregarding self-reported diabetes)** | 266 (39.1%) | 246 (34.8%) | 290 (43.9%) | 281 (41.1%) | -0.69 (-11.03, 9.67); p=0.8837 | -0.37 (-10.56, 9.80); p=0.9349 |
| **Sensitivity analysis 3a^: Arbitrary cut-off definitions of intermediate hyperglycemia and diabetes.** | 420 (68.7%) | 401 (60.9%) | 433 (73.3%) | 471 (74.5%) | 5.47 (-7.75, 18.69); p=0.3735 | 5.35 (-7.56, 18.26); p=0.3732 |
| **Sensitivity analysis 3b^^: Arbitrary cut-off definitions of intermediate hyperglycemia and diabetes.** | 369 (60.4%) | 349 (53.0%) | 393 (66.5%) | 385 (60.9%) | -1.42 (-14.75, 11.91); p=0.8152 | -1.60 (-14.54, 11.34); p=0.7863 |

* Adjustment for baseline outcome measure at the cluster level.
**Adjusted for cluster-level baseline outcome measure, gender, and age as linear and quadratic terms.
^ IFG fasting glucose 5.5-6.2 mmol/L & 2h glucose <10.4 mmol/L; IGT fasting glucose<6.3 mmol/L & 2h glucose 6.8-10.3 mmol/L; diabetes fasting glucose>6.3 mmol/L or 2h glucose >10.4 mmol/L.
^^ IFG fasting glucose 6.3-7.7 mmol/L & 2h glucose <10.4 mmol/L; IGT fasting glucose<7.8 mmol/L & 2h glucose 6.8-10.3 mmol/L; diabetes fasting glucose>7.8 mmol/L or 2h glucose >10.4 mmol/L.
