# Supplementary material for: A cluster randomised controlled trial of community groups using Participatory Learning and Action to prevent and control diabetes and intermediate hyperglycaemia in rural Bangladesh
Source: PLOS Glob Public Health. 2025 Aug 14;5(8):e0005049. doi: 10.1371/journal.pgph.0005049 (PMC12352636; doi:10.1371/journal.pgph.0005049)
Supplement: S5 Table — (DOCX) [file pgph.0005049.s005.docx]

**S5 Table: Post-hoc analysis of intervention effects on pulse pressure, mean arterial pressure and log-transformed anxiety (GAD-7) score.**

|  | **Baseline** | | **Endline** | | **Crude Difference (95%CI)*** | **Adjusted Difference (95%CI)**** |
| --- | --- | --- | --- | --- | --- | --- |
|  | **Control** | **Intervention** | **Control** | **Intervention** |  |  |
| **Pulse pressure mmHg, mean (SD)** | 57.9 (17.7) | 54.0 (16.2) | 51.1 (15.0) | 49.2 (13.4) | -1.97 (-3.83, -0.12); p=0.0394 | -1.97 (-3.77, -0.16); p=0.0358 |
| **Mean arterial pressure, mmHg (SD)** | 95.0 (13.7) | 97.2 (13.8) | 92.9 (13.2) | 94.0 (12.6) | -0.83 (-3.21, 1.54); p=0.4473 | -1.27 (-3.57, 1.02); p=0.2403 |
| **Log GAD7 score (median, IQR)** | 1.36 (1.10-1.95) | 1.28 (0.69-1.95) | 1.17 (0.69-1.61) | 0.93 (0.69-1.39) | -0.24 (-0.43, -0.05); p=0.0181 | -0.22 (-0.41, -0.04); p=0.0201 |

* Adjustment for baseline outcome measure at the cluster level.
**Adjusted for cluster-level baseline outcome measure, gender, and age as linear and quadratic terms.
