# Supplementary material for: A cluster randomised controlled trial of community groups using Participatory Learning and Action to prevent and control diabetes and intermediate hyperglycaemia in rural Bangladesh
Source: PLOS Glob Public Health. 2025 Aug 14;5(8):e0005049. doi: 10.1371/journal.pgph.0005049 (PMC12352636; doi:10.1371/journal.pgph.0005049)
Supplement: S6 Table — Odds ratios (for binary outcomes) and coefficients (for continuous outcomes) indicate the difference in intervention effect on outcomes between interaction categories. (DOCX) [file pgph.0005049.s006.docx]

**S6 Table:** **Interaction odds ratios and regression coefficients (95% CIs) between trial arm and gender, wealth index, age, village size and inclusion in baseline sample for primary and secondary study outcomes. Odds ratios (for binary outcomes) and coefficients (for continuous outcomes) indicate the difference in intervention effect on outcomes between interaction categories.**

| **Outcomes** | | **Gender**  **(male compared to female)** | **Wealth Index**  **(per unit increase in wealth index)** | **Age**  **(per year increase in age)** | **Village size**  **(bigger than average compared to smaller than average village)** | **In baseline survey (yes compared to no)** |
| --- | --- | --- | --- | --- | --- | --- |
| **Primary outcome – prevalence of DM & intermediate hyperglycaemia^+^** | | 1.13 (0.63, 2.01) | 0.98 (0.83, 1.16) | 1.00 (0.98, 1.02) | 1.06 (0.54, 2.09) | 2.12 (0.64, 7.03) |
| **Blood pressure** | **Systolic blood pressure**^ | 0.68 (-6.99, 8.35) | 0.18 (-0.93, 1.28) | -0.04 (-0.21, 0.14) | -0.09 (-6.78, 6.60) | 0.11 (-5.90, 6.11) |
|  | **Diastolic blood pressure**^ | 0.15 (-2.78, 3.08) | -0.21 (-1.19, 0.77) | 0.06 (-0.04, 0.16) | 1.61 (-3.26, 6.48) | 1.41 (-2.76, 5.57) |
|  | **Hypertension^+^** | 1.19 (0.56, 2.53) | 1.12 (0.93, 1.33) | 1.00 (0.98, 1.02) | 0.86 (0.48, 1.54) | 1.22 (0.61, 2.43) |
| **Overweight & obesity** | **Body Mass Index (BMI)**^ | -0.63 (-1.65, 0.38) | -0.18 (-0.46, 0.09) | 0.01 (-0.02, 0.05) | 0.43 (-1.59, 2.45) | -0.94 (-3.17, 1.29) |
|  | **Overweight or obese^+^** | 0.85 (0.54, 1.35) | 0.90 (0.74, 1.10) | 1.01 (0.99, 1.02) | 1.31 (0.59, 2.91) | 0.59 (0.19, 1.89) |
|  | **Waist:Hip ratio**^ | 0.01 (-0.03, 0.04) | 0.00 (0.00, 0.01) | 0.00 (0.00, 0.00) | 0.00 (-0.05, 0.05) | 0.00 (-0.03, 0.03) |
|  | **Abdominal obesity^+^** | 1.24 (0.40, 3.81) | 0.98 (0.84, 1.13) | 1.01 (0.98, 1.03) | 0.63 (0.15, 2.69) | 0.91 (0.27, 3.04) |
| **Dietary diversity score**^ | | 0.42 (-0.04, 0.88) | -0.02 (-0.14, 0.11) | -0.01 (-0.02, 0.01) | -0.57 (-1.32, 0.18) | -0.20 (-0.64, 0.24) |
| **Log minutes spent engaged in physical activity per week**^ | | -0.20 (-0.75, 0.34) | 0.04 (-0.05, 0.13) | -0.00 (-0.01, 0.01) | -0.35 (-0.71, 0.02) | 0.10 (-0.25, 0.45) |
| **Diabetes knowledge** | **Ability to report one or more valid *causes* of diabetes^+^** | 0.66 (0.31, 1.40) | 0.90 (0.69, 1.17) | 0.98 (0.96, 1.01) | 1.42 (0.32, 6.23) | 0.67 (0.23, 1.92) |
|  | **Ability to report one or more valid *symptoms* of diabetes^+^** | 1.01 (0.50, 2.04) | 0.93 (0.75, 1.16) | 0.98 (0.96, 1.00) | 2.14 (0.31, 14.74) | 0.62 (0.22, 1.76) |
|  | **Ability to report one or more valid *complications* of diabetes^+^** | 0.75 (0.34, 1.65) | 0.87 (0.64, 1.17) | 0.98 (0.96, 1.01) | 2.97 (0.43, 20.40) | 0.89 (0.34, 2.35) |
|  | **Ability to report one or more valid ways to *prevent* diabetes^+^** | 0.84 (0.35, 2.05) | 0.96 (0.76, 1.22) | 0.98 (0.96, 1.00) | 2.71 (0.61, 12.03) | 0.69 (0.18, 2.60) |
|  | **Ability to report one or more valid ways to *control* diabetes^+^** | 0.95 (0.38, 2.39) | 0.89 (0.67, 1.19) | 0.99 (0.96, 1.01) | 3.28 (0.72, 15.06) | 0.80 (0.18, 3.61) |
| **PHQ-9 score >10 (moderate or severe depression)^+^** | | 2.24 (0.57, 8.84) | 1.83 (1.26, 2.66)** | 1.00 (0.93, 1.07) | 0.27 (0.06, 1.26) | 2.35 (0.29, 19.00) |
| **GAD-7 score >10 (moderate or severe anxiety)^+^** | | 1.09 (0.16, 7.37) | 1.85 (1.28, 2.68)** | 1.00 (0.93, 1.07) | 0.50 (0.07, 3.40) | 4.65 (0.39, 55.30) |
| **Self-awareness of status among individuals identified with DM by blood glucose measures^+^** | | 0.36 (0.08, 1.60) | 1.32 (0.98, 1.77) | 1.02 (0.98, 1.05) | 1.34 (0.32, 5.70) | 1.24 (0.30, 5.06) |
| **Use of services for treatment/advice for diabetes among individuals with prior diagnosis^+^** | | No data | 0.22 (0.10, 0.46)*** | 1.19 (1.11, 1.28)*** | No data | No data |
| **Two-year diabetes incidence among individuals with intermediate hyperglycaemia^+^** | | 0.57 (0.07, 4.92) | 1.77 (1.12, 2.80)* | 1.03 (0.95, 1.11) | 0.78 (0.12, 5.00) | NA |
| **Self-rated health**^ | | -3.52 (-8.45, 1.41) | -2.19 (-3.93, -0.47)* | 0.12 (-0.05, 0.29) | -1.44 (-8.12, 5.24) | -3.21 (-9.97, 3.56) |
| **Daily smoking or use of smokeless tobacco^+^** | | -0.94 (-6.07, 4.19) | -1.10 (-2.69, 0.49) | -0.05 (-0.17, 0.06) | -2.40 (-5.67, 0.87) | 0.71 (-3.12, 4.54) |
| **Betel nut use^+^** | | 1.31 (0.67, 2.57) | 1.07 (0.93, 1.23) | 1.00 (0.98, 1.02) | 1.51 (0.80, 2.88) | 0.82 (0.32, 2.12) |
| **Log sedentary time in previous 24 hours**^ | | 0.32 (0.17, 0.46)*** | -0.03 (-0.08, 0.02) | -0.00 (-0.01, 0.00) | -0.29 (-0.60, 0.02) | 0.24 (-0.03, 0.50) |
| **Log time engaged in brisk walking**^ | | 0.20 (-0.26, 0.66) | -0.01 (-0.10, 0.07) | 0.00 (-0.01, 0.01) | -0.06 (-0.37, 0.25) | -0.27 (-0.51, -0.02)* |
| **Among people living with diabetes** | | | | | | |
| **Diabetes control^+^** | | 1.21 (0.12, 12.58) | 2.18 (1.17, 4.07)* | 1.00 (0.91, 1.09) | 0.87 (0.18, 4.28) | 0.71 (0.01, 52.92) |
| **Diabetes affect (ADS) score** | | -0.94 (-6.07, 4.19) | -1.10 (-2.69, 0.49) | -0.05 (-0.17, 0.06) | -2.40 (-5.67, 0.87) | 0.71 (-3.12, 4.54) |
| **Diabetes-related complications^+^** | | No data | 0.36 (0.20, 0.65)** | 0.97 (0.89, 1.06) | 1.73 (0.07, 43.93) | 5.74 (0.19, 169.45) |
| **At least monthly blood glucose testing^+^** | | 0.88 (0.09, 8.22) | 0.66 (0.30, 1.44) | 0.96 (0.88, 1.04) | 0.68 (0.02, 21.29) | No data |
| **Stigma 1: Perceived inability to fulfil responsibilities^+^** | | 3.57 (0.64, 19.86) | 0.91 (0.45, 1.81) | 0.96 (0.90, 1.01) | 1.73 (0.03, 110.44) | 0.11 (0.01, 1.17) |
| **Stigma 2: Perceived to be seen as a lesser person^+^** | | 1.84 (0.22, 15.51) | 0.37 (0.22, 0.62)** | 0.96 (0.88, 1.04) | 57.10 (3.18, 1023.88)* | 0.06 (0.00, 1.11) |
| **Stigma 3: Embarrassed in social situations^+^** | | 3.53 (0.49, 25.53) | 0.52 (0.20, 1.35) | 0.93 (0.90, 0.97)** | 20.01 (1.67, 239.86)* | No data |
| **Stigma 4: Ashamed of having diabetes^+^** | | No data | 1.22 (0.76, 1.94) | 1.01 (0.98, 1.05) | 7.78 (0.54, 112.61) | No data |
| **Feels supported by family in managing diabetes^+^** | | 1.96 (0.06, 59.42) | 0.83 (0.50, 1.38) | 0.96 (0.88, 1.05) | 0.38 (0.05, 2.98) | No data |
| **Ever experienced physical, social, or mental abuse due to diabetic status^+^** | | 0.37 (0.04, 3.93) | 0.83 (0.61, 1.11) | 0.94 (0.88, 1.00)* | 0.85 (0.11, 6.69) | No data |
| **Social norms** | | | | | | |
| **1) Going on a morning walk alone (women only)^+^** | | NA | 1.03 (0.64, 1.65) | 0.95 (0.91, 0.98)** | 3.17 (0.17, 59.87) | 1.62 (0.34, 7.76) |
| **2) Going for a morning walk with a female relative (women only)^+^** | | NA | 0.94 (0.62, 1.42) | 0.95 (0.90, 1.01) | 2.16 (0.08, 59.38) | No data |
| **3) Eating less than usual or refusing oily or sugary foods and drinks at social gatherings^+^** | | 1.02 (0.31, 3.33) | 0.98 (0.76, 1.28) | 1.00 (0.97, 1.04) | 0.80 (0.15, 4.40) | 1.16 (0.58, 2.30) |
| **4) Providing healthy snacks to guests^+^** | | 2.73 (1.35, 5.52)** | 0.93 (0.75, 1.15) | 0.99 (0.97, 1.01) | 0.95 (0.10, 9.48) | 1.48 (0.64, 3.43) |

* p<0.05; **p<0.01; ***p<0.001
^Continuous outcome – results show regression coefficient and 95% confidence intervals of interaction term (intervention effect on outcome by column interaction parameter)
+Binary outcome – results show odds ratio and 95% confidence intervals of interaction term (intervention effect on outcome by column interaction parameter)
