# Supplementary material for: A cluster randomised controlled trial of community groups using Participatory Learning and Action to prevent and control diabetes and intermediate hyperglycaemia in rural Bangladesh
Source: PLOS Glob Public Health. 2025 Aug 14;5(8):e0005049. doi: 10.1371/journal.pgph.0005049 (PMC12352636; doi:10.1371/journal.pgph.0005049)
Supplement: S7 Table — (DOCX) [file pgph.0005049.s007.docx]

**S7 Table: Intervention costs disaggregated by input, activities and implementation phase**

| **Description** | **US$** | **%** |
| --- | --- | --- |
| **Inputs/Line items** |  |  |
| Staff | 215,349 | 68% |
| Materials for running PLA | 2,326 | 1% |
| Other materials | - | 0% |
| Materials-COVID protocol | 35,671 | 11% |
| Other recurrent | 41,372 | 13% |
| Travel/Transportation | 23,062 | 7% |
| Capital | 925 | 0% |
| **Activities** |  |  |
| Adapting PLA materials | 23,160 | 2% |
| Community sensitisation | 39,865 | 4% |
| PLA-training | 18,034 | 2% |
| Covid-19 protocols | 95,862 | 10% |
| Other start up activities | 813 | 0% |
| PLA implementation | 448,688 | 48% |
| Coordination | 13,569 | 1% |
| supervision | 6,756 | 1% |
| General M&E | 2,961 | 0% |
| Admin & joint activities | 282,434 | 30% |
| **Implementation phase** |  |  |
| Start up | 55,880 | 18% |
| Implementation | 262,826 | 82% |
| **Total costs** | **318,706** | **100%** |
