# Supplementary material for: A cluster randomised controlled trial of community groups using Participatory Learning and Action to prevent and control diabetes and intermediate hyperglycaemia in rural Bangladesh
Source: PLOS Glob Public Health. 2025 Aug 14;5(8):e0005049. doi: 10.1371/journal.pgph.0005049 (PMC12352636; doi:10.1371/journal.pgph.0005049)
Supplement: S1 Text — (PDF) [file pgph.0005049.s010.pdf]

King et al. Trials (2021) 22:235
https://doi.org/10.1186/s13063-021-05167-y

Trials

STUDY PROTOCOL Open Access

Community participatory learning and action cycle groups to reduce type 2 diabetes in Bangladesh (D:Clare trial): study protocol for a stepped-wedge cluster randomised controlled trial

Carina King[1,2*], Malini Pires[1], Naveed Ahmed[3], Kohenour Akter[3], Abdul Kuddus[3], Andrew Copas[1], Hassan Haghparast-Bidgoli[1], Joanna Morrison[1], Tasmin Nahar[3], Sanjit Kumer Shaha[3], A. K. Azad Khan[3], Kishwar Azad[3] and Edward Fottrell[1]

Abstract

Background: An estimated 463 million people globally have diabetes, with the prevalence growing in low-and middle-income settings, such as Bangladesh. Given the need for context-appropriate interventions to prevent type 2 diabetes mellitus (T2DM), the 'Diabetes: Community-led Awareness, Response and Evaluation' (D:Clare) trial will rigorously evaluate the replication and scale-up of a participatory learning and action (PLA) cycle intervention in Bangladesh, to inform policy on population-level T2DM prevention and control.

Methods: This is a stepped-wedge cluster randomised controlled trial, with integrated process and economic evaluations, conducted from March 2020 to September 2022. The trial will evaluate a community-based four-phase PLA cycle intervention focused on prevention and control of T2DM implemented over 18 months, against a control of usual care. Twelve clusters will be randomly allocated (1:1) to implement the intervention at project month 1 or 12. The intervention will be evaluated through three cross-sectional surveys at months 1, 12 and 24. The trial will be conducted in Alfadanga Upazila, Faridpur district, with an estimated population of 120,000. Clusters are defined as administrative geographical areas, with approximately equal populations. Each of the six unions in Alfadanga will be divided into two clusters, forming 12 clusters in total. Given the risk of inter-cluster contamination, evaluation surveys will exclude villages in border areas. Participants will be randomly sampled, independently for each survey, from a population census conducted in January 2020. The primary outcome is the combined prevalence of intermediate hyperglycaemia and T2DM, measured through fasting and 2-h post-glucose load blood tests. A total of 4680 participants provide 84% power to detect a 30% reduction in the primary outcome, assuming a baseline of 30% and an ICC of 0.07. The analysis will be by intention-to-treat, comparing intervention and control periods across all clusters, adjusting for geographical clustering.

(Continued on next page)

* Correspondence: carina.king@ki.se
[1]Institute for Global Health, University College London, 30 Guildford Street, London WC1N 1EH, UK
[2]Department of Global Public Health, Karolinska Institutet, Stockholm, Sweden
Full list of author information is available at the end of the article

© The Author(s). 2021 **Open Access** This article is licensed under a Creative Commons Attribution 4.0 International License, which permits use, sharing, adaptation, distribution and reproduction in any medium or format, as long as you give appropriate credit to the original author(s) and the source, provide a link to the Creative Commons licence, and indicate if changes were made. The images or other third party material in this article are included in the article's Creative Commons licence, unless indicated otherwise in a credit line to the material. If material is not included in the article's Creative Commons licence and your intended use is not permitted by statutory regulation or exceeds the permitted use, you will need to obtain permission directly from the copyright holder. To view a copy of this licence, visit http://creativecommons.org/licenses/by/4.0/. The Creative Commons Public Domain Dedication waiver (http://creativecommons.org/publicdomain/zero/1.0/) applies to the data made available in this article, unless otherwise stated in a credit line to the data.

(Continued from previous page)
Discussion: This study will provide further evidence of effectiveness for community-based PLA to prevent T2DM at scale in a rural Bangladesh setting. However, we encountered several challenges in applying the stepped-wedge design to our research context, with particular consideration given to balancing seasonality, timing and number of steps and estimation of partial versus full effect.

Trial registration: ISRCTN: ISRCTN42219712. Registered on 31 October 2019

Keywords: Diabetes, T2DM, Non-communicable diseases, Bangladesh, Participatory learning and action, Stepped-wedge trial

Background

An estimated 463 million people globally have diabetes, with prevalence rising [1]. Currently, 75% of type 2 diabetes mellitus (T2DM) cases are thought to occur in low- and middle-income countries (LMICs) [2]. In South Asia, the increased incidence of diabetes has been attributed to rapid increases in income and urbanisation [3]. However, the prevalence of both T2DM and intermediate hyperglycaemia—including both impaired fasting glucose and impaired glucose tolerance—is also growing rapidly in rural areas [4]. This trend is seen in Bangladesh, with a rural community-based survey in 2016 finding more than one third of adults over 30 years of age had raised blood glucose levels [4].

T2DM is associated with a combination of behavioural risk factors, including sedentary behaviour and low physical activity, poor diet and smoking, alongside metabolic risk factors of hypertension, overweight and obesity, and genetic predisposition and gene-environment interactions [5]. Prevention and control efforts have predominantly targeted individual behaviour change amongst high-risk groups, with mixed results in South Asian settings [6–11].

There is a need to develop and test population-level interventions that create an enabling environment for the prevention of T2DM at scale, shifting from individualistic to structural and social interventions [12]. The DMagic (Diabetes Mellitus Action through community Groups or mHealth Information for better Control) trial used a participatory learning and action (PLA) approach directed at the general population in rural Bangladesh [13]. In this trial, significant increases in T2DM knowledge and awareness and reduced prevalence of T2DM and intermediate hyperglycaemia were reported. Amongst individuals identified with intermediate hyperglycaemia, the 2-year cumulative incidence odds of T2DM was 59% lower, equating to an absolute reduction of 21% for T2DM and 9% for intermediate hyperglycaemia. However, no significant changes were observed for diet, exercise and smoking behaviours. The intervention was highly cost-effective. Assuming a 30% loss of effectiveness at scale, an estimated 240,000 individuals could be prevented from developing T2DM and intermediate hyperglycaemia annually, with a cost-saving of INT$132 million [13].

Given the need for context-appropriate interventions to prevent T2DM at scale, the 'Diabetes: Community-led Awareness, Response and Evaluation' (D:Clare) trial will further inform policy on population-level T2DM prevention and control, through a large-scale implementation of PLA in rural Bangladesh.

Methods

The aim of the D:Clare trial is to evaluate the scale-up of a PLA cycle intervention to prevent intermediate hyperglycaemia and T2DM and improve control of T2DM in rural Bangladesh.

We hypothesise that rapid horizontal scale-up of PLA will significantly (a) increase population-level awareness of diabetes prevention and control and (b) reduce the prevalence of intermediate hyperglycaemia and diabetes by at least 30%.

Setting

The trial is set in Alfadanga Upazila, Faridpur district, in the central region of Bangladesh. Faridpur has an estimated population of 1.7 million and is divided into nine upazilas. Alfadanga was purposefully selected as the trial location as it is less liable to flooding and land erosion than other areas of Faridpur, has not been exposed to a PLA intervention previously and is close to an existing field office. Alfadanga has an estimated population of 120,000 people, divided into six administrative unions. The area is rural with a predominantly agricultural economy based on jute and rice; the population is mainly Bengali and Muslim, reflecting a 'typical' rural setting in Bangladesh [14].

Primary healthcare is provided at union health centres, family welfare centres and community clinics. Secondary care is provided at the upazila health complex, a charity-based diabetes centre in Alfadanga town, and the District Hospital in Faridpur town. There are two tertiary referral hospitals in the Faridpur district, which can treat T2DM complications.

Design

This is a stepped-wedge cluster randomised controlled trial (SW-CRCT), with integrated process and economic

evaluations (Fig. 1). Clusters are defined as administrative geographical areas, with a population of approximately 10,000. Each of the six unions in Alfadanga will be divided into two, forming 12 clusters. The SW-CRCT is at risk of inter-cluster contamination, where participants residing in clusters under control conditions may be exposed to the intervention in a neighbouring cluster. Therefore, we will use a 'fried-egg' design for our evaluation surveys, with participants residing in border areas excluded from surveys.

The trial clusters will be randomly allocated, with a 1:1 ratio, to implement the intervention at either project month 1 or 12. The intervention will be evaluated with three cross-sectional surveys, at project months 1 (baseline), 12 (midline) and 24 (endline). The trial intervention period is planned to run for 30 months, from March 2020 to September 2022 (Fig. 1).

Population

The intervention will be delivered at the community level to the whole population of Alfadanga Upazila, Faridpur district. The intervention will be available and accessible to any community member to participate, including health care providers. We will particularly encourage high-risk individuals, those aged over 30 years and those with T2DM to attend, as the groups we expect to benefit most from the intervention. The evaluation will include permanent residents—those who have lived within the study cluster for a minimum of 6 months, aged 30 years and older. The evaluation will exclude pregnant women.

Intervention

The intervention is community mobilisation through PLA, focused on reducing intermediate hyperglycaemia and T2DM and improving non-communicable disease behaviours and risk factors. PLA works through community groups actively engaging communities in identifying the causes of health problems, and working together to design and implement ways to address these health problems, and reflect on their progress [15]. This approach has been shown to substantially improve maternal, neonatal and child health outcomes [16], and in the DMagic trial, reduce intermediate hyperglycaemia and T2DM [13].

The intervention and its theory base have been previously described [17–19]. Briefly, the intervention involves initiating separate men's and women's groups of 20–30 attendees. Separate groups are established to enable the participation of men and women that accounts for gender norms and convenient meeting times. Equal numbers of male and female groups are planned within communities, and groups will explore working together after approximately 10 months. The groups work through four phases of PLA: (1) problem identification where participants identify and prioritise causes of diabetes and diabetes risk in their community, (2) planning together where groups and communities collectively design strategies to address the causes of diabetes that can be implemented by communities, (3) strategy implementation and (4) participatory evaluation of the strategies which they have implemented. The four-phase cycle is planned to take 18 months, with groups meeting once a month. Those attending groups are encouraged to share information in communities and households and actively engage non-group attenders in their activities.

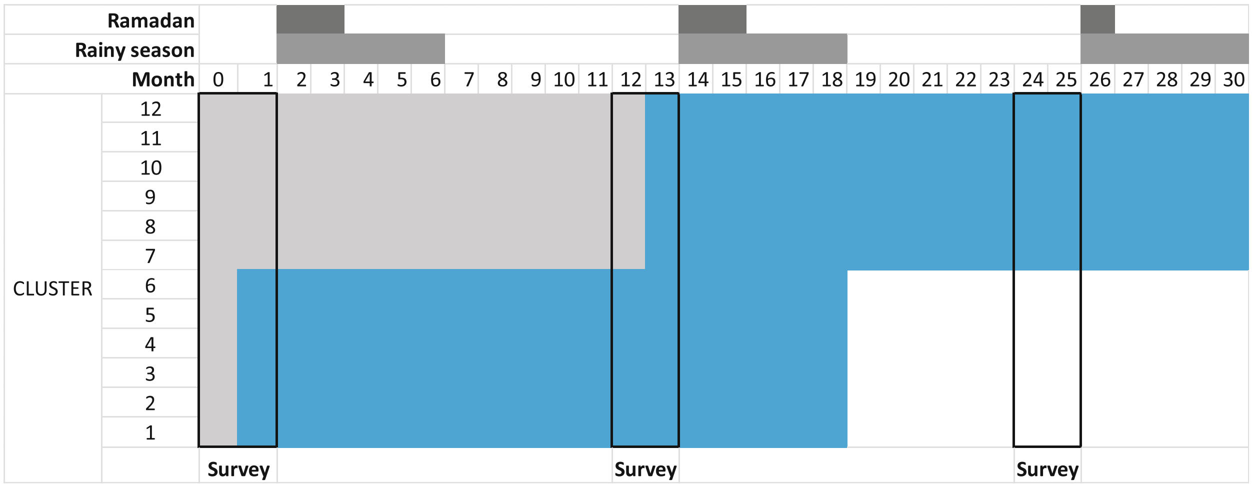

Blue = active intervention condition; Grey = control condition; White = after withdrawal of intervention

Fig. 1 Stepped-wedge trial design

We plan for nine men's and nine women's groups in each cluster ($n = 216$ groups), for a coverage of 1 group per 200 adults aged 30 years and above. The groups will be led by trained and salaried facilitators, with one male and one female facilitator in each cluster; they will facilitate approximately nine meetings per month. All facilitators will have a minimum of secondary school education and will be recruited from the intervention areas. A 1-week training will cover group facilitation and basic health messages related to T2DM prevention and control. The facilitators will be provided with a community action manual, picture cards and flip charts developed through formative research during the DMagic trial. These materials are aligned with standard recommendations for the prevention of T2DM provided by the Diabetic Association of Bangladesh. The male groups will be led by a male facilitator, and the female groups will be led by a female facilitator. Facilitators will be mentored and supported by two participatory group coordinators and a district manager, based in Alfadanga, and a senior group intervention manager based in Dhaka. At the end of 18 months, a group volunteer will be trained in facilitation, and the groups will be encouraged to continue meeting.

Control

During control periods, communities will receive standard diabetes prevention and care in accordance with usual practice in the region.

Outcome

The primary outcome is the combined prevalence of intermediate hyperglycaemia (i.e. impaired fasting glucose and/or impaired glucose tolerance) and T2DM amongst adults aged 30 years or older, based on WHO definitions (Table 1) [20]. This is the same definition used in the DMagic trial.

Table 1 WHO definition of normoglycaemia, intermediate hyperglycaemia and T2DM [20]

Definition	Diagnostic criteria
Normoglycaemia	Fasting plasma glucose ≤ 6.0 mmol/l
Intermediate hyperglycaemia	
Impaired fasting glucose	Fasting plasma glucose ≥ 6.1 mmol/l to < 7.0 mmol/l<br>AND<br>2-h post-ingestion of 75 g glucose load plasma glucose < 7.8 mmol/l
Impaired glucose tolerance	Fasting plasma glucose < 7.0 mmol/l<br>AND<br>2-h post-ingestion of 75 g glucose load plasma glucose ≥ 7.8 to < 11.1 mmol/l
Type 2 diabetes mellitus	Fasting plasma glucose ≥ 7.0 mmol/l<br>OR*<br>2-h post-ingestion of 75 g glucose load plasma glucose ≥ 11.1 mmol/l

Secondary outcomes include the following: self-awareness of diabetes status, smoking prevalence, physical activity, mean population diastolic and systolic blood pressure, prevalence of hypertension (systolic blood pressure ≥ 140 mmHg or a diastolic blood pressure ≥ 90 mmHg or current treatment with antihypertensive medication), mean population body mass index, prevalence of overweight and obesity, mean population waist and hip circumference ratio, consumption of sugar, dietary diversity, knowledge of diabetes symptoms and complications, utilisation of diabetic services, psychological distress and ability to self-manage amongst diabetics. The cumulative incidence of T2DM amongst individuals with intermediate hyperglycaemia, identified during the survey at month 1 and followed up in subsequent surveys (at 12 and 24 months), will be calculated. Primary and secondary outcomes will be measured at months 1, 12 and 24 (Fig. 1).

Randomisation

Due to the nature of the intervention, communities cannot be blinded to cluster allocation. Informed community consent will be taken through discussions with local leaders. Thereafter, clusters will be randomly allocated at a public meeting in the presence of traditional leaders and representatives from communities. The name of each cluster will be written on pieces of paper and folded uniformly by study staff, placed into a container and then drawn by a community representative. The clusters will be numbered in the order they are drawn, with clusters 1–6 implementing the intervention at month 1 and clusters 7–12 at month 12. The randomisation process will be filmed, with the consent of those present, to document the procedure.

Sampling

Participants in the impact evaluation will be selected using a three-step sampling approach. Prior to randomisation, evaluation villages used for all cross-sectional surveys will be purposively selected using the following criteria: they do not sit on a border with a neighbouring study cluster; they are not a major trading centre or administrative centre; they have a minimum of 50 households. Between two and five villages will be selected, to achieve a total of 800–1000 households per cluster based on estimated population sizes from the 2011 Bangladesh census, and confirmed through rapid study census (Table 2, Fig. 2).

A sampling frame of households with at least one eligible resident, aged over 30 years, within the evaluation villages will be generated, based on a study census completed in January 2020. A sample of 110 households will be selected using simple random sampling, with a random number generator in Stata SE 14. At the next stage,

Table 2 Estimated population per cluster, derived from the 2011 Bangladesh population census

Study cluster	Estimated cluster population	Estimated households in evaluation villages
Alfadanga 1	9891	923
Alfadanga 2	8827	1005
Bana 1	9345	970
Bana 2	8421	867
Buraich 1	11,386	1030
Buraich 2	13,047	813
Golpalpur 1	10,539	775
Golpalpur 2	9880	927
Panchuria 1	9257	706
Panchuria 2	9842	911
Tagarbanda 1	9628	812
Tagarbanda 2	10,450	736

a single eligible adult will be selected from each sampled household, using simple random sampling. At each survey, a new sample of households and individuals will be generated with replacement from the same sampling frame, i.e. some individuals and households may be sampled more than once by chance. Individuals identified with intermediate hyperglycaemia in the baseline survey will be purposefully sampled for inclusion in subsequent surveys.

Sample size

The sample size was calculated using a presumed baseline combined prevalence of intermediate hyperglycaemia and T2DM of 30%, an assumed exchangeable correlation structure across surveys for each cluster, and an estimated intra-cluster correlation of 0.07. A sample of 110 participants per cluster, per survey, will give 84% power at 95% confidence to detect a minimum of 30% relative reduction in disease prevalence to 21% or less. An additional 10% was added to the sample size to allow for non-response, resulting in a total target sample of 3960. We will, in addition, include individuals identified

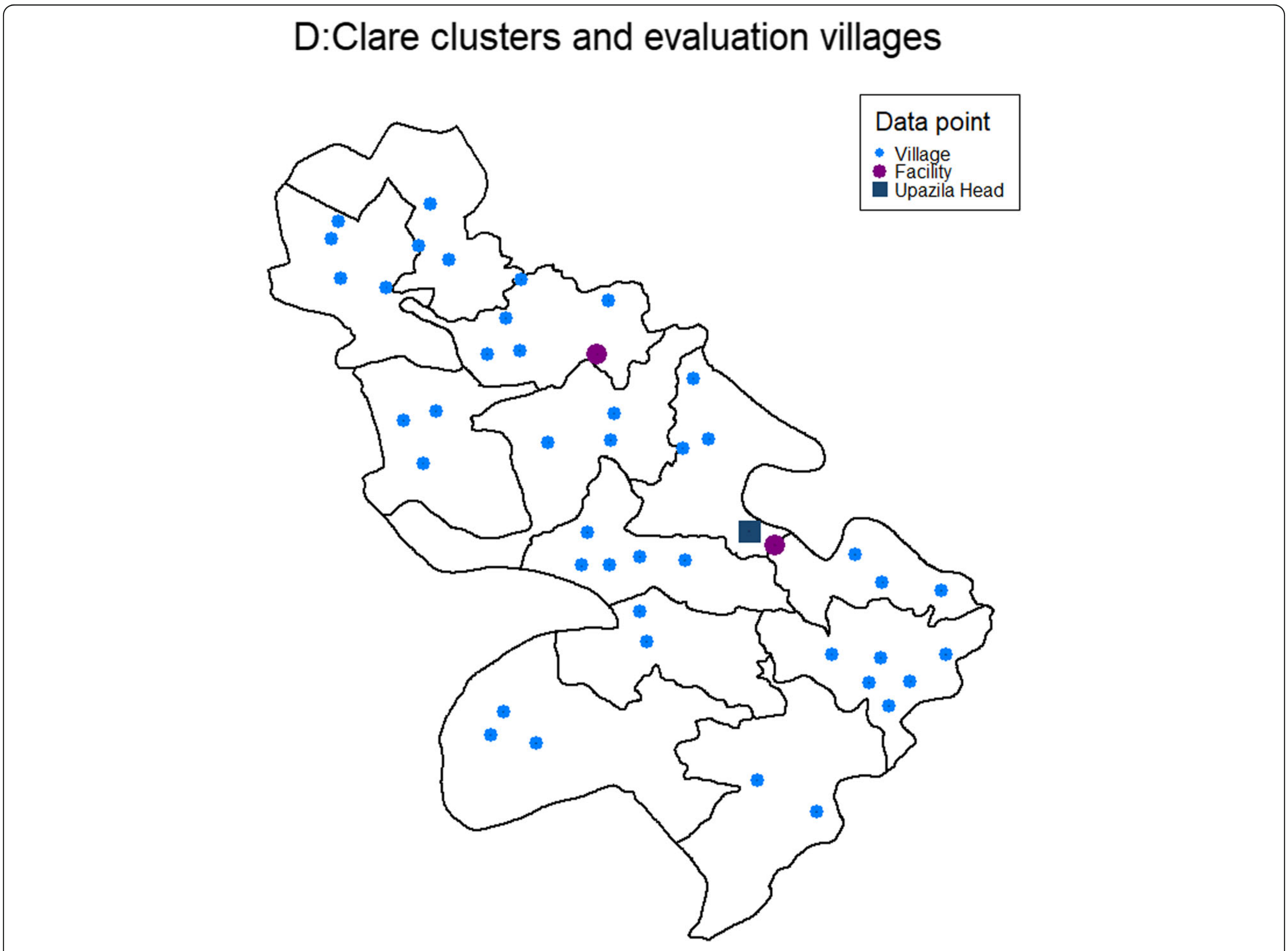

Fig. 2 Cluster and evaluation village map in Alfadanga Upazila

with intermediate hyperglycaemia in subsequent surveys. We estimate 360 individuals will be identified as having intermediate hyperglycaemia at baseline and included in the month 12 and 24 surveys. The sample size was calculated using Stata SE14.

Data collection

Data will be collected through three cross-sectional surveys at months 1, 12 and 24 (Fig. 1), by six pairs of fieldworkers (one male and one female) with three supervisors. Data collectors with completed secondary education will be recruited locally and selected following written assessment and interview. All fieldworkers will undergo 2 weeks of training, including consent, survey methods and physical measurements, followed by 1-week supervised field practice in villages outside of the study area. During training and field practice, survey tools will be piloted and revised. Within each village, teams will be aided by a village assistant, who will receive a daily payment to coordinate communication with study participants and assist data collectors. They will inform sampled individuals about the time and location of data collection, including the need to fast overnight. A convenient location within the village will be selected for the blood and anthropometric measures, and the collection of questionnaire data will take place at the respondent's home. All participants will be provided with verbal and written study information prior to taking part in anthropometric assessments and surveys, and data will only be collected following signed consent.

Individual, interviewer-administrated questionnaires will collect information on demographic and socioeconomic characteristics, risk behaviours, diabetes awareness indicators, health-seeking and costs of care. This questionnaire will be adapted from the WHO Stepwise tool [21] and the 2014 Bangladesh Demographic and Health Survey [14] and will include GAD-7 and PHQ-9 for mental health assessment [22, 23] and an adapted Appraisal of Diabetes Scale [24]. Blood pressure will be measured using the OMRON HBP 1100 Professional Blood Pressure Monitor (Kyoto, Japan). Two measurements will be taken with a 5-min interval, and the average will be taken for analysis. Height, weight, and waist and hip circumference will be measured, asking respondents to wear only light clothing and removing shoes. Blood glucose will be measured using the OneTouch Verio Flex Glucometer (Lifescan, Inc., Milpitas, CA 95035) in mmol/l, from the whole blood obtained by finger prick. Participants will be tested following an overnight fast of 8–12 h and then again 2-h after receiving a 75-g glucose load dissolved in 250 ml of water. In self-reported diabetics, we will take a random blood glucose measurement only.

Data will be collected using ODK Collect on Android tablets and downloaded via USB onto field supervisor's laptops and shared via a secure cloud server. Clusters, villages, households and individuals will be linked using unique study identifiers. Personal identifiable data will be collected in order to identify the sampled individuals in the community during surveys; however, these will be removed following cleaning for analysis and archiving. Data quality will be promoted through pilot testing of data collection tools, in-built range and logic checks, supervisor observation of data collection and concurrent data checking, with errors sent back to the field team for verification.

Analysis

The primary analysis will be an intention-to-treat analysis, at the individual level, comparing the combined prevalence of intermediate hyperglycaemia and T2DM between intervention and control periods across all clusters. All participants sampled during intervention periods will be included in the primary analysis, regardless of their direct engagement with the groups. The analysis will be based on regression models that include fixed effects for intervention exposure, survey timing, and sociodemographic predictors, and random effects for geographical clustering and, where appropriate, individual and household clustering (e.g. if the same individual or household is randomly sampled in two surveys). Participants with missing data on the primary outcome will be excluded from the analysis, as we anticipate high levels of participation. All estimates of the intervention effects will be presented with 95% confidence intervals. Data analysis will be done blinded, and clusters will only be unmasked following the presentation of the primary results to the Trial Steering Committee.

Outcome data in the intervention condition are collected at 12 and 24 months after the intervention is first implemented (Fig. 1). The primary analysis will however estimate one effect of the intervention, which assumes the effect of the intervention is the same after 12 and 24 months. To explore this assumption, we a priori plan to report the estimated intervention effects at these two durations separately, fitting a model that includes two intervention duration indicators. Intuitively, we can see the midline data allow the intervention effect after 12 months relative to control to be estimated, and the endline data allow the effect after 24 months relative to 12 months to be estimated. Combining these two will give the effect of the intervention after 24 months relative to control. Assuming that the intervention effect is the same at 18 months, when the intervention activities are completed, and 24 months (i.e. it does not 'tail off' once groups no longer have a paid facilitator), then the 24-

month effect can be interpreted as the 'full' intervention effect.

We will also explore subgroup analyses, by socioeconomic status, gender, age and for both intermediate hyperglycaemia and T2DM separately. As we have a small number of clusters involved in the trial, we are considering the use of random effects models with a correction for the modest number of clusters. This is an active area of methodological research, and a detailed final analysis plan will be written before the analysis begins.

Process evaluation

We will conduct a mixed methods process evaluation following the UK Medical Research Council guidelines to describe the intervention implementation and develop the theory of how the intervention can affect health outcomes in this context [25]. The intervention and trial design has used the extensive formative and process evaluation research from the DMagic trial [18, 19]. A process evaluation officer will collect qualitative data on how the intervention is working through (1) group observations throughout the intervention and (2) semi-structured and key informant interviews and group discussions in case study communities at two time points during the intervention. We will develop our hypothesis that health literacy is improved by the intervention [19] by integrating questions on health literacy in our survey tools [26]. We will also develop our hypothesis about the intervention effect on diabetes stigma through qualitative research and four quantitative questions asked to self-reported diabetics in the individual questionnaires. Quantitative data to describe intervention implementation will be collected from monthly reports of group supervisors who will observe an average of 33 meetings per month [18].

Economic evaluation

Cost and cost-effectiveness analyses will be conducted from both the health system and societal perspectives. Costs of implementing the intervention will be collected prospectively from the project accounts and input into a customised excel-based standardised costing tool designed for this purpose. Data on utilisation of NCD services from healthcare providers and costs of seeking care will be collected from the study participants during individual surveys. Incremental cost-effectiveness ratios (ICERs) will be evaluated in terms of the cost per case of intermediate hyperglycaemia and T2DM prevented and disability-adjusted life years (DALYs) averted. The robustness of the cost-effectiveness results will be assessed through a series of sensitivity analyses. In addition to cost-effectiveness analysis, an equity impact analysis will be conducted in order to assess whether the intervention has improved the equity of health service delivery and is improving the health status of those most in need. All costs will be estimated both from a financial and economic perspective and presented in international dollars.

Management and oversight

The project is a collaboration between the Diabetic Association of Bangladesh and University College London. A Trial Steering Committee will be convened according to the DAMOCLES charter, including a statistician, epidemiologist, social scientist and those with expertise in both community interventions and T2DM. This group will meet at key time points in the project (e.g. protocol review, endline results review), with the aim to meet a minimum of once a year [27]. Their role will be to provide an independent, objective review of the study implementation and baseline data and to advise on any extension or modification of the trial design. There are no stopping rules as we do not expect the intervention to have adverse effects at either cluster or participant level and have not planned an interim analysis to check for harm or futility. As a low-risk trial, a separate Data Monitoring Committee is not planned. The trial sponsor is the Institute for Global Health, University College London (30 Guildford Street, London WC1N 1EH, UK; +44 (0)20 7905 2352).

We will also establish community advisory boards, which include community representatives from Alfadanga. They will provide advice on the research design, implementation and support effective communication, and support feedback of adverse community events. These advisory boards will be consulted at key time points (e.g. group establishment and dissemination) and when questions or challenges arise (e.g. community conflicts). The Project Management Group, including all co-investigators, will meet at least monthly via teleconference, to review project progress, challenges and protocol compliance.

Dissemination

We plan to disseminate the impact, economic and process evaluation findings to an academic audience, policy-makers and study communities at the end of the project. Depending on the planned audience, we will hold meetings, workings and share reports, as appropriate. We will also publish results in open-access peer-reviewed journals.

Discussion

We present the protocol for a stepped-wedge cluster randomised trial, measuring the impact of a PLA intervention to prevent T2DM in a rural Bangladeshi setting at scale. During the research design process, we had extended discussions around three key methodological

challenges: SW-CRCT versus parallel-arm RCT, the timing of steps, and the number of sequences (i.e. ‘steps’).

Stepped-wedge versus parallel arm cluster RCT

The purpose of the D:Clare trial is to replicate the effective PLA approach used during the previous DMagic trial at scale. The CONSORT extension for SW-CRCT requests that authors justify using this study design, given the greater risk of bias compared to a traditional cRCT [28].

A replication study is generally defined as following the same methodology within a different study population or context; therefore, a cRCT design would be a direct replication of the DMagic trial. Given the PLA approach significantly reduced both incidence and prevalence of T2DM in the DMagic trial, but with no measurable impacts on most secondary outcomes, we felt it important to rigorously evaluate any scale-up and further explore the mechanisms of impact. As Alfadanga Upazila is in the same district as the previous DMagic population and does not have any unique distinguishing features to warrant a re-evaluation of the intervention theory, it could be argued this is not really a replication. Rather, it raises the question of whether we would meet the criteria of equipoise needed to justify an RCT.

The other factor to consider was the 36-month timeline of the project. The PLA intervention takes 18 months, and after scheduling for project setup, ethical approvals, data cleaning and analysis, there would not be sufficient time to run two full intervention cycles back to back. Therefore, on balance we selected the SW approach due to equipoise concerns, and achieving the overall aim of scale-up within the time available.

Timing of steps and data collection

As would be conventional for a SWT with cross-sectional data collection, we decided to collect data at the same time as the ‘steps’, i.e. when clusters switch to intervention, together with an endline survey. There is a theoretical basis that the time of year could influence not only the outcome assessment, i.e. fasting blood glucose and glucose tolerance, but also several secondary and explanatory variables, such as exercise and diet. As a rural population, farming practices, diet and work patterns are liable to change with the agricultural seasons. Therefore, surveys conducted during different seasons, with unequal proportions of data from intervention and control periods, could introduce bias. Such bias can potentially be removed at analysis, but the face validity of the trial would nevertheless be undermined. There are data showing seasonal variation in both fasting glucose and HbA1C amongst known T2DM [29–31]; however, we are not aware of any population data exploring this from South Asia. We therefore lack baseline knowledge on the direction of impact to comfortably adjust for this.

In addition, data collection during the rainy season and Ramadan present pragmatic challenges. Ramadan is also likely to have an important influence on diet and physical activity, with decreases in fasting blood glucose previously observed following Ramadan [32]. Therefore, we decided that data collection during these time periods would need to be avoided. A practical solution was to plan for all surveys during the same calendar period.

Number of sequences

Given the considerations about the timing of steps and project duration, we explored designs with 2, 3 and 4 sequences and having steps at 3 months, 6 months, 9 months or 12 months after the first implementation of the intervention—all scenarios feasible within 36 months. Based on the DMagic process evaluation, we reasoned that the community meeting at month 10 in the PLA cycle is an important milestone when groups start to implement strategies and when we might realistically expect to observe changes in population-level knowledge and behaviours. We therefore projected that some impact may be measured by month 12 of the intervention, but is less likely before. We discussed conducting the community meeting earlier in the PLA cycle, but had concerns about deviating from the effective DMagic intervention, and process data suggested groups need this initial period for problem analysis and building collective consciousness. We also hypothesised that the intervention effect may be larger beyond the end of the planned 18-month delivery, than at 12-months, given longer exposure to the intervention. Setting a 12-month period before expecting measurable intervention effects, designs with three or more sequences and hence four or more data collection surveys would have meant excluding clusters from intermediate surveys (or from the primary analysis) which had more recently implemented the intervention.

We therefore opted for the two-sequence design as the most pragmatic balance, estimating an overall (average) primary intervention effect and also the intervention effect after 12 months (6 months before completion) and 24 months (6 after completion), relative to control. Our major concern is that finding no effect may be a design artefact if PLA takes the full 18 months to work or effects are not sustained. If we assume that it takes 6 months for diabetes status to change, the measured intervention effect at 24 months should reflect the complete intervention exposure at 18 months. We will therefore give emphasis to the impact at 24 months, if its seen to be larger than the impact after 12 months. We acknowledge however that our power to estimate the 24-month effect will be lower than for the primary

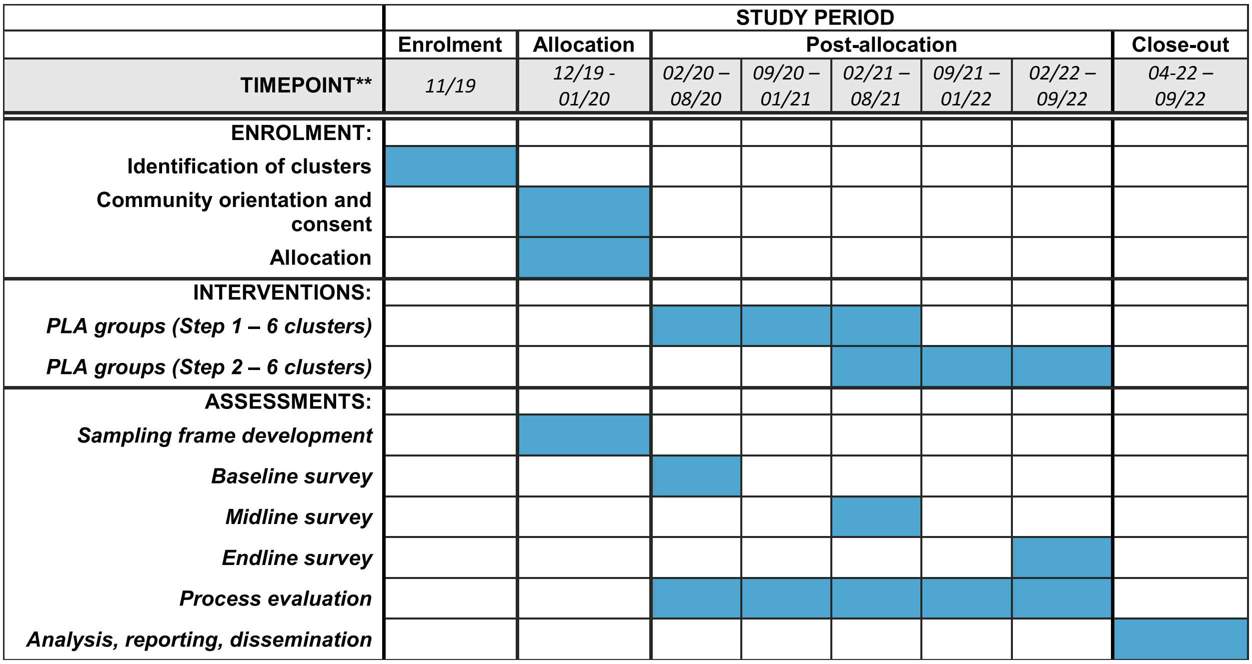

Fig. 3 Study time frame

effect. These pre-planned analyses of differential effects during the intervention period should provide important insights into the mechanisms and appropriate timeframe for intervention implementation in further scale-up.

Trial status

Protocol version 1.3 (10 December 2019).

The baseline survey started on 11 February 2020, and 52 of the 108 planned PLA groups in the first step have been established. Participant recruitment is anticipated to finish in March 2022 (Fig. 3). However, due to the COVID-19 pandemic, all intervention and data collection activities were paused on 20 March 2020 and as of 22 September 2020 have not resumed.

Abbreviations

D:Clare: Diabetes: Community-led Awareness, Response and Evaluation; DMagic: Diabetes Mellitus Action through community Groups or mHealth Information for better Control; LMIC: Low- and middle-income country; NCD: Non-communicable disease; PLA: Participatory learning and action; T2DM: Type 2 diabetes mellitus

Acknowledgements

We thank the members of our trial steering committee: Dr. David Beran (Chair), Prof. Graham Hitman, Prof. Sarah Hawkes, Prof. Anthony Costello, Prof. Edward Gregg, Dr. Jennifer Thompson, Prof. Audrey Prost and Prof. Justine Davies. We would also like to acknowledge the communities and community leaders for their engagement with the project.

Authors' contributions

EF is the principal investigator of the trial. He led the design of the study, wrote the first draft of the study protocol and will participate in the analysis and interpretation of the data. AK is the project manager of the trial, overseeing all activities. CK is a senior research associate of the project, supporting the design, analysis and interpretation of data and overseeing the data collection and management system. MP is a research assistant on the study, contributing to the design, analysis and interpretation of the data. JM is a senior research associate of the project, leading the design, analysis and interpretation of the process evaluation. HHB is a senior research associate of the project, leading the economic evaluation and equity components. AC is the trial statistician and contributed to the design of the study and will participate in the analysis and interpretation of data. NA contributed to the protocol development, fieldworker training and development of the interventions. SKS coordinates the survey data collection and processes and will participate in the analysis and interpretation of the data. TN contributed to the design of interventions and is responsible for the implementation of the community groups. KAk contributed to the design of the process evaluation. AKAK provides technical oversight. KAz is the project director, contributed to the design of the study, leads the implementation of the trial and will participate in the interpretation of the data. All authors read and approved the final manuscript.

Funding

The trial is funded by the UK Medical Research Council (ref: MR/T023562/1) under the Global Alliance for Chronic Diseases (GACD) Diabetes Programme. Peer review of the grant application provided input into the study proposal. Beyond that, the funder will have no role in the design of the study; the data collection, analysis and interpretation; or the write-up of the findings. Open Access funding provided by Karolinska Institute.

Availability of data and materials

Any data required to support the protocol can be supplied on request.

Declarations

Ethics approval and consent to participate

Written informed consent will be obtained from all survey and interview participants by study staff. Community consent will be sought from community leaders prior to randomisation and intervention implementation. Ethical approvals were provided by the University College London Research Ethics Committee (ref: 4199/007) and the Ethical Review Committee of the Diabetic Association of Bangladesh (ref: BADAS-ERC/E/19/00276). Any protocol modifications will be submitted for ethical review as amendments and updated in the trial registration.

Consent for publication
Not applicable.

Competing interests
The authors declare that they have no competing interests.

Author details
[1]Institute for Global Health, University College London, 30 Guildford Street, London WC1N 1EH, UK. [2]Department of Global Public Health, Karolinska Institutet, Stockholm, Sweden. [3]Diabetic Association of Bangladesh, Dhaka, Bangladesh.

Received: 19 October 2020 Accepted: 2 March 2021
Published online: 29 March 2021

References

1. Saeedi P, Petersohn I, Salpea P, Malanda B, Karuranga S, Unwin N, Colagiuri S, Guariguata L, Motala AA, Ogurtsova K, et al. Global and regional diabetes prevalence estimates for 2019 and projections for 2030 and 2045: results from the International Diabetes Federation Diabetes Atlas, 9th edition. Diabetes Res Clin Pract. 2019;157:107843.
2. International Diabetes Federation. IDF Diabetes Atlas. 2015;7. Accessed from: https://diabetesatlas.org/upload/resources/previous/files/7/IDF%20Diabetes%20Atlas%207th.pdf. Accessed 23 Mar 2021.
3. Ramachandran A, Snehalatha C, Shetty AS, Nanditha A. Trends in prevalence of diabetes in Asian countries. World J Diabetes. 2012;3(6):110–7.
4. Fottrell E, Ahmed N, Shaha SK, Jennings H, Kuddus A, Morrison J, Akter K, Nahar B, Nahar T, Haghparast-Bidgoli H, et al. Distribution of diabetes, hypertension and non-communicable disease risk factors among adults in rural Bangladesh: a cross-sectional survey. BMJ Glob Health. 2018;3(6):e000787.
5. Zheng Y, Ley SH, Hu FB. Global aetiology and epidemiology of type 2 diabetes mellitus and its complications. Nat Rev Endocrinol. 2018;14(2):88–98.
6. Gyawali B, Neupane D, Vaidya A, Sandbaek A, Kallestrup P. Community-based intervention for management of diabetes in Nepal (COBIN-D trial): study protocol for a cluster-randomized controlled trial. Trials. 2018;19(1):579.
7. Srinivasan K, Mazur A, Mony PK, Whooley M, Ekstrand ML. Improving mental health through integration with primary care in rural Karnataka: study protocol of a cluster randomized control trial. BMC Fam Pract. 2018;19(1):158.
8. Khetan A, Patel T, Hejjaji V, Barbhaya D, Mohan SKM, Josephson R, Webel A. Role development of community health workers for cardiovascular disease prevention in India. Eval Program Plann. 2018;67:177–83.
9. Kaur J, Kaur M, Webster J, Kumar R. Protocol for a cluster randomised controlled trial on information technology-enabled nutrition intervention among urban adults in Chandigarh (India): SMART eating trial. Glob Health Action. 2018;11(1):1419738.
10. Weber MB, Ranjani H, Meyers GC, Mohan V, Narayan KM. A model of translational research for diabetes prevention in low and middle-income countries: the Diabetes Community Lifestyle Improvement Program (D-CLIP) trial. Prim Care Diabetes. 2012;6(1):3–9.
11. Thankappan KR, Sathish T, Tapp RJ, Shaw JE, Lotfaliany M, Wolfe R, Absetz P, Mathews E, Aziz Z, Williams ED, et al. A peer-support lifestyle intervention for preventing type 2 diabetes in India: a cluster-randomized controlled trial of the Kerala Diabetes Prevention Program. PLoS Med. 2018;15(6):e1002575.
12. Adjaye-Gbewonyo K, Vaughan M. Reframing NCDs? An analysis of current debates. Glob Health Action. 2019;12(1):1641043.
13. Fottrell E, Ahmed N, Morrison J, Kuddus A, Shaha SK, King C, Jennings H, Akter K, Nahar T, Haghparast-Bidgoli H, et al. Community groups or mobile phone messaging to prevent and control type 2 diabetes and intermediate hyperglycaemia in Bangladesh (DMagic): a cluster-randomised controlled trial. Lancet Diabetes Endocrinol. 2019;7(3):200–12.
14. Research NIoP, Training - NIPORT/Bangladesh, Mitra, Associates, ICF International. Bangladesh demographic and health survey 2014. Dhaka: NIPORT, Mitra and Associates, and ICF International; 2016.
15. Rifkin SB and Pridmore P: Partners in planning: information, participation and empowerment. London: Macmillan Education Limited; 2001.
16. Prost A, Colbourn T, Seward N, Azad K, Coomarasamy A, Copas A, Houweling TAJ, Fottrell E, Kuddus A, Lewycka S, et al. Women's groups practising participatory learning and action to improve maternal and newborn health in low-resource settings: a systematic review and meta-analysis. Lancet. 2013;381(9879):1736–46.
17. Fottrell E, Jennings H, Kuddus A, Ahmed N, Morrison J, Akter K, Shaha SK, Nahar B, Nahar T, Haghparast-Bidgoli H, et al. The effect of community groups and mobile phone messages on the prevention and control of diabetes in rural Bangladesh: study protocol for a three-arm cluster randomised controlled trial. Trials. 2016;17(1):600.
18. Morrison J, Akter K, Jennings HM, Kuddus A, Nahar T, King C, Shaha SK, Ahmed N, Haghparast-Bidgoli H, Costello A, et al. Implementation and fidelity of a participatory learning and action cycle intervention to prevent and control type 2 diabetes in rural Bangladesh. Glob Health Res Policy. 2019;4(1):19.
19. Morrison J, Akter K, Jennings HM, Nahar T, Kuddus A, Shaha SK, Ahmed N, King C, Haghparast-Bidgoli H, Costello A, et al. Participatory learning and action to address type 2 diabetes in rural Bangladesh: a qualitative process evaluation. BMC Endocr Disord. 2019;19(1):118.
20. World Health Organisation, International Diabetes Federation. Definition and diagnosis of diabetes mellitus and intermediate hyperglycaemia: report of a WHO/IDF consultation. Geneva: World Health Organisation; 2006.
21. Bonita R, de Courten M, Dwyer T, Jamrozik K, Winkelmann R. Surveillance of risk factors for noncommunicable diseases: the WHO STEPwise approach. Summary. Geneva: World Health Organization; 2001.
22. Spitzer RL, Kroenke K, Williams JBW, Löwe B. A brief measure for assessing generalized anxiety disorder: the GAD-7. Arch Intern Med. 2006;166(10):1092–7.
23. Kroenke K, Spitzer RL. The PHQ-9: a new depression diagnostic and severity measure. Psychiatr Ann. 2002;32:509–15.
24. Carey MP, Jorgensen RS, Weinstock RS, Sprafkin RP, Lantinga LJ, Carnrike CLM, Baker MT, Meisler AW. Reliability and validity of the Appraisal of Diabetes Scale. J Behav Med. 1991;14(1):43–50.
25. Moore GF, Audrey S, Barker M, Bond L, Bonell C, Hardeman W, Moore L, O'Cathain A, Tinati T, Wight D, et al. Process evaluation of complex interventions: Medical Research Council guidance. BMJ. 2015;350:h1258.
26. World Health Organization ROfS-EA. Health literacy toolkit for low- and middle-income countries: a series of information sheets to empower communities and strengthen health systems. New Delhi: WHO Regional Office for South-East Asia; 2015.
27. NHS: Data Monitoring Committees in Clinical Trials: Guidance for research ethics committees. In. Edited by National Patient Safety Agency NRES. UK; 2010.
28. Hemming K, Taljaard M. Reflection on modern methods: when is a stepped-wedge cluster randomized trial a good study design choice? Int J Epidemiol. 2020;49(3):1043–52.
29. Tseng C-L, Brimacombe M, Xie M, Rajan M, Wang H, Kolassa J, Crystal S, Chen T-C, Pogach L, Safford M. Seasonal patterns in monthly hemoglobin A1c values. Am J Epidemiol. 2005;161(6):565–74.
30. Gikas A, Sotiropoulos A, Pastromas V, Papazafiropoulou A, Apostolou O, Pappas S. Seasonal variation in fasting glucose and HbA1c in patients with type 2 diabetes. Prim Care Diabetes. 2009;3(2):111–4.
31. Maguire G, Edwards O. Seasonal variation in glycated haemoglobin in diabetics. Ann Clin Biochem. 2001;38:56–60.
32. Kul S, Savaş E, Öztürk ZA, Karadağ G. Does Ramadan fasting alter body weight and blood lipids and fasting blood glucose in a healthy population? A meta-analysis. J Relig Health. 2014;53(3):929–42.

Publisher's Note
Springer Nature remains neutral with regard to jurisdictional claims in published maps and institutional affiliations.
