# Supplementary material for: A cluster randomised controlled trial of community groups using Participatory Learning and Action to prevent and control diabetes and intermediate hyperglycaemia in rural Bangladesh
Source: PLOS Glob Public Health. 2025 Aug 14;5(8):e0005049. doi: 10.1371/journal.pgph.0005049 (PMC12352636; doi:10.1371/journal.pgph.0005049)
Supplement: Attachment — Submitted filename: PGPH-D-24-01381_R1.pdf [file pgph.0005049.s015.pdf]

PLOS Global Public Health

A cluster randomised controlled trial of community groups using Participatory Learning and Action to prevent and control diabetes and intermediate hyperglycaemia in rural Bangladesh.

--Manuscript Draft--

**Manuscript Number:**	PGPH-D-24-01381R1
**Article Type:**	Clinical Trial
**Full Title:**	A cluster randomised controlled trial of community groups using Participatory Learning and Action to prevent and control diabetes and intermediate hyperglycaemia in rural Bangladesh.
**Short Title:**	The DClare trial of PLA to prevent and control diabetes in Bangladesh.
**Corresponding Author:**	Edward Fottrell<br>University College London<br>London, Not Listed or Not Applicable UNITED KINGDOM OF GREAT BRITAIN AND NORTHERN IRELAND
**Order of Authors:**	Edward Fottrell
	Abdul Kuddus, MPH
	Joanna Morrison, PhD
	Tasmin Nahar MSS
	Carina King, PhD
	Sanjit Kumer Shaha MSS
	Malini Pires, MPH
	Sarker Ashraf Uddin Ahmed, MPH
	James Beard, BSc
	Naveed Ahmed, MPH
	Andrew Copas, PhD
	Hassan Haghparast-Bidgoli, PhD
	A.K. Azad Khan
	Kishwar Azad, FCPS
**Keywords:**	Diabetes; intermediate hyperglycaemia; Hypertension; Community intervention; Bangladesh; cluster randomised controlled trial
**Abstract:**	Objectives<br>To evaluate the impact of Participatory Learning and Action (PLA) community mobilisation intervention for diabetes prevention and control when implemented in a new area in rural Bangladesh and in the context of COVID-19.<br>Design<br>Cluster randomised controlled trial.<br>Setting<br>Rural communities in Alfadanga Upazila, Faridpur District, Bangladesh.<br>Participants<br>Adults aged >30 years who permanently reside in study villages. A random cross-sectional sample of 1392 adults at baseline (pre-intervention) and 1343 at endline (post-intervention) was selected to evaluate intervention impact.<br>Intervention<br>Community mobilisation through PLA, which works through facilitated community groups actively engaging communities in identifying the causes of health problems, and working together to design and implement ways to address these health problems, and reflect on their progress. One hundred and eight PLA groups were created with the intention to run for 18 months, with groups meeting once a month. Due to COVID-

Powered by Editorial Manager® and ProduXion Manager® from Aries Systems Corporation

	related interruptions, however, implementation was modified to allow a minimum of 13 meetings held over a total implementation period of 20 months.<br>Main outcome measures<br>Prevalence of intermediate hyperglycaemia and diabetes assessed through fasting blood glucose concentrations and two-hour oral glucose tolerance tests. Secondary outcomes included blood pressure, knowledge of diabetes and behavioural outcomes, including diet, physical activity, and care-seeking.<br>Results<br>There was no evidence of an intervention effect on the primary outcome of prevalence of intermediate hyperglycaemia and diabetes between study arms (adjusted difference -1.19% (95% CI -10.74, 8.36), p=0.784). Large increases in diabetes knowledge were recorded and the intervention was associated with a significant reduction in mean diastolic blood pressure (-2.98 (-5.55, -0.41), p=0.028) and possible reductions in the prevalence of hypertension (-7.40% (-16.03, 1.24), p=0.0845) and abdominal obesity (-15.23% (-33.02, 2.56), p=0.085).<br>Conclusion<br>After a total of 20 months of intervention delivery there was no evidence of impact of PLA on blood glucose outcomes, but large impacts on diabetes knowledges were seen and likely impacts on some health outcomes, including mean diastolic blood pressure. Interruptions to implementation of PLA for diabetes prevention and control during the evolving COVID-19 pandemic required a reduction in the total number of group meetings and other adaptations that may have affected intervention effectiveness. Nevertheless, the PLA approach to non-communicable disease prevention and control continues to show promise in resource-poor settings.<br>Funding<br>This work was funded by the Medical Research Council UK (MR/T023562/1) under the Global Alliance for Chronic Diseases Scale-Up Programme.<br>Trial registration: ISRCTN42219712 – registered 31st October 2019. Status: complete.
**Additional Information:**	
**Question**	**Response**
**Financial Disclosure**<br><br>Enter a financial disclosure statement that describes the sources of funding for the work included in this submission and the role the funder(s) played. This includes grants and any commercial funding of the work or authors.<br><br>This statement will be typeset if the manuscript is accepted for publication.<br><br>*Please review the submission guidelines and the instructions link below for detailed requirements and guidance.*	This work was funded by the Medical Research Council UK (MR/T023562/1) under the Global Alliance for Chronic Diseases Scale-Up Programme. The funder of the study had no role in study design, data collection, data analysis, data interpretation, or writing the report.
**Competing Interests**<br><br>On behalf of all authors, disclose any competing interests that could be perceived to bias this work.	The authors have declared that no competing interests exist.

This statement will be typeset if the manuscript is accepted for publication.<br><br>*Please review the instructions link below and PLOS Global Public Health's competing interests policy to determine what information must be disclosed at submission.*	
**Data Availability**<br><br>Before publication, Authors are required to make fully available and without restriction all data underlying their findings. Please see our PLOS Data Policy page for detailed information on this policy.<br><br>A **Data Availability Statement**, detailing where the data can be accessed, is required at first submission. Insert your Data Availability Statement in the box below.<br><br>Please see the data reporting section of our submission guidelines for instructions on what you need to include in your Data Availability Statement.<br><br>This statement will be typeset if the manuscript is accepted for publication.<br><br>*PLOS allows rare exemptions to address legal and ethical concerns. If you have legal or ethical restrictions, please use the box below to detail these in full sentences for the Journal team to consider.*	Deidentified data collected for this study and a data dictionary are available from the UCL Research Data Repository DOI:10.5522/04/28751210.

Manuscript
Click here to access/download;Manuscript;20250408_DClareTrialPaper.doc

A cluster randomised controlled trial of community groups using Participatory Learning and Action to prevent and control diabetes and intermediate hyperglycaemia in rural Bangladesh.

Short Title: The DClare trial of PLA to prevent and control diabetes in Bangladesh.

Edward Fottrell PhD[1*] (0000-0003-0518-7161), Abdul Kuddus MPH[2], Joanna Morrison PhD[1], Tasmin Nahar MSS[2], Carina King PhD[3], Sanjit Kumer Shaha MSS[2], Malini Pires MPH[1], Sarker Ashraf Uddin Ahmed MPH[2], James Beard BSc[4], Naveed Ahmed MPH[2], Andrew Copas PhD[1], Hassan Haghparast-Bidgoli PhD[1], A.K. Azad Khan D.Phil[2], Kishwar Azad FCPS[2]

1 University College London Institute for Global Health, London, United Kingdom
2 Centre for Health Research and Implementation, Diabetic Association of Bangladesh, Dhaka, Bangladesh
3 Department of Global Public Health, Karolinska Institutet, Stockholm, Sweden
4 Independent Consultant, Guildford, United Kingdom

*Correspondence to: Edward Fottrell e.fottrell@ucl.ac.uk

Author contributions: EF was Principal Investigator, wrote the first draft, conducted analyses and led the design of the study. AK oversaw the implementation of implementation and evaluation activities; JM led the process evaluation and drafted relevant parts of the manuscript; TN was responsible for intervention implementation; CK contributed to study design and led in data processing; SKS led evaluation and survey activities; MP contributed to study design and process evaluation; SAUA implemented process evaluation research activities; JB contributed to data processing; NA contributed to intervention and survey design; AC was the trial statistician; HHB led the economic evaluation; AKAK and KA facilitated project activities and contributed to project design and implementation. All authors contributed to interpretation of the data and revisions of the manuscript.

Abstract

Objectives

To evaluate the impact of Participatory Learning and Action (PLA) community mobilisation intervention for diabetes prevention and control when implemented in a new area in rural Bangladesh and in the context of COVID-19.

Design

Cluster randomised controlled trial.

Setting

Rural communities in Alfadanga Upazila, Faridpur District, Bangladesh.

Participants

Adults aged ≥30 years who permanently reside in study villages. A random cross-sectional sample of 1392 adults at baseline (pre-intervention) and 1343 at endline (post-intervention) was selected to evaluate intervention impact.

Intervention

Community mobilisation through PLA, which works through facilitated community groups actively engaging communities in identifying the causes of health problems and working together to design and implement ways to address these health problems, and reflect on their progress. One hundred and eight PLA groups were created with the intention to run for 18 months, with groups meeting once a month. Due to COVID-related interruptions, however, implementation was modified to allow a minimum of 13 meetings held over a total implementation period of 20 months.

Main outcome measures

Prevalence of intermediate hyperglycaemia and diabetes assessed through fasting blood glucose concentrations and two-hour oral glucose tolerance tests. Secondary outcomes included blood pressure, knowledge of diabetes and behavioural outcomes, including diet, physical activity, and care-seeking.

Results

There was no evidence of an intervention effect on the primary outcome of prevalence of intermediate hyperglycaemia and diabetes between study arms (adjusted difference -1.19% (95% CI -10.74, 8.36), p=0.784). Large increases in diabetes knowledge were recorded and the intervention was associated with a significant reduction in mean diastolic blood pressure (-2.98 (-5.55, -0.41), p=0.028) and possible reductions in the prevalence of hypertension (-7.40% (-16.03, 1.24), p=0.0845) and abdominal obesity (-15.23% (-33.02, 2.56), p=0.085).

Conclusion

After a total of 20 months of intervention delivery there was no evidence of impact of PLA on blood glucose outcomes, but large impacts on diabetes knowledges were seen and likely impacts on some health outcomes, including mean diastolic blood pressure. Interruptions to implementation of PLA for diabetes prevention and control during the evolving COVID-19 pandemic required a reduction in the total number of group meetings and other adaptations that may have affected intervention effectiveness. Nevertheless, the PLA approach to non-communicable disease prevention and control continues to show promise in resource-poor settings.

Funding

This work was funded by the Medical Research Council UK (MR/T023562/1) under the Global Alliance for Chronic Diseases Scale-Up Programme.

Trial registration: ISRCTN42219712 – registered 31st October 2019. Status: complete.

Introduction

Globally, an estimated 537 million adults aged 20-79 have diabetes and more than three quarters of these people live in low- and middle-income countries.[1-3] By 2045, the International Diabetes Federation predicts that 1 in 8 adults in this age range – approximately 783 million – will be living with diabetes, an increase of 46% and with the largest increases occurring in resource-poor settings.[3] The majority of people living with diabetes in low- and middle-income settings are undiagnosed and unaware of their condition, and health systems in resource-poor settings are ill-equipped to respond to the growing burden of diabetes risk and morbidity.[4] There is therefore a pressing need for effective, scalable and sustainable population-level initiatives to raise awareness and to prevent and control diabetes globally, and particularly in resource-poor settings.

In Bangladesh, the prevalence of diabetes is estimated to range from 2% to 13% and the estimated prevalence of intermediate hyperglycaemia (impaired fasting glucose or impaired glucose tolerance indicating an increased risk of diabetes) varies between 2% and 22%.[5] Between 2015 and 2018, the DMagic (Diabetes Mellitus Action through community Groups or mHealth Information for better Control) trial used a participatory learning and action (PLA) approach for type-2 diabetes mellitus (T2DM) prevention and control directed at the general population in rural Bangladesh.[6] After 18 months of implementation of 122 monthly PLA meetings, the trial showed significant increases in diabetes knowledge and awareness and large reductions in the prevalence of diabetes and intermediate hyperglycaemia, with an absolute reduction of -20.1% (95% CI:-26.1, -14.0).[6, 7] Among individuals identified with intermediate hyperglycaemia, the two-year cumulative incidence odds of diabetes was 59% lower (OR: 0.41; 95% CI:0.24, 0.67), equating to an absolute reduction of -8.4% (95% CI: -13.8, -2.99).[6] Impact was shown to be highly cost effective, equitable and,[8] though diabetes outcomes were no longer observed at 5-years post randomisation, other longer-term potential health impacts were notable, including reductions in population hypertension outcomes.[9]

Given the need for context-appropriate interventions to prevent diabetes at scale, the Diabetes: Community-led Awareness, Response and Evaluation (D:Clare) trial assesses the impact of PLA on T2DM and other non-communicable disease and risk outcomes when implemented across a sub-district (‘upazila’) in Bangladesh and the context of COVID-19, with pragmatic adaptations to intervention delivery and evaluation.[10, 11]

Methods

Setting

The D:Clare trial took place between January 2020 and December 2022 in Alfadanga upazila, Faridpur district, Bangladesh. Alfadanga was purposefully selected as the trial location as it had not been exposed to a PLA intervention previously and is close to an existing field office. Alfadanga has an estimated population of 120,000 people, divided into six administrative unions and has a predominantly agricultural economy.[12]

Study design and participants

Originally designed as a stepped-wedge cluster randomised controlled trial[11] we changed our design to a parallel-arm two group cluster RCT with 1:1 allocation[10] due to COVID-19-related project interruptions and potential impacts on health and contextual factors.[13]

Each of the six *unions* in Alfadanga were split in two, forming 12 clusters (population approx.=10,000 each). Evaluation was based on data collected at baseline (post randomisation but pre-intervention delivery) and endline (post intervention) cross-sectional surveys. To minimise inter-cluster

contamination, we employed a 'fried-egg' design for our evaluation surveys, with participants residing in border areas excluded from surveys. Everyone in intervention clusters was eligible to participate in the intervention but evaluation was restricted to non-pregnant female and male permanent residents aged 30 years and above.

Ethics

The trial received ethical approval from University College London (4199/007) and the Diabetic Association of Bangladesh (BADAS-ERC/E/19/00276). All survey participants indicated informed consent through signature or thumbprint.

Randomisation and masking

Randomisation took place at a project orientation meeting in Faridpur town attended by community representatives and independent observers. Twelve folded pieces of paper, each with a name one of the clusters, were placed into a container and then drawn by meeting participants. The first six clusters were allocated to intervention, and the subsequent six were allocated to control.

Intervention

Participatory Learning and Action

The intervention was community mobilisation through PLA, which works through facilitated community groups actively engaging communities in identifying the causes of health problems, and working together to design and implement ways to address these health problems, and reflect on their progress.[14]

One hundred and eight PLA groups, 9 men's and 9 women's in each intervention cluster - approximately 1 per 200 adults aged 30 years and above – were created with the intention to work through four phases of PLA: 1) problem identification where participants identify and prioritise causes of T2DM and T2DM risk in their community; 2) planning together where groups and communities collectively design strategies to address the causes of T2DM that can be implemented by communities; 3) strategy implementation; 4) participatory evaluation of the strategies which they have implemented. The four-phase cycle was planned to take 18 months, with groups meeting once a month. Due to COVID-related interruptions, however, implementation was modified to allow a minimum of 13 meetings held over a total implementation period of 20 months. Additional COVID-related modifications to the intervention delivery are summarised in Supplementary Table 1. Those attending groups were encouraged to share learning and key messages with other members of the community who were unable to attend.

PLA groups were led by 12 facilitators (6 males, 6 females) – two in each cluster – recruited from the intervention areas. They received training on group facilitation and basic health messages related to non-communicable disease prevention and control, with particular focus on T2DM. Facilitators were equipped with a picture card, flip chart and PLA manual with topic-specific information and suggested group activities. The intention was for groups to cover one topic in each meeting, but due to COVID-19 adaptations, multiple topics needed to be covered in single meetings. Topics included information on T2DM, its causes, symptoms, prevention, and control. The PLA manual was adapted from the DMagic trial, aligned with standard recommendations for awareness, prevention and control of T2DM and updated to incorporate information about preventing COVID-19. Male groups were led by a male facilitator and female groups were led by a female facilitator. Facilitators were salaried and each was expected to coordinate and facilitate an average of 18 group meetings per month. Facilitators were mentored and supported by two participatory group coordinators and, a District Manager based locally, and a Senior Group Intervention Manager based in Dhaka.

Piloting & interruption

We conducted a 1-month pilot in intervention areas in December 2020. Experiences from the pilot were shared with study Community Advisory Groups – groups based in each of the study upazilas

and comprised of community stakeholders – who were asked to provide input on the acceptability of implementing the PLA intervention after COVID-19 lockdown. Details of this community consultation are published elsewhere and describe key adaptations to the intervention, including restrictions on group size and the implementation of COVID-19 screening and hygiene measures.[13] Routine intervention implementation began in January 2021. A further government COVID-19 lockdown was imposed between April 2021 and August 2021, during which time all field activities, including intervention implementation were paused. Intervention implementation resumed in November 2021 and then continued uninterrupted until September 2022.

Control

The 6 control clusters received usual care, which in this context is little or no preventative public health campaigning and care seeking in government or private facilities, often associated with out-of-pocket payment for diagnostics, consultations, and treatments. Commitment to deliver the intervention to all clusters was made at the public orientation meeting (reflecting the original stepped-wedge design). Control clusters started to receive the intervention after the trial evaluation endline survey in November 2022.

Procedures

Sampling for evaluation

We used a three-step sampling approach. Prior to randomisation, we purposefully selected two to six evaluation villages from a central area in each cluster, with the aim to have between 800-1000 households present, and assuming an average of two people aged $\geq$30 years in each household. Eligible villages were those which do not sit on a border with a neighbouring study cluster, do not act as a major trading centre or administrative centre, and had a minimum of 50 households. The list of villages, and their estimated population sizes was derived from the 2011 Bangladesh census, and administrative maps.

A sampling frame of all the households with at least one non-pregnant permanent resident aged 25 years or above within the selected evaluation villages was generated by household census completed in the evaluation villages in January 2020. Each member of the household aged 30 years or older was listed and a sample of households with at least one adult aged $\geq$30 years was selected using simple random sampling. Next, a one adult aged $\geq$30 years was selected for inclusion in the survey, again using simple random sampling. New samples of households and individuals were generated from the same sampling frame (same villages) for the baseline survey (4th January to 25th February 2021) and endline survey (5th September and 15th November 2022). This general population sample was used to evaluate population-wide intervention impacts.

In addition, individuals aged 30 years or older identified with intermediate hyperglycaemia in the 2021 baseline survey were followed-up at endline (i.e. an intermediate hyperglycaemia cohort) and this sample was used to evaluate intervention impacts on diabetes incidence in a high-risk population. Therefore, we describe the total endline collection as including a cross-sectional random population sample, and a cohort sample of those with intermediate hyperglycaemia at baseline. Some individuals with baseline intermediate hyperglycaemia were also randomly sampled in the cross-sectional survey at endline – these individuals were considered to belong to both samples.

Survey Procedures

All sampled individuals in a single village were informed of the requirements of the study and were requested to prepare for data collection in the morning of a specified day following an overnight fast. Data collection took place at respondents' homes or at a convenient location in their village.

Data were collected by six teams of locally recruited fieldworkers comprised of one male and one female with at least secondary education. They underwent 10 days training on survey methods and how to take physical measurements, followed by one-week supervised field practice and piloting in

non-study villages in Faridpur district. Data collectors were supervised by two field supervisors with experience in survey methods. Questionnaire data were gathered in Bangla on Android tablets using ODK Collect.

Individual Questionnaire

Fieldworkers used a structured survey instrument adapted from the WHO Stepwise tool[15] and 2014 Bangladesh Demographic and Health Survey[12] to measure the background demographic and socio-economic characteristics, lifestyle and behavioural risk factors, T2DM awareness indicators and health seeking behaviour and costs of care seeking among study participants. Given the established links between diabetes, depression[16] and anxiety, we used the Patient Health Questionnaire-9 (PHQ-9) and General Anxiety Disorder-7 (GAD-7) mental health screening tools to measure depression and anxiety, respectively.[17, 18] Both PHQ-9 and GAD-7 have been applied previously in Bangladesh and similar populations.[19, 20]

Physical measurements

Fieldworkers measured blood glucose using the One Touch Verio Flex Glucometer (Lifescan, Inc., Milpitas, USA) in whole blood obtained by finger prick from capillaries in the middle or ring finger. Individuals with a self-reported prior medical diagnosis of T2DM were invited to provide random blood glucose readings whereas all other participants were asked to provide readings after an over-night fast. All individuals without a prior diagnosis of T2DM then received a 75g glucose load dissolved in 250 ml of water and had a repeat capillary blood test within 5 minutes of 120 minutes post ingestion to determine glucose tolerance status and differentiate between individuals with intermediate hyperglycaemia and those with T2DM according to WHO criteria (Supplementary Table 2).

Blood pressure was measured using the OMRON HBP 1100 Professional Blood Pressure Monitor (Kyoto, Japan). Two measurements were taken at approximately 5-minute intervals and the respondent's blood pressure obtained by averaging these measurements. Measurements of height, weight, and waist and hip girth were taken with light clothes without shoes and using standard methods.[21] Survey participants were provided with their blood glucose and blood pressure results and were signposted to health services for further testing, advice and treatment.

Data Management & Quality Control

Data quality control was ensured through ODK form design, supervisor observation, and data checking in Dhaka, after which data were uploaded to a cloud server where project data leads (King/Beard) ran Stata scripts for further data verification.

The same procedures were applied at the baseline and endline surveys. Data collectors, supervisors and managers were unaware of randomisation assignments at baseline but field staff may have been able to deduce assignment during data collection at endline. Access to endline data was restricted until collection was complete, at which point the data were available for blinded analysis by the PI (EF) and trial statistician (AC). Data collection, management and analytical procedures were monitored by an independent Data Monitoring Committee (DMC). Trial management was also reviewed by an independent Trial Steering Committee (TSC).

Process evaluation

Process evaluation data has been published in detail elsewhere.[22] Briefly, we conducted a mixed methods process evaluation following the MRC guidelines to describe the context of intervention and control areas, implementation of the intervention, and mechanisms of how the intervention might have affected health outcomes. In intervention areas, we used project monitoring documents, group meeting minutes, observations, interviews and focus group discussions with the intervention team and group members.[23] Qualitative interviews and discussions were conducted by a process

evaluation officer, transcribed and translated to English and analysed using the framework approach.[24]

Outcomes

Our primary outcome was the combined prevalence of intermediate hyperglycaemia (i.e. impaired fasting glucose or impaired glucose tolerance) and T2DM among adults aged 30 years or older. We used WHO definitions and blood glucose cut-offs for normoglycaemia, impaired fasting glucose, impaired glucose tolerance and T2DM as summarised in Supplementary Table 2. Individuals with a self-reported prior diagnosis of T2DM were classified as living with diabetes in our analysis, irrespective of blood glucose readings. The primary outcome is defined only for those in the cross-sectional sample at endline.

Secondary outcomes are defined in Supplementary Table 3 and relate to self-awareness of diabetic status, knowledge of T2DM, utilisation of diabetic services, depression, anxiety and common non-communicable disease risk factors. We also calculated the cumulative incidence of T2DM among the cohort of individuals with intermediate hyperglycaemia.

We also included several pre-specified exploratory and explanatory outcome measures designed to identify potential mechanisms of intervention effect on population behaviours, health and wellbeing. Among individuals with a self-reported prior diagnosis of T2DM, these included: diabetes control (random blood glucose $\leq$11.1 mmol/L), self-reported diabetes-related health complications, at least monthly blood glucose testing, perceptions of social stigma, measures of family support or abuse related to diabetes diagnosis, and psychological distress and ability to self-manage diabetes assessed using the Appraisal of Diabetes Scale (ADS).[25] Among all participants, we measured self-rated health (score 0-100 on a visual analogue scale), tobacco use, betel nut use, sedentary time, time engaged in brisk walking within the previous 7 days, and perceptions of social norms related to dietary practices and female physical activity.

Sample size and statistical analysis

Due to COVID-19 adaptations, we updated our sample size calculation in 2022 using methods described by Copas and Hooper (2020).[26] With an average of 116 participants per cluster in the baseline survey (n=1392), we estimated that 125 participants per cluster in the endline survey would give 78% power to detect a 30% reduction in the primary outcome of combined prevalence of T2DM and intermediate hyperglycaemia, assuming a baseline primary outcome prevalence of 40%, an intra-cluster correlation of 0.02 and an estimated autocorrelation between baseline and endline of 0.4. We therefore sampled 132 respondents for the endline survey, to account for non-participation (n=1584). Our goal was to also collect data from a cohort of participants with intermediate hyperglycaemia at baseline, giving an endline target sample of 1868.

The main analysis of the primary outcome includes all individuals who provided blood glucose measures at endline from the cross-sectional survey. For the cumulative incidence secondary outcome, analysis includes all individuals in the cohort for whom a baseline blood glucose measurement of intermediate hyperglycaemia was taken and an endline blood glucose measurement was taken. We did not impute any values for missing data since key predictors we might use for imputation (e.g. age, sex, baseline hyperglycaemia) were already included in model adjustments.

The primary analyses for each outcome were undertaken on an intention-to-treat basis. For each outcome, unless otherwise specified, we conducted an ‘unadjusted’ and ‘adjusted’ analysis. All analyses included baseline data alongside the endline data to gain precision and account for any baseline imbalance. The ‘unadjusted’ analysis included adjustment only for the baseline prevalence of the outcome at the cluster level. ‘Adjusted’ analysis included further adjustment for age as a

linear and quadratic term, gender, and baseline hyperglycaemia, as specified *a priori* as likely predictors of intermediate hyperglycaemia and T2DM.

We applied a two-stage cluster summary approach for all outcomes because of the modest number of clusters.[27] In the first stage of the unadjusted analysis a cluster summary of the outcome was calculated for each cluster at baseline and endline. In the second stage the endline cluster summary values were analysed using linear regression including trial arm and baseline cluster summary value as predictors. The coefficient for trial arm represents an intervention effect on the difference scale.

For the adjusted analysis, we first calculated adjusted cluster summary values at baseline and endline by, separately for each time point, fitting a model to predict the outcome including as predictors age, gender and (for endline model only) baseline survey participation, but excluding trial arm and ignoring the clustering in the data. The outcome was then predicted for all participants (as a probability for binary outcomes) and the cluster mean of the predicted values was calculated for all clusters. Next a residual term was calculated for each cluster – the observed cluster mean minus the mean predicted value. In the second stage the residuals at endline were modelled using linear regression including trial arm and baseline residual as predictors. The coefficient for trial arm represents the adjusted intervention effect on the difference scale. We consider the adjusted analysis primary for all outcomes.

Primary analysis of the primary outcome was conducted blind and independently by the trial PI (EF) and trial statistician (AC) who reported these results to the DMC and Chair of the TSC, after which the identities of the trial arms were revealed, and analysis continued un-blinded.

Pre-specified exploratory analyses of possible interactions between intervention and the following characteristics were undertaken using individual level analysis of endline data: (1) gender, (2) household wealth, (3) age, (4) village size (less than mean village size vs. greater than or equal to mean village size), and (5) inclusion in baseline survey. Specifically, we used independence estimating equations with robust standard errors through the use of the complex survey 'svy' functions in Stata and added the interaction term between allocated group and subgroup variable into a logistic regression (odds) model.

To assess effects of possible misclassification in the primary outcome based on self-reported diagnoses, we also conducted sensitivity analysis of intervention effect on the primary outcome whereby a) all cases of self-reported T2DM were dropped from the analysis and b) all cases were defined based on blood glucose measures only (i.e. disregarding self-reported diagnoses) where individuals with self-reported diabetes with random blood glucose values of $\geq$11.1 mmol/L were classified as living with diabetes and those with values <11.1 mmol/L were classified as normoglycaemic.

To assess effects of possible blood glucose measurement bias we assessed intervention impact on continuous blood glucose measurements and by separately applying different arbitrary fasting blood glucose cut-offs of 5·5 mmol/L, 6·3 mmol/L, and 7·8 mmol/L and 2-h blood glucose cut-offs of 6·8 mmol/L and 10·4 mmol/L for classifications of intermediate hyperglycaemia or T2DM.

Total cost and cost-effectiveness analysis of the PLA intervention was conducted from a programme provider perspective. Costs were collected prospectively from project accounts and input into a customised excel-based costing tool designed for this purpose. Time horizon for costing was 36 months, including 16 months start-up and 20 months implementation. Costs were estimated. The incremental cost-effectiveness ratio (ICER) was calculated in terms of cost per 1-mmHg reduction in diastolic blood pressure (statistically significant secondary outcome). All costs were adjusted for inflation, discounted at 3% per year and converted to 2022 US dollar (US$) using the exchange rate of 1US$=91.75 Bangladeshi Taka.[28] A detailed analysis will be presented in a separate publication.

Statistical analysis was conducted in StataSE version 15 and StataMP version 18, and a 5% significance level used for all analyses.

Patient & public involvement

Members of the public and community representatives were engaged in a community orientation meeting at the start of the study and in regular community advisory groups to inform the processes and methods used in the study. This included representatives from each of the study upazillas acting as a link between the project team and communities and providing direct input into the design of study tools and methods, intervention delivery (including interruptions and re-starting PLA groups in the context of COVID-19[13]), and, at the end of the trial, in dissemination and interpretation of study findings.

Role of the funding source

The funder of the study had no role in study design, data collection, data analysis, data interpretation, or writing the report.

Results

Baseline data were collected across all 12 study clusters from 1392/1584 (87.9%) individuals, of whom 326 individuals were identified with intermediate hyperglycaemia. Endline data were collected from 1343/1584 (84.8%) randomly sampled individuals, and 272/326 (83.4%) of the individuals with intermediate hyperglycaemia at baseline (Figure 1). Sociodemographic characteristics and blood glucose classifications in the baseline and endline random survey samples by study arm are shown in Table 1.

Responders at baseline and endline were more likely to be female (baseline: 92.7% female vs 80.7% male, p<0.001; endline: 89.3% female vs. 78.7% male, p<0.001) than non-responders. Male responders were older than male non-responders (baseline mean difference 4.5 years, p=0.0026; endline mean difference 1.0 year, p=0.1333) and female responders were younger than female non-responders (baseline mean difference=2.4 years, p=0.1464; endline mean difference 2.2 years, p=0.0009). The same patterns of non-response were observed across trial arms.

A total of 108 groups were held by 12 facilitators, with each facilitator responsible for eight to eleven groups per month. Each group had an average of 26 participants and based on our COVID protocol, groups were divided into two separate meetings of around 13 participants in each. Around 60% of the attendees were between 30-40 years old, and the remaining were over 50 years of age. COVID-19 disruptions prevented fidelity to the PLA intervention approach and limited the duration of the intervention. Individual and household actions to prevent diabetes were reported, and group members indicated that they had learned enough information over 10 months to implement individual change. However, PLA group attendance was low after the wider community meeting and data suggest that restricting the number of people attending groups, masking, social distancing, and the discomfort around household visits in a COVID-19 pandemic context did not engender community or group cohesion necessary to trigger mechanisms of community-wide actions. Table 2 shows that 31.6% of the intervention arm sample reported ever participating in a PLA group and those who did participate reported attending a median of 2 out of a maximum of 13 meetings. Approximately 1 in 4 respondents who did not attend PLA groups reported knowing someone who did and 16% of respondents participated in the wider community meeting.

There was no evidence of an intervention effect on the prevalence of intermediate hyperglycaemia and T2DM (adjusted difference, intervention minus control -1.19 (-10.74, 8.36), p=0.7838) (Table 3). Findings were similar in pre-specified sensitivity analyses (Supplementary Table 4).

Amongst our secondary outcomes, large increases in ability to report one or more valid causes, symptoms, complications and strategies for prevention and control of T2DM were observed in the intervention arm relative to control (Table 3). PLA intervention was associated with a significant reduction in diastolic blood pressure (-2.98 (-5.55, -0.41), p=0.0277), but little effect on systolic blood pressure, and possible reductions in the prevalence of hypertension (-7.40 (-16.03, 1.24), p=0.0845) and abdominal obesity (-15.23 (-33.02, 2.56), p=0.0847). Two-year cumulative incidence of T2DM among the intermediate hyperglycaemia cohort was lower in intervention clusters relative to control, but the difference was not statistically significant (-8.60 (-20.09, 2.89), p=0.1265). A potential small negative intervention impact on physical activity is noted.

407 Figure 1: Trial consort profile

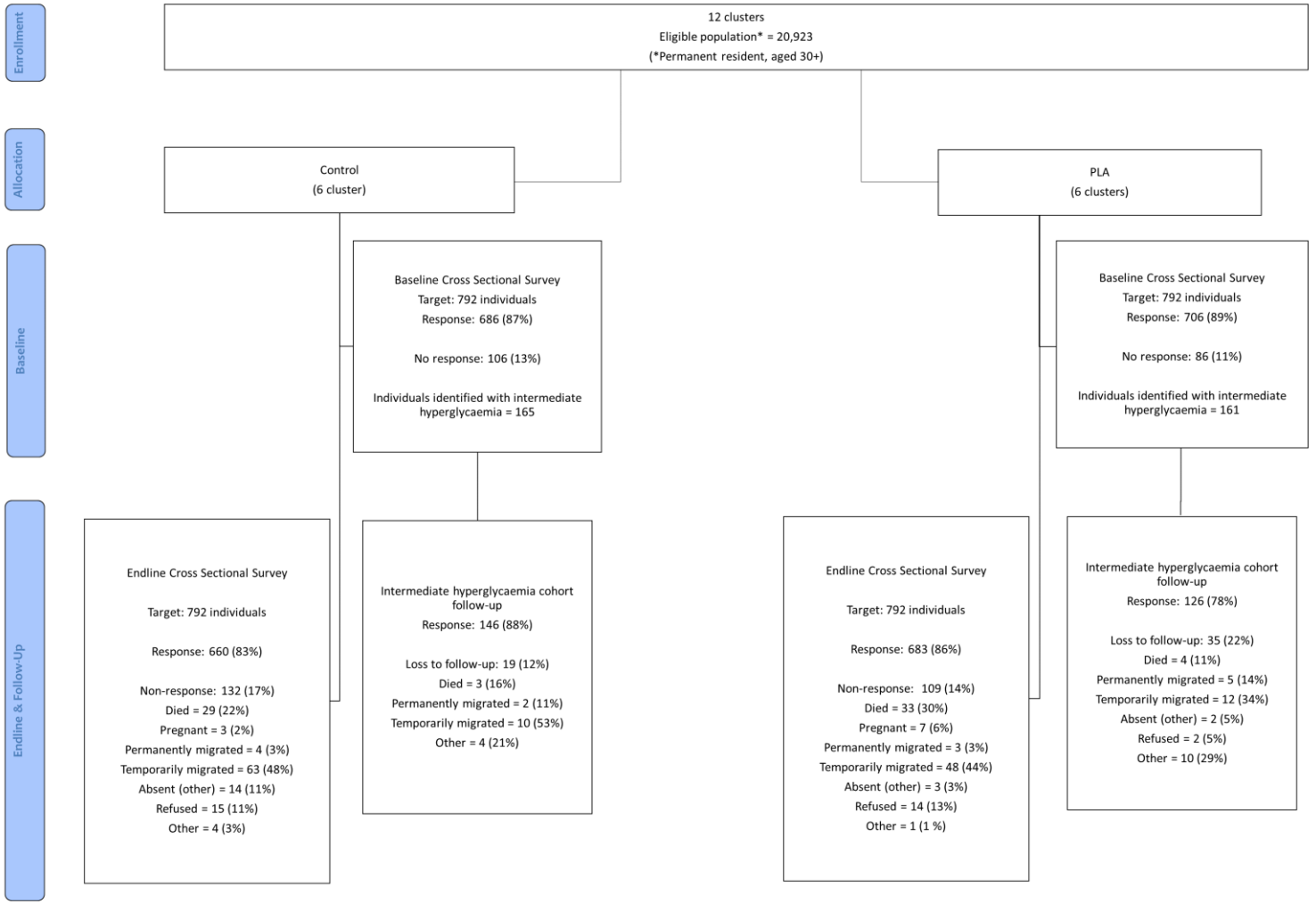

408

409 Table 1: Count and cluster mean sociodemographic characteristics and blood glucose classification by
410 trial arm at baseline and endline random population samples.
411 *IFG=Impaired Fasting Glucose; IGT=Impaired Glucose Tolerance

		Baseline		Endline	
		**Control**	**Intervention**	**Control**	**Intervention**
**Clusters**		6	6	6	6
**Average cluster population aged ≥30 years (sd)**		1750.7 (330.8)	1782.5 (166.7)	1818.7 (342.2)	1840.3 (169.2)
**Average number of households with eligible members per cluster (sd)**		975.7 (76.9)	971.0 (151.8)	990.3 (76.9)	991.4 (153.9)
**Total (survey)**		686	706	660	683
**Age**	**30-39 years**<br>**40-49 years**<br>**50-59 years**<br>**60-69 years**<br>**70-100 years**	198 (28.9%)<br>183 (26.7%)<br>123 (17.9%)<br>118 (17.2%)<br>64 (9.3%)	216 (30.6%)<br>169 (23.9%)<br>121 (17.1%)<br>125 (17.7%)<br>75 (10.6%)	116 (17.6%)<br>196 (29.7%)<br>161 (24.4%)<br>90 (13.6%)<br>97 (14.7%)	129 (18.9%)<br>163 (23.9%)<br>162 (23.7%)<br>119 (17.4%)<br>110 (16.1%)
**Sex**	**Male**<br>**Female**	248 (36.2%)<br>438 (63.9%)	268 (38.0%)<br>438 (62.0%)	243 (36.8%)<br>417 (63.2%)	264 (38.6%)<br>419 (61.4%)
**Education**	**None**<br>**Primary**<br>**Secondary**<br>**Tertiary**	369 (53.8%)<br>231 (33.7%)<br>71 (10.4%)<br>15 (2.2%)	457 (64.7%)<br>192 (27.2%)<br>44 (6.2%)<br>13 (1.8%)	364 (55.2%)<br>235 (35.6%)<br>47 (7.1%)<br>14 (2.1%)	387 (56.7%)<br>231 (33.8%)<br>48 (7.0%)<br>17 (2.5%)
**Illiterate**	**Literate**<br>**Illiterate**	385 (56.1%)<br>301 (43.9%)	354 (50.1%)<br>352 (49.9%)	359 (54.4%)<br>301 (45.6%)	408 (59.7%)<br>275 (40.3%)
**Marital status**	**Married**<br>**Not married**	586 (85.4%)<br>100 (14.6%)	596 (84.4%)<br>110 (15.6%)	554 (83.9%)<br>106 (16.1%)	545 (79.8%)<br>138 (20.2%)
**Religion**	**Muslim**<br>**Other**	660 (96.2%)<br>26 (3.8%)	688 (97.5%)<br>18 (2.6%)	614 (93.0%)<br>46 (7.0%)	670 (98.1%)<br>13 (1.9%)
**Occupation**	**Not working**<br>**Manual labour**<br>**Non-manual labour**	468 (68.2%)<br>183 (26.7%)<br>35 (5.1%)	457 (64.7%)<br>224 (31.7%)<br>25 (3.5%)	448 (67.9%)<br>187 (28.3%)<br>25 (3.8%)	428 (62.7%)<br>229 (33.5%)<br>26 (3.8%)
**Wealth quintile**	**Most poor**<br>**Very poor**<br>**Poor**<br>**Less poor**<br>**Least poor**	109 (15.9%)<br>148 (21.6%)<br>143 (20.9%)<br>167 (24.3%)<br>119 (17.4%)	170 (24.1%)<br>148 (21.0%)<br>118 (16.7%)<br>121 (17.1%)<br>149 (21.1%)	131 (19.9%)<br>145 (22.0%)<br>153 (21.2%)<br>119 (18.0%)<br>112 (17.0%)	141 (20.6%)<br>125 (18.3%)<br>121 (17.7%)<br>142 (20.8%)<br>154 (22.6%)
**Total (diabetes outcomes)**		681	704	660	683
**Diabetes outcomes**	**Normal**<br>**IFG***<br>**IGT***<br>**Diabetes**	374 (54.9%)<br>30 (4.4%)<br>135 (19.8%)<br>142 (20.9%)	435 (61.8%)<br>34 (4.8%)<br>127 (18.0%)<br>108 (15.3%)	331 (50.2%)<br>31 (4.7%)<br>154 (23.3%)<br>144 (21.8%)	372 (54.5%)<br>45 (6.6%)<br>160 (23.4%)<br>106 (15.5%)

412
413 Table 2: PLA intervention coverage process indicators by trial arm.

**Intervention exposure**	**Allocation**	
	**Control**	**Intervention**
**Ever participated in PLA group (%)**	0 (0%)	216 (31.6%)
**Median number of meetings attended (if>0), (IQR; min; max)**	NA	2 (2-5; min.=1, max.=13)
**Respondent knows someone who attended PLA group if respondent did not attend (%)**	0 (0%)	115 (24.6%)
**Participated in community meeting (%)**	1 (0.2%)	109 (16.0%)

414

Table 3 Frequencies and proportions, or means/medians, and absolute (coefficient) effects and 95% confidence interval comparing primary and secondary outcome measures between trial arms (intervention – control)

OUTCOMES		Baseline		Endline		Crude Difference, mean or % (95%CI)*	Adjusted Difference, mean or % (95%CI)**
		Control	Intervention	Control	Intervention		
**PRIMARY**							
**Combined prevalence of intermediate hyperglycaemia and diabetes**		307 (45.1%)	269 (38.2%)	329 (49.9%)	311 (45.5%)	-1.85 (-11.63, 7.92,); p=0.6777	-1.19 (-10.74, 8.36,); p=0.7838
**SECONDARY**							
**Blood pressure**	**Systolic blood pressure (mmHg), mean (sd)**	133.5 (22.6)	133.1 (22.1)	127.0 (20.7)	126.8 (19.1)	0.02 (-3.20, 3.24); p=0.9900	-0.15 (-3.33, 3.04); p=0.9176
	**Diastolic blood pressure (mmHg), mean (sd)**	75.7 (11.4)	79.2 (11.4)	75.8 (11.1)	77.6 (10.8)	-2.78 (-5.24, -0.32,); p=0.0308	-2.98 (-5.55, -0.41); p=0.0277
	**Hypertension, n (%)**	274 (39.9%)	274 (38.8%)	236 (35.8%)	196 (28.7%)	-6.47 (-14.87, 1,93,); p=0.1153	-7.40 (-16.03, 1.24,); p=0.0845
**Overweight & obesity**	**Body Mass Index (BMI), mean (sd)**	23.4 (4.0)	22.7 (3.9)	23.0 (4.0)	22.8 (3.7)	0.38 (-0.30, 1.07); p=0.2404	0.39 (-0.39, 1.17); p=0.2837
	**Overweight or obese, n (%)**	346 (50.4%)	319 (45.3%)	318 (48.2%)	304 (44.5%)	2.59 (-4.84, 10.0), p=0.4504	3.04 (-4.75, 10.83); p=0.4008
	**Waist:Hip ratio, mean (sd)**	0.90 (0.06)	0.89 (0.07)	0.91 (0.05)	0.90 (0.07)	-0.01 (-0.04, 0.02); p=0.4133	-0.01 (-0.04, 0.02); p=0.4420
	**Abdominal obesity, n (%)**	471 (68.7%)	425 (60.2%)	529 (80.2%)	436 (63.8%)	-15.29 (-33.94, 3.36); p=0.0966	-15.23 (-33.02, 2.56); p=0.0847
**Dietary diversity score, mean (SD)**		5.4 (2.2)	5.0 (1.5)	4.7 (1.6)	4.7 (1.4)	-0.10 (-0.59, 0.39); p=0.6566	-0.09 (-0.59, 0.41); p=0.7000
**Log transformed minutes spent engaged in physical activity per week, mean (sd)**		6.4 (0.8)	6.3 (1.0)	6.2 (0.7)	5.9 (0.9)	-0.26 (-0.53, 0.01); p=0.0569	-0.25 (-0.54, 0.04); p=0.0871
**Diabetes knowledge**	**Ability to report one or more valid *causes* of diabetes (%)**	346 (50.4%)	291 (41.2%)	336 (50.9%)	576 (84.3%)	33.73 (17.67, 49.79); p=0.0010	34.07 (18.05, 50.10); p=0.0010
	**Ability to report one or more valid *symptoms* of diabetes (%)**	417 (60.8%)	321 (45.5%)	374 (56.7%)	595 (87.1%)	35.4 (18.21, 52.59); p=0.0012	35.82 (18.67, 52.98); p=0.0011
	**Ability to report one or more valid *complications* of diabetes (%)**	213 (31.1%)	225 (31.9%)	196 (29.7%)	534 (78.2%)	48.17 (28.70, 67.64); p=0.0003	48.66 (29.32, 68.00); p=0.0003
	**Ability to report one or more valid ways to *prevent* diabetes (%)**	407 (59.3%)	385 (54.5%)	451 (68.3%)	601 (88.0%)	21.26 (10.51, 32.01); p=0.0015	21.82 (11.06, 32.58); p=0.0013
	**Ability to report one or more valid ways to *control* diabetes (%)**	453 (66.0%)	410 (58.1%)	498 (75.5%)	606 (88.7%)	14.75 (3.64, 25.86); p=0.0149	15.27 (4.16, 26.39); p=0.0125
**PHQ-9 score ≥10 (moderate or severe depression), n (%)**		51 (7.4%)	33 (4.7%)	27 (4.1%)	24 (3.5%)	-0.12 (-2.93, 2.69); p=0.9232	-0.40 (-3.22, 2.42); p=0.7553
**GAD-7 score ≥10 (moderate or severe anxiety), n (%)**		29 (4.2%)	31 (4.4%)	28 (4.2%)	18 (2.6%)	-1.57 (-4.63, 1.49); p=0.2762	-1.72 (-4.83, 1.38); p=0.2409
**Self-awareness of diabetic status among individuals identified with diabetes by blood glucose measures, n (%)**		70 (49.3%)	47 (43.5%)	69 (47.9%)	51 (48.1%)	0.77 (-20.28, 21.81); p=0.9361	3.61 (-14.89, 22.10); p=0.6694
**Utilisation of services for treatment or advice for diabetes among individuals with prior diagnosis of diabetes**		63 (90.0%)	40 (85.1%)	66 (95.7%)	46 (90.2%)	-4.33 (-15.66, 6.99); p=0.4093	-3.92 (-14.63, 6.79); p=0.4288
**Two-year diabetes incidence among individuals with intermediate hyperglycaemia at baseline, n (%)**				25 (17.1%)	12 (9.5%)	-8.60 (-20.91, 3.72); p=0.1509	-8.60 (-20.09, 2.89); p=0.1265

* Adjustment for baseline outcome measure at the cluster level. **Adjusted for cluster-level baseline outcome measure, gender and age as linear and quadratic terms. Intraclass correlation (ICC) of primary outcome at endline is 0.01.

Table 4: Frequencies, proportions, means/medians and absolute (coefficient) effects and 95% confidence interval comparing explanatory outcome measures by trial arm (intervention – control)

OUTCOMES		Baseline		Endline		Crude Difference (95%CI)*	Adjusted Difference (95%CI)**
		Control	Intervention	Control	Intervention		
**Self-rated health, mean (sd)**		76.1 (17.6)	86.4 (12.8)	69.2 (16.6)	78.0 (18.4)	8.33 (1.91, 14.75); p=0.0167	9.12 (2.58, 15.6); p=0.0116
**Daily smoking or use of smokeless tobacco (%)**		218 (31.8%)	275 (39.0%)	242 (36.7%)	259 (37.9%)	-2.26 (-10.87, 6.35); p=0.5670	-1.44 (-11.1, 8.23); p=0.7439
**Betel nut use (%)**		207 (30.2%)	261 (37.0%)	212 (32.1%)	235 (34.4%)	1.26 (-11.96, 14.5); p=0.8344	0.25 (-12.61, 13.1); p=0.9657
**Log sedentary time in previous 24 hours, median (IQR)**		4.7 (4.4-5.0)	4.7 (4.1-5.2)	4.7 (4.1-5.1)	5.0 (4.8-5.2)	0.26 (0.05, 0.47); p=0.0206	0.24 (0.0, 0.43); p=0.0225
**Log time engaged in brisk walking, median (IQR)**		5.3 (4.8-6.0)	5.1 (4.8-5.6)	5.3 (4.8-5.5)	5.2 (4.8-5.5)	-0.15 (-0.52, 0.22); p=0.3777	-0.13 (-0.49, 0.24); p=0.4454
**Among individuals with prior diagnosis of diabetes**	**Diabetes control (%)**	15 (21.4%)	7 (14.9%)	15 (21.7%)	15 (29.4%)	3.56 (-12.16, 19.28); p=0.6208	1.80 (-15.00, 18.60); p=0.8139
	**Diabetes affect (ADS) score, mean (sd)**	14.9 (3.3)	13.8 (3.2)	16.1 (3.8)	16.5 (3.5)	0.90 (-1.47, 3.26); p=0.4134	0.78 (-1.58, 3.14); p=0.4716
	**Diabetes-related complications (%)**	55 (78.6%)	28 (59.6%)	60 (87.0%)	33 (64.7%)	-22.54 (-48.23, 3.15); p=0.0784	-22.46 (-48.22, 3.30); p=0.0801
	**At least monthly blood glucose testing (%)^**	17 (24.3%)	17 (36.2%)	18 (26.1%)	17 (33.3%)	25.06 (-23.55, 73.68); p=0.2735	19.97 (-29.74, 69.68); p=0.3871
	**Stigma 1: Perceived inability to fulfil responsibilities+**	31 (44.3%)	12 (25.5%)	32 (46.4%)	25 (49.0%)	22.90 (-18.29, 64.09); p=0.2402	23.18, (-17.25, 63.62); p=0.2269
	**Stigma 2: Perceived to be seen as a lesser person+**	18 (25.7%)	4 (8.5%)	13 (18.8%)	14 (27.5%)	18.41 (-35.51, 72.32); p=0.4598	18.08 (-32.23, 68.39); p=0.4373
	**Stigma 3: Embarrassed in social situations+**	29 (41.4%)	5 (10.6%)	20 (29.0%)	17 (33.3%)	38.40 (-8.21, 85.01); p=0.0953	37.17 (-8.32, 82.65); p=0.0976
	**Stigma 4: Ashamed of having diabetes+**	18 (25.7%)	4 (8.5%)	11 (15.9%)	9 (17.7%)	8.79 (-16.79, 34.38); p=0.4567	10.42 (-17.29, 38.13); p=0.4171
	**Feels supported by family in managing diabetes~**	58 (82.9%)	28 (59.6%)	49 (71.0%)	36 (70.6%)	-5.64 (-27.55, 16.27); p=0.5747	-5.05 (-27.15, 17.04); p=0.6173
	**Ever experienced physical, social, or mental abuse due to diabetic status**	27 (38.6%)	5 (10.6%)	20 (29.0%)	17 (33.3%)	7.45 (-31.09, 45.99); p=0.6723	6.81 (-32.38, 46.00); p=0.7035
**Social norms: Perceived negative feelings or family or social reactions to:**	**1) Going on a morning walk alone (women only)**	35 (8.0%)	87 (19.9%)	26 (6.2%)	105 (25.1%)	4.47 (-5.12, 14.06); p=0.3192	4.19 (-5.77, 14.1); p=0.3662
	**2) Going for a morning walk with a female relative (women only)**	10 (2.3%)	26 (5.9%)	15 (3.6%)	74 (17.7%)	7.87 (-2.43, 25.2); p=0.3302	8.12 (-8.90, 25.15); p=0.3084
	**3) Eating less than usual or refusing oily or sugary foods and drinks at social gatherings**	494 (72.0%)	580 (82.2%)	462 (70.0%)	612 (89.6%)	14.36 (2.40, 26.32); p=0.0237	14.59 (2.25, 26.93); p=0.0254
	**4) Providing healthy snacks to guests**	392 (57.1%)	305 (43.2%)	356 (53.9%)	392 (57.4%)	4.02 (-28.58, 36.62); p=0.7866	4.07 (-28.49, 36.63); p=0.7839

* Adjustment for baseline outcome measure at the cluster level. **Adjusted for cluster-level baseline outcome measure, gender and age as linear and quadratic terms. ^Missing data for 9 cases – 1 in control arm at baseline, 4 in each arm at endline. +Missing data for 7 cases – 3 in intervention arm and 1 in control arm at baseline, 2 in intervention arm and 1 in control arm at endline ~Reports at least moderate family support

There was a significant increase in self-rated health in the PLA arm relative to control and a large but non-significant reduction the prevalence of self-reported diabetes-related health complications (Table 4). A small potential negative intervention effect on sedentary time was observed, as was a significant increase in perceived negative social or family reactions to eating less than usual or refusing oily or sugary food and drinks at social gatherings, though wide confidence intervals are noted.

Given the observed intervention effect on diastolic blood pressure, we conducted post-hoc analyses of blood pressure measures. Namely, we evaluated intervention impact on pulse pressure, calculated as the difference between systolic and diastolic blood pressure, and mean arterial pressure, which accounts for flow, resistance and pressure in arteries and is calculated as diastolic blood pressure plus one third of the difference between diastolic and systolic blood pressure. Analysis showed a significant reduction in pulse pressure in intervention clusters relative to control (adjusted difference -1.97 (-3.77, -0.16), p=0.0358). There was also a reduction in mean arterial pressure, though not statistically significant (-1.27 (-3.57, 1.02), p=0.2403). Considering the demonstrated association between anxiety and hypertension in other contexts[29], and in recognition of the hypothesised effects of PLA via mechanisms of enhanced social support[30] we further analysed the GAD-7 measure of anxiety as a log-transformed continuous outcome as opposed to the categorical outcome included in our pre-specified analysis. This showed that there was a significant reduction in transformed GAD-7 score (-0.22 (-0.41, -0.04), p=0.0201) in intervention clusters compared to control arm at endline. Data from post-hoc analyses are presented in Supplementary Table 5.

There was no evidence of interaction between the intervention and gender, household wealth, age, village size, or inclusion in baseline survey in relation to the primary outcome (Supplementary Table 6). Some potential interactions were identified, especially in relation to wealth and outcomes related to diabetes awareness, control, and complications, as well as anxiety and depression. Results suggest intervention effect on incidence of diabetes in the cohort of individuals with intermediate hyperglycaemia at baseline may be modified by wealth index, with poorer individuals benefiting more.

Total and average annual costs of the intervention were US$318,706 and US$157,696, respectively. The total cost per target population (adults $\geq$30 years) covered was US$12. Staff costs constituted 68% of the total intervention cost, followed by other recurrent costs (e.g., overheads) and COVID-19 protection measures, accounting for 13% and 11%, respectively. Costs of the activities conducted in the intervention start-up period (16 months) accounted for around 12% of total costs. Supplementary table 7 provides breakdown of the intervention costs by inputs and activities. ICER was US$4.2 per 1-mmHg reduction in diastolic blood pressure, ranging from $2.2 to $30.3 using the confidence interval around the effect size.

Discussion

We evaluated the effects of facilitated PLA community mobilisation on T2DM and non-communicable disease risk among a general population when horizontally scaled-up and in a dynamic context of COVID-19 in rural Bangladesh. There is no evidence of intervention effect on the primary outcome of prevalence of T2DM and intermediate hyperglycaemia. The intervention is associated with large improvements in diabetes knowledge as well as changes in diastolic blood pressure and self-rated health. Potentially important reductions in the prevalence of hypertension and abdominal obesity, as well as self-reported diabetes-related health complications are also noted, though these do not reach statistical significance. Among a higher-risk population cohort (i.e. individuals with intermediate hyperglycaemia at baseline), the direction and magnitude of intervention effect on incidence of diabetes among is very similar to that observed in our former

DMagic trial (-8.7 (-3.5, -14.0))[6], though given our smaller sample size in D:Clare, the wider confidence intervals included the null.

The absence of a clear intervention effect on the prevalence of intermediate hyperglycaemia and T2DM differs from what we observed in the DMagic trial, which showed large significant effects.[6] However, the intervention also differed in important ways as a result of COVID-19 disruptions and adaptations. Our process evaluation allows us to examine what worked well and, crucially, to analyse fidelity to the PLA intervention that we planned, and factors affecting this.

There were three main ways that the intervention was not implemented as planned because of COVID-19 disruptions and delays: 1. Phases of the intervention were shorter; 2. Group size, average attendance at PLA groups, interaction between group members and facilitators, and between group members was restricted; 3. Community action was not initiated on a large scale. The lack of pre-existing social cohesion in our study context, which was exacerbated by COVID-19 restrictions, has been reported in other studies as a factor which makes it challenging to mobilise communities for social change.[31, 32] The shorter time frame for the first phase of the intervention and interruptions to meeting schedules may also have prevented the development of a core group of members to drive action. A lack of community cohesion in intervention areas fed into a lack of group cohesion, and when combined with restrictions on group size and less consistency in group membership, it was difficult to develop collective responsibility for prevention and control of diabetes. Our intervention was conducive to participatory learning and awareness raising as reflected in diabetes-related awareness outcomes but may not have allowed sufficient time for collective action which usually occurs after the wider community meeting. Compared to DMagic, where 74% of survey respondents in intervention areas reported ever participating in PLA meetings, repeat attendance was high, and 72% knew someone who participated[6], participation in and wider awareness of the intervention was low in D:Clare. Details and results from an in-depth mixed-methods process evaluation of the D:Clare intervention are presented elsewhere.[22]

We have previously described one of the key mechanisms of PLA as a process of enabling individuals to act within enabled, supportive environments.[30, 33] It may be that without adequate time devoted to development of community action and implementation of locally agreed strategies our D:Clare implementation was unable to translate raised awareness into effective collective actions. The findings of increased population awareness without impact on T2DM outcomes is similar to the effect of the mHealth health promotion intervention component of DMagic, which similarly lacked opportunity for collective action.[34] Nevertheless, knowledge and awareness are well-established components of health literacy.[35] This, in turn, supports individuals and communities in preventing and controlling disease, and is necessary to accelerate progress towards global non-communicable disease targets.[35, 36]

We observed a statistically significant reduction in diastolic blood pressure and a suggested reduction in hypertension prevalence in D:Clare, though we note some caution in our certainty of effect due to the number of outcomes tested. Concurrent PLA impact on abdominal obesity and log-transformed anxiety score may have been important mechanisms for this though further exploration of this and the possible role of salt consumption and physical activity changes are needed. While no effects on blood pressure outcomes were observed in the DMagic trial, we did observe similar impacts in our five-year post-randomisation follow-up of DMagic communities.[9] Raised blood pressure and hypertension is the leading modifiable risk factor for future morbidity and mortality from cardiovascular diseases, specially heart attacks and stroke[37, 38] and blood pressure lowering provides broadly generalisable benefits.[39] Diastolic blood pressure, pulse pressure and mean arterial pressure are related to peripheral vascular resistance and loss of arterial vasodilatory mechanisms and have been associated with ischaemic stroke.[40-42]. Data from the Framingham study in the 1990s suggest that even modest reductions in mean population diastolic blood pressure

could result in substantial reductions in the prevalence of hypertension and associated risks of cardiovascular events.[43] The potential to achieve such reductions in blood pressure through population-based interventions of health messaging and community-based strategies such as PLA warrants further exploration, especially in settings that lack screening and treatment for raised blood pressure.

The large, rural, population-based surveys with high response rates at baseline and endline, and the high follow-up of individuals identified with intermediate hyperglycaemia at baseline, with assessment of glycaemic status through fasting and two-hour blood glucose tests are notable strengths of our trial. The inability to ensure blinding of enumerators and participants in the endline survey is a potential weakness. Not all of the same enumerators worked on the endline and baseline surveys and enumerators worked within specific clusters, therefore some inter-observer variation is possible. The use of self-reported measures of behaviours is also a potential weakness of our design and may be subject to recall and social desirability bias and it is plausible that this would vary by trial arm. Measures of physical activity, dietary practice, care-seeking and attitudes must therefore be interpreted with caution. Finally, there was overlap between the timing of baseline data collection and the start of intervention implementation, however, it unlikely that the PLA intervention would have any impacts in this early stage.

The D:Clare intervention cost $12 per target population (adults ≥30 years). This is slightly higher than DMagic intervention, mainly due to longer implementation period (20 vs 18 months) and cost of COVID-19 protection measures. ICER was US$4.2 per 1-mmHg reduction in diastolic blood pressure, ranging from $2.2 to $30.3. ICER for D:Clare is significantly lower than previous community-based hypertension control interventions.[44]

Conclusion

Scale-up of PLA for T2DM prevention and control during the evolving COVID-19 pandemic required adaptations to intervention implementation and evaluation approach. There was no evidence of impact of the adapted intervention on blood glucose outcomes, but large and important impacts on diabetes knowledges and likely effects on other health outcomes, including blood pressure. Process evaluation combined with cluster RCT data provide important insights into intervention and implementation features which are necessary for the success of the intervention. Critical to this is providing sufficient time for intervention delivery that allows communities to develop and share collective knowledge and plan and implement collective action. The PLA approach to non-communicable disease prevention and control continues to show promise.

Contributors

EF, the project PI, led in the design of the study, conducted statistical analyses and drafted the manuscript. AK coordinated project activities in Bangladesh, co-led the project and contributed to intervention development and study design. JM led the process evaluation component of the study and contributed to intervention development. TN developed and coordinated the implementation of PLA group activities. CK provided technical coordination of the surveys and data management processes. SKS coordinated quantitative data collection activities. MP contributed to process evaluation data processing and analysis. SAUA gathered and analysed qualitative process data. JB was responsible for endline survey data processing. NA contributed to intervention development and survey methods. HHB led the economic evaluation component of the project. AC was the trial statistician and contributed to the design, development of the statistical analysis plan and verification of results. AKAK and KA provide technical oversight of all project activities and facilitated community engagement and intervention development activities. All authors contributed to the interpretation of study findings reported here.

Declaration of interests

We declare no competing interests.

Data sharing

Deidentified data collected for this study and a data dictionary are available from the UCL Research Data Repository DOI:10.5522/04/28751210.

Acknowledgements

The study team would like to thank the D:Clare Trial Steering Committee (David Beran (Chair), Graham Hitman, Justine Davies, Edward Gregg, Aaron Holiday, Jennifer Thompson, Calum Davey, Audrey Prost and Anthony Costello) and the Data Monitoring Committee (Mohammod Shahidullah (Chair), Meena Daivadanam, and A.T.M Iqbal Anwar) for their valuable contributions to the project. We are grateful to our Community Advisory Groups and Md. Golam Azam for contributions to field activities.

577 **Supplementary Table 1: PLA group design and adaptations in response to COVID-19**

Design parameter	Original design	Adaptation
Group composition	Open to all community members, with no restrictions on numbers in meetings. DMagic's average group size was 32.	Open to all community members, but meeting size restricted to maximum of 20 participants or less per meeting.
Meeting frequency	Once a month per village	Twice a month per village, to allow for smaller groups but with the same population coverage
Meeting conduct	Participants sit in a circle, and active participation is encouraged.	Participants were asked to wash their hands on arrival/departure, to wear a mask, and try to keep a minimum of 1-meter distance from group members outside of their households. All materials used during the meeting were wiped with alcohol between meetings.
Facilitator role	Facilitators hold 9 meetings per month	Facilitators held 18 meetings per month. Facilitators travelled using well-ventilated transport (e.g. rickshaw) and adhered to infection control practices.
Group communication	Meetings arranged in-person	Phone numbers of group members were collected (after consent) to facilitate communication around meetings, and if needed, to cancel meetings. Numbers were stored locally by facilitators.
Total number of meetings	18 meetings per group delivered over 18 months	Minimum of 13 meetings per group delivered over 20 months. This required more topics to be covered in each meeting.
Community meetings	Large community meetings with several hundred participants held	Smaller stakeholder meetings and use of alternative methods to disseminate group information to wider community, e.g. loudspeaker announcements.

578

579
580

Supplementary Table 2: Glycaemic definitions and diagnostic criteria used in the D:Clare trial, adapted from WHO 2006

**Definition**		**Diagnostic Criteria**
Normoglycaemia		Fasting plasma glucose ≤6.0 mmol/L
Intermediate Hyperglycaemia	Impaired Fasting Glucose (IFG)	Fasting plasma glucose ≥6.1 mmol/L to <7.0 mmol/L **AND** two-hour post ingestion of 75g glucose load plasma glucose <7.8 mmol/L
	Impaired Glucose Tolerance (IGT)	Fasting plasma glucose <7.0 mmol/L **AND** two-hour post ingestion of 75g glucose load plasma glucose ≥7.8 mmol/L to <11.1 mmol/L
Type 2 Diabetes Mellitus (T2DM)		Fasting plasma glucose ≥7.0 mmol/L **OR*** two-hour post ingestion of 75g glucose load plasma glucose ≥11.1 mmol/L **OR** Self-reported prior medical diagnosis of diabetes

**Diabetes cannot be excluded without 2-h post oral glucose load test*

Source: http://apps.who.int/iris/bitstream/10665/43588/1/9241594934_eng.pdf

586 Supplementary Table 3: D:Clare trial secondary outcome measures and definitions

Outcome	Definition	Denominator	Data type
awareness of diabetic status	self-reported diabetes	endline cross-sectional sample with valid endline blood glucose measurements that meet the classification of diabetes and who complete the endline questionnaire	Binary
physical activity	average time spent engaged in physical activity per week	endline cross-sectional sample with valid self-reported physical activity data	Continuous
blood pressure	diastolic and systolic blood pressure	endline cross-sectional sample with valid blood pressure measurements	Continuous
Hypertension	systolic blood pressure ≥ 140 mmHg or a diastolic blood pressure ≥ 90 mmHg or current treatment with antihypertensive medication	endline cross-sectional sample with valid blood pressure measurements	Binary
Body Mass Index	body mass index (weight (kg)/height(m)^2	endline cross-sectional sample with valid weight and height measurements	Continuous
abdominal obesity	waist:hip ratio (WHR)	endline cross-sectional sample with valid waist and hip measurements	Continuous
dietary diversity	Dietary Diversity Score (DDS)	endline cross-sectional sample with valid DDS data	Continuous
knowledge about diabetes symptoms and complications (five separate outcomes)	proportion of adults aged 30 years and above who are able to: a) name at least one cause of diabetes; b) report at least one symptom of diabetes; c) report at least one complication of diabetes; d) report at least one way to reduce the risk of getting diabetes; e) report at least one way to control diabetes if diagnosed.	endline cross-sectional sample of with valid knowledge data	Binary
utilisation of services for treatment or advice for diabetes	proportion of diabetics with self-reported diagnosis of diabetes reporting current or ever receipt of care or advice from a medical professional	endline cross-sectional sample of known (pre-diagnosed) diabetic individuals	Binary
depression	proportion of adults aged 30 years and above with a PHQ score ≥10 (i.e. moderate to severe depressive symptoms).	endline cross-sectional sample of with valid PHQ data.	Binary
anxiety	proportion of adults aged 30 years and above with a GAD-7 score ≥10.	endline cross-sectional sample of with valid GAD-7 data	Binary
18 month cumulative incidence of T2DM among individuals with intermediate hyperglycaemia at baseline	proportion of adults aged 30 years or older with plasma glucose cut-off categorisations for intermediate hyperglycaemia at baseline who are categorised as T2DM at endline	cohort sample of adults aged 30 years or older with plasma glucose cut-off intermediate hyperglycaemia at baseline and followed-up to endline	Binary

587

Supplementary Table 4: Sensitivity analysis of the primary outcome showing frequencies, proportions, estimated difference (intervention minus control) and 95% confidence interval comparing prevalence of intermediate hyperglycaemia and diabetes between trial arms using different outcome definitions.

OUTCOME	Baseline		Endline		Crude Difference (95% CI)*	Adjusted Difference (95% CI)**
	Control	Intervention	Control	Intervention		
**Sensitivity analysis 1: All cases of self-reported diabetes excluded**	237 (38.8%)	222 (33.7%)	260 (44.0%)	260 (41.1%)	0.01 (-10.27, 10.40); p=0.9887	0.18 (-9.86, 10.22); p=0.9682
**Sensitivity analysis 2: All cases defined based on blood glucose measures only (disregarding self-reported diabetes)**	266 (39.1%)	246 (34.8%)	290 (43.9%)	281 (41.1%)	-0.69 (-11.03, 9.67); p=0.8837	-0.37 (-10.56, 9.80); p=0.9349
**Sensitivity analysis 3a^: Arbitrary cut-off definitions of intermediate hyperglycemia and diabetes.**	420 (68.7%)	401 (60.9%)	433 (73.3%)	471 (74.5%)	5.47 (-7.75, 18.69); p=0.3735	5.35 (-7.56, 18.26); p=0.3732
**Sensitivity analysis 3b^^: Arbitrary cut-off definitions of intermediate hyperglycemia and diabetes.**	369 (60.4%)	349 (53.0%)	393 (66.5%)	385 (60.9%)	-1.42 (-14.75, 11.91); p=0.8152	-1.60 (-14.54, 11.34); p=0.7863

* Adjustment for baseline outcome measure at the cluster level.

**Adjusted for cluster-level baseline outcome measure, gender, and age as linear and quadratic terms.

^ IFG fasting glucose 5.5-6.2 mmol/L & 2h glucose <10.4 mmol/L; IGT fasting glucose<6.3 mmol/L & 2h glucose 6.8-10.3 mmol/L; diabetes fasting glucose≥6.3 mmol/L or 2h glucose ≥10.4 mmol/L.

^^ IFG fasting glucose 6.3-7.7 mmol/L & 2h glucose <10.4 mmol/L; IGT fasting glucose<7.8 mmol/L & 2h glucose 6.8-10.3 mmol/L; diabetes fasting glucose≥7.8 mmol/L or 2h glucose ≥10.4 mmol/L.

Supplementary table 5: Post-hoc analysis of intervention effects on pulse pressure, mean arterial pressure and log-transformed anxiety (GAD-7) score.

	Baseline		Endline		Crude Difference (95%CI)*	Adjusted Difference (95%CI)**
	Control	Intervention	Control	Intervention		
**Pulse pressure mmHg, mean (SD)**	57.9 (17.7)	54.0 (16.2)	51.1 (15.0)	49.2 (13.4)	-1.97 (-3.83, -0.12); p=0.0394	-1.97 (-3.77, -0.16); p=0.0358
**Mean arterial pressure, mmHg (SD)**	95.0 (13.7)	97.2 (13.8)	92.9 (13.2)	94.0 (12.6)	-0.83 (-3.21, 1.54); p=0.4473	-1.27 (-3.57, 1.02); p=0.2403
**Log GAD7 score (median, IQR)**	1.36 (1.10-1.95)	1.28 (0.69-1.95)	1.17 (0.69-1.61)	0.93 (0.69-1.39)	-0.24 (-0.43, -0.05); p=0.0181	-0.22 (-0.41, -0.04); p=0.0201

* Adjustment for baseline outcome measure at the cluster level.

**Adjusted for cluster-level baseline outcome measure, gender, and age as linear and quadratic terms.

608 Supplementary table 6: Interaction odds ratios and regression coefficients (95% CIs) between trial arm and gender, wealth index, age,
609 village size and inclusion in baseline sample for primary and secondary study outcomes. Odds ratios (for binary outcomes) and
610 coefficients (for continuous outcomes) indicate the difference in intervention effect on outcomes between interaction categories.

Outcomes		Gender (male compared to female)	Wealth Index (per unit increase in wealth index)	Age (per year increase in age)	Village size (bigger than average compared to smaller than average village)	In baseline survey (yes compared to no)
**Primary outcome – prevalence of DM & intermediate hyperglycaemia⁺**		1.13 (0.63, 2.01)	0.98 (0.83, 1.16)	1.00 (0.98, 1.02)	1.06 (0.54, 2.09)	2.12 (0.64, 7.03)
**Blood pressure**	**Systolic blood pressure^**	0.68 (-6.99, 8.35)	0.18 (-0.93, 1.28)	-0.04 (-0.21, 0.14)	-0.09 (-6.78, 6.60)	0.11 (-5.90, 6.11)
	**Diastolic blood pressure^**	0.15 (-2.78, 3.08)	-0.21 (-1.19, 0.77)	0.06 (-0.04, 0.16)	1.61 (-3.26, 6.48)	1.41 (-2.76, 5.57)
	**Hypertension⁺**	1.19 (0.56, 2.53)	1.12 (0.93, 1.33)	1.00 (0.98, 1.02)	0.86 (0.48, 1.54)	1.22 (0.61, 2.43)
**Overweight & obesity**	**Body Mass Index (BMI)^**	-0.63 (-1.65, 0.38)	-0.18 (-0.46, 0.09)	0.01 (-0.02, 0.05)	0.43 (-1.59, 2.45)	-0.94 (-3.17, 1.29)
	**Overweight or obese⁺**	0.85 (0.54, 1.35)	0.90 (0.74, 1.10)	1.01 (0.99, 1.02)	1.31 (0.59, 2.91)	0.59 (0.19, 1.89)
	**Waist:Hip ratio^**	0.01 (-0.03, 0.04)	0.00 (0.00, 0.01)	0.00 (0.00, 0.00)	0.00 (-0.05, 0.05)	0.00 (-0.03, 0.03)
	**Abdominal obesity⁺**	1.24 (0.40, 3.81)	0.98 (0.84, 1.13)	1.01 (0.98, 1.03)	0.63 (0.15, 2.69)	0.91 (0.27, 3.04)
**Dietary diversity score^**		0.42 (-0.04, 0.88)	-0.02 (-0.14, 0.11)	-0.01 (-0.02, 0.01)	-0.57 (-1.32, 0.18)	-0.20 (-0.64, 0.24)
**Log minutes spent engaged in physical activity per week^**		-0.20 (-0.75, 0.34)	0.04 (-0.05, 0.13)	-0.00 (-0.01, 0.01)	-0.35 (-0.71, 0.02)	0.10 (-0.25, 0.45)
**Diabetes knowledge**	**Ability to report one or more valid *causes* of diabetes⁺**	0.66 (0.31, 1.40)	0.90 (0.69, 1.17)	0.98 (0.96, 1.01)	1.42 (0.32, 6.23)	0.67 (0.23, 1.92)
	**Ability to report one or more valid *symptoms* of diabetes⁺**	1.01 (0.50, 2.04)	0.93 (0.75, 1.16)	0.98 (0.96, 1.00)	2.14 (0.31, 14.74)	0.62 (0.22, 1.76)
	**Ability to report one or more valid *complications* of diabetes⁺**	0.75 (0.34, 1.65)	0.87 (0.64, 1.17)	0.98 (0.96, 1.01)	2.97 (0.43, 20.40)	0.89 (0.34, 2.35)
	**Ability to report one or more valid ways to *prevent* diabetes⁺**	0.84 (0.35, 2.05)	0.96 (0.76, 1.22)	0.98 (0.96, 1.00)	2.71 (0.61, 12.03)	0.69 (0.18, 2.60)
	**Ability to report one or more valid ways to *control* diabetes⁺**	0.95 (0.38, 2.39)	0.89 (0.67, 1.19)	0.99 (0.96, 1.01)	3.28 (0.72, 15.06)	0.80 (0.18, 3.61)
**PHQ-9 score ≥10 (moderate or severe depression)⁺**		2.24 (0.57, 8.84)	1.83 (1.26, 2.66)**	1.00 (0.93, 1.07)	0.27 (0.06, 1.26)	2.35 (0.29, 19.00)
**GAD-7 score ≥10 (moderate or severe anxiety)⁺**		1.09 (0.16, 7.37)	1.85 (1.28, 2.68)**	1.00 (0.93, 1.07)	0.50 (0.07, 3.40)	4.65 (0.39, 55.30)
**Self-awareness of status among individuals identified with DM by blood glucose measures⁺**		0.36 (0.08, 1.60)	1.32 (0.98, 1.77)	1.02 (0.98, 1.05)	1.34 (0.32, 5.70)	1.24 (0.30, 5.06)
**Use of services for treatment/advice for diabetes among individuals with prior diagnosis⁺**		No data	0.22 (0.10, 0.46)***	1.19 (1.11, 1.28)***	No data	No data
**Two-year diabetes incidence among individuals with intermediate hyperglycaemia⁺**		0.57 (0.07, 4.92)	1.77 (1.12, 2.80)*	1.03 (0.95, 1.11)	0.78 (0.12, 5.00)	NA
**Self-rated health^**		-3.52 (-8.45, 1.41)	-2.19 (-3.93, -0.47)*	0.12 (-0.05, 0.29)	-1.44 (-8.12, 5.24)	-3.21 (-9.97, 3.56)
**Daily smoking or use of smokeless tobacco⁺**		-0.94 (-6.07, 4.19)	-1.10 (-2.69, 0.49)	-0.05 (-0.17, 0.06)	-2.40 (-5.67, 0.87)	0.71 (-3.12, 4.54)
**Betel nut use⁺**		1.31 (0.67, 2.57)	1.07 (0.93, 1.23)	1.00 (0.98, 1.02)	1.51 (0.80, 2.88)	0.82 (0.32, 2.12)
**Log sedentary time in previous 24 hours^**		0.32 (0.17, 0.46)***	-0.03 (-0.08, 0.02)	-0.00 (-0.01, 0.00)	-0.29 (-0.60, 0.02)	0.24 (-0.03, 0.50)

**Log time engaged in brisk walking^**	0.20 (-0.26, 0.66)	-0.01 (-0.10, 0.07)	0.00 (-0.01, 0.01)	-0.06 (-0.37, 0.25)	-0.27 (-0.51, -0.02)*
**Among people living with diabetes**					
**Diabetes control+**	1.21 (0.12, 12.58)	2.18 (1.17, 4.07)*	1.00 (0.91, 1.09)	0.87 (0.18, 4.28)	0.71 (0.01, 52.92)
**Diabetes affect (ADS) score**	-0.94 (-6.07, 4.19)	-1.10 (-2.69, 0.49)	-0.05 (-0.17, 0.06)	-2.40 (-5.67, 0.87)	0.71 (-3.12, 4.54)
**Diabetes-related complications+**	No data	0.36 (0.20, 0.65)**	0.97 (0.89, 1.06)	1.73 (0.07, 43.93)	5.74 (0.19, 169.45)
**At least monthly blood glucose testing+**	0.88 (0.09, 8.22)	0.66 (0.30, 1.44)	0.96 (0.88, 1.04)	0.68 (0.02, 21.29)	No data
**Stigma 1: Perceived inability to fulfil responsibilities+**	3.57 (0.64, 19.86)	0.91 (0.45, 1.81)	0.96 (0.90, 1.01)	1.73 (0.03, 110.44)	0.11 (0.01, 1.17)
**Stigma 2: Perceived to be seen as a lesser person+**	1.84 (0.22, 15.51)	0.37 (0.22, 0.62)**	0.96 (0.88, 1.04)	57.10 (3.18, 1023.88)*	0.06 (0.00, 1.11)
**Stigma 3: Embarrassed in social situations+**	3.53 (0.49, 25.53)	0.52 (0.20, 1.35)	0.93 (0.90, 0.97)**	20.01 (1.67, 239.86)*	No data
**Stigma 4: Ashamed of having diabetes+**	No data	1.22 (0.76, 1.94)	1.01 (0.98, 1.05)	7.78 (0.54, 112.61)	No data
**Feels supported by family in managing diabetes+**	1.96 (0.06, 59.42)	0.83 (0.50, 1.38)	0.96 (0.88, 1.05)	0.38 (0.05, 2.98)	No data
**Ever experienced physical, social, or mental abuse due to diabetic status+**	0.37 (0.04, 3.93)	0.83 (0.61, 1.11)	0.94 (0.88, 1.00)*	0.85 (0.11, 6.69)	No data
**Social norms**					
**1) Going on a morning walk alone (women only)+**	NA	1.03 (0.64, 1.65)	0.95 (0.91, 0.98)**	3.17 (0.17, 59.87)	1.62 (0.34, 7.76)
**2) Going for a morning walk with a female relative (women only)+**	NA	0.94 (0.62, 1.42)	0.95 (0.90, 1.01)	2.16 (0.08, 59.38)	No data
**3) Eating less than usual or refusing oily or sugary foods and drinks at social gatherings+**	1.02 (0.31, 3.33)	0.98 (0.76, 1.28)	1.00 (0.97, 1.04)	0.80 (0.15, 4.40)	1.16 (0.58, 2.30)
**4) Providing healthy snacks to guests+**	2.73 (1.35, 5.52)**	0.93 (0.75, 1.15)	0.99 (0.97, 1.01)	0.95 (0.10, 9.48)	1.48 (0.64, 3.43)

611 * p<0.05; **p<0.01; ***p<0.001

612 ^Continuous outcome – results show regression coefficient and 95% confidence intervals of interaction term (intervention effect on outcome by column interaction parameter)

613 +Binary outcome – results show odds ratio and 95% confidence intervals of interaction term (intervention effect on outcome by column interaction parameter)

614

615

616

Supplementary table 7: Intervention costs disaggregated by input, activities and implementation phase

Description	US$	%
**Inputs/Line items**		
Staff	215,349	68%
Materials for running PLA	2,326	1%
Other materials	-	0%
Materials-COVID protocol	35,671	11%
Other recurrent	41,372	13%
Travel/Transportation	23,062	7%
Capital	925	0%
**Activities**		
Adapting PLA materials	23,160	2%
Community sensitisation	39,865	4%
PLA-training	18,034	2%
Covid-19 protocols	95,862	10%
Other start up activities	813	0%
PLA implementation	448,688	48%
Coordination	13,569	1%
supervision	6,756	1%
General M&E	2,961	0%
Admin & joint activities	282,434	30%
**Implementation phase**		
Start up	55,880	18%
Implementation	262,826	82%
**Total costs**	**318,706**	**100%**

References

1. Cousin E, Duncan BB, Stein C, Ong KL, Vos T, Abbafati C, et al. Diabetes mortality and trends before 25 years of age: an analysis of the Global Burden of Disease Study 2019. The Lancet Diabetes & Endocrinology. doi: 10.1016/S2213-8587(21)00349-1.
2. Lin X, Xu Y, Pan X, Xu J, Ding Y, Sun X, et al. Global, regional, and national burden and trend of diabetes in 195 countries and territories: an analysis from 1990 to 2025. Scientific Reports. 2020;10(1):14790. doi: 10.1038/s41598-020-71908-9.
3. International Diabetes Federation. IDF Diabetes Atlas. 10th ed. Brussels, Belgium: International Diabetes Federation; 2021.
4. Manne-Goehler J, Geldsetzer P, Agoudavi K, Andall-Brereton G, Aryal KK, Bicaba BW, et al. Health system performance for people with diabetes in 28 low- and middle-income countries: A cross-sectional study of nationally representative surveys. PLoS Med. 2019;16(3):e1002751. Epub 20190301. doi: 10.1371/journal.pmed.1002751. PubMed PMID: 30822339; PubMed Central PMCID: PMCPMC6396901.
5. Akhtar S, Nasir JA, Sarwar A, Nasr N, Javed A, Majeed R, et al. Prevalence of diabetes and pre-diabetes in Bangladesh: a systematic review and meta-analysis. BMJ Open. 2020;10(9):e036086. doi: 10.1136/bmjopen-2019-036086.
6. Fottrell E, Ahmed N, Morrison J, Kuddus A, Shaha SK, King C, et al. Community groups or mobile phone messaging to prevent and control type 2 diabetes and intermediate hyperglycaemia in Bangladesh (DMagic): a cluster-randomised controlled trial. The Lancet Diabetes & Endocrinology. 2019;7(3):200-12. doi: https://doi.org/10.1016/S2213-8587(19)30001-4.
7. Morrison J, Akter K, Jennings HM, Kuddus A, Nahar T, King C, et al. Implementation and fidelity of a participatory learning and action cycle intervention to prevent and control type 2 diabetes in rural Bangladesh. Global Health Research and Policy. 2019;4(1):19. doi: 10.1186/s41256-019-0110-6.
8. Pires M, Shaha S, King C, Morrison J, Nahar T, Ahmed N, et al. Equity impact of participatory learning and action community mobilisation and mHealth interventions to prevent and control type 2 diabetes and intermediate hyperglycaemia in rural Bangladesh: analysis of a cluster randomised controlled trial. J Epidemiol Community Health. 2022;76(6):586-94.
9. Fottrell E, King C, Ahmed N, Shaha SK, Morrison J, Pires M, et al. Intermediate hyperglycaemia, diabetes and blood pressure in rural Bangladesh: five-year post-randomisation follow-up of the DMagic cluster-randomised controlled trial. The Lancet Regional Health - Southeast Asia. 2023;10. doi: 10.1016/j.lansea.2022.100122.
10. King C, Pires M, Ahmed N, Akter K, Kuddus A, Copas A, et al. Community participatory learning and action cycle groups to reduce type 2 diabetes in Bangladesh (D:Clare): an updated study protocol for a parallel arm cluster randomised controlled trial. Trials. 2023;24(1):218. doi: 10.1186/s13063-023-07243-x.
11. King C, Pires M, Ahmed N, Akter K, Kuddus A, Copas A, et al. Community participatory learning and action cycle groups to reduce type 2 diabetes in Bangladesh (D:Clare trial): study protocol for a stepped-wedge cluster randomised controlled trial. Trials. 2021;22(1):235. doi: 10.1186/s13063-021-05167-y.
12. Research NIoP, Training - NIPORT/Bangladesh, Mitra, Associates, ICF International. Bangladesh Demographic and Health Survey 2014. Dhaka, Bangladesh: NIPORT, Mitra and Associates, and ICF International, 2016.
13. Morrison J, Hossin R, Kuddus A, Pires M, King C, Ahmed N, et al. Community engagement in population-level research in the context of Covid-19 in rural Bangladesh. Research for All. 2023;7(1). doi: 10.14324/rfa.07.1.17. PubMed PMID: ScienceOpenVid:0a82dac7-e46d-41b4-91bd-4bd7ed1002ac.
14. Rifkin SB, Pridmore P, Cost FfTAaL. Partners in Planning: Information, Participation and Empowerment: Macmillan Education Limited; 2001.

15. Bonita R, de Courten M, Dwyer T, Jamrozik K, R W. Surveillance of risk factors for noncommunicable diseases: The WHO STEPwise approach. Summary. Geneva: World Health Organization,, 2001.
16. Mendenhall E, Norris SA, Shidhaye R, Prabhakaran D. Depression and type 2 diabetes in low- and middle-income countries: A systematic review. Diabetes Research and Clinical Practice. 2014;103(2):276-85. doi: https://doi.org/10.1016/j.diabres.2014.01.001.
17. Spitzer RL, Kroenke K, Williams JBW, Löwe B. A Brief Measure for Assessing Generalized Anxiety Disorder: The GAD-7. Archives of Internal Medicine. 2006;166(10):1092-7. doi: 10.1001/archinte.166.10.1092.
18. Kroenke K, R. S. The PHQ-9: A New Depression Diagnostic and Severity Measure. Psychiatr Ann. 2002;32:509-15. doi: doi: 10.3928/0048-5713-20020901-06.
19. Redwan ASM, Karim MR, Royle RKS, Chowdhury AR. Validation of Bangla generalized anxiety disorder 7 scale among general population. Bangladesh Journal of Psychiatry. 2020;34(1):3-8. doi: 10.3329/bjpsy.v34i1.71969.
20. Pardhan S, Siddique AB, Motahara U, Islam MS. Investigating the prevalence and associated factors of depression, anxiety, and loneliness among people with type-2 diabetes in Bangladesh: a community-based study. Sci Rep. 2024;14(1):25129. Epub 20241024. doi: 10.1038/s41598-024-75144-3. PubMed PMID: 39448658; PubMed Central PMCID: PMCPMC11502699.
21. Lohman TG, Roche AF, Martorell R. Anthropometric standardization reference manual. 1988.
22. Morrison J, Pires M, Ahmed SAU, King C, Jeny TJ, Hossin R, et al. How did the context of COVID-19 affect the implementation and mechanisms of participatory learning and action to address type 2 diabetes? Mixed-methods research in rural Bangladesh. BMJ Open. 2025;15(4):e089288. doi: 10.1136/bmjopen-2024-089288.
23. Moore GF, Audrey S, Barker M, Bond L, Bonell C, Hardeman W, et al. Process evaluation of complex interventions: Medical Research Council guidance. BMJ : British Medical Journal. 2015;350:h1258. doi: 10.1136/bmj.h1258.
24. Gale NK, Heath G, Cameron E, Rashid S, Redwood S. Using the framework method for the analysis of qualitative data in multi-disciplinary health research. BMC Medical Research Methodology. 2013;13(1):117. doi: 10.1186/1471-2288-13-117.
25. Carey MP, Jorgensen RS, Weinstock RS, Sprafkin RP, Lantinga LJ, Carnrike CLM, et al. Reliability and validity of the Appraisal of Diabetes Scale. Journal of Behavioral Medicine. 1991;14(1):43-50. doi: 10.1007/bf00844767.
26. Copas AJ, Hooper R. Cluster randomised trials with different numbers of measurements at baseline and endline: Sample size and optimal allocation. Clin Trials. 2020;17(1):69-76. Epub 20191003. doi: 10.1177/1740774519873888. PubMed PMID: 31580144; PubMed Central PMCID: PMCPMC7334046.
27. Hayes RJ, Moulton LH. Cluster Randomised Trials. 2nd ed. Boca Raton, FL, USA: Chapman & Hall/CRC; 2017.
28. World Bank Group. PPP conversion factor, GDP (LCU per international $) - Bangladesh [19th July 2023]. Available from: https://data.worldbank.org/indicator/PA.NUS.FCRF?locations=BD.
29. Pan Y, Cai W, Cheng Q, Dong W, An T, Yan J. Association between anxiety and hypertension: a systematic review and meta-analysis of epidemiological studies. Neuropsychiatr Dis Treat. 2015;11:1121-30. Epub 20150422. doi: 10.2147/ndt.S77710. PubMed PMID: 25960656; PubMed Central PMCID: PMCPMC4411016.
30. Morrison J, Akter K, Jennings H, Nahar T, Kuddus A, Shaha S, et al. Participatory Learning and Action to address type 2 diabetes in rural Bangladesh: a qualitative process evaluation. BMC Endocrine Disorders. 2019;19(118). doi: doi.org/10.1186/s12902-019-0447-3.
31. Nic a Bháird C. The Complexity of Community Engagement: Developing Staff–Community Relationships in a Participatory Child Education and Women's Rights Intervention in Kolkata Slums. Journal of Community & Applied Social Psychology. 2013;23(5):389-404. doi: https://doi.org/10.1002/casp.2133.

731 32. Gaikwad SS, Bhende A, Nidhi G, Saggurti N, Ranebennur V. How effective is community
732 mobilisation in HIV prevention among highly diverse sex workers in urban settings? The Aastha
733 intervention experience in Mumbai and Thane districts, India. J Epidemiol Community Health.
734 2012;66 Suppl 2:ii69-77. Epub 20120703. doi: 10.1136/jech-2011-200514. PubMed PMID: 22760223.
735 33. Morrison J, Akter K, Jennings H, Kuddus A, Nahar T, King C, et al. Implementation and fidelity
736 of a participatory learning and action cycle intervention to prevent and control type 2 diabetes in
737 rural Bangladesh. Global Health Research and Policy. 2019;4(19).
738 34. Morrison J, Akter K, Jennings H, Ahmed N, Kumer Shaha S, Kuddus A, et al. Learning from a
739 diabetes mHealth intervention in rural Bangladesh: what worked, what did not and what next?
740 Global Public Health. 2022;17(7):1299-313. doi: 10.1080/17441692.2021.1923776.
741 35. Heine M, Lategan F, Erasmus M, Lombaard C-M, Mc Carthy N, Olivier J, et al. Health
742 education interventions to promote health literacy in adults with selected non-communicable
743 diseases living in low-to-middle income countries: A systematic review and meta-analysis. Journal of
744 Evaluation in Clinical Practice. 2021;27(6):1417-28. doi: https://doi.org/10.1111/jep.13554.
745 36. World Health Organization. Health literacy development for the prevention and control of
746 noncommunicable diseases. Geneva: World Health Organization, 2022.
747 37. Yusuf S, Joseph P, Rangarajan S, Islam S, Mente A, Hystad P, et al. Modifiable risk factors,
748 cardiovascular disease, and mortality in 155 722 individuals from 21 high-income, middle-income,
749 and low-income countries (PURE): a prospective cohort study. Lancet. 2020;395(10226):795-808.
750 Epub 20190903. doi: 10.1016/s0140-6736(19)32008-2. PubMed PMID: 31492503; PubMed Central
751 PMCID: PMCPMC8006904.
752 38. Ezzati M, Riboli E. Behavioral and dietary risk factors for noncommunicable diseases. N Engl J
753 Med. 2013;369(10):954-64. doi: 10.1056/NEJMra1203528. PubMed PMID: 24004122.
754 39. Ettehad D, Emdin CA, Kiran A, Anderson SG, Callender T, Emberson J, et al. Blood pressure
755 lowering for prevention of cardiovascular disease and death: a systematic review and meta-analysis.
756 The Lancet. 2016;387(10022):957-67. doi: https://doi.org/10.1016/S0140-6736(15)01225-8.
757 40. Giles TD, Sander GE. Diastolic Blood Pressure and Hypertension Phenotypes: The US Food
758 and Drug Administration Has It Right. The Journal of Clinical Hypertension. 2013;15(3):145-6. doi:
759 https://doi.org/10.1111/jch.12053.
760 41. Kandil H, Soliman A, Alghamdi NS, Jennings JR, El-Baz A. Using Mean Arterial Pressure in
761 Hypertension Diagnosis versus Using Either Systolic or Diastolic Blood Pressure Measurements.
762 Biomedicines. 2023;11(3). Epub 20230310. doi: 10.3390/biomedicines11030849. PubMed PMID:
763 36979828; PubMed Central PMCID: PMCPMC10046034.
764 42. Zheng L, Sun Z, Li J, Zhang R, Zhang X, Liu S, et al. Pulse Pressure and Mean Arterial Pressure
765 in Relation to Ischemic Stroke Among Patients With Uncontrolled Hypertension in Rural Areas of
766 China. Stroke. 2008;39(7):1932-7. doi: doi:10.1161/STROKEAHA.107.510677.
767 43. McEvoy JW, Daya N, Rahman F, Hoogeveen RC, Blumenthal RS, Shah AM, et al. Association
768 of Isolated Diastolic Hypertension as Defined by the 2017 ACC/AHA Blood Pressure Guideline With
769 Incident Cardiovascular Outcomes. JAMA. 2020;323(4):329-38. doi: 10.1001/jama.2019.21402.
770 44. Zhang D, Wang G, Joo H. A Systematic Review of Economic Evidence on Community
771 Hypertension Interventions. Am J Prev Med. 2017;53(6s2):S121-s30. doi:
772 10.1016/j.amepre.2017.05.008. PubMed PMID: 29153113; PubMed Central PMCID:
773 PMCPMC5819001.

774

CONSORT checklist

Click here to access/download
Supporting Information
20240612_CONSORTChecklist.docx

Original published protocol

Click here to access/download
Supporting Information
DClare_Protocol1.pdf

Updated published protocol

Click here to access/download
Supporting Information
DClare_Protocol2.pdf

Statistical Analysis Plan - signed by TSC and DMC

Click here to access/download
Supporting Information
DClare_StatisticalAnalysisPlan.pdf

Ethics extension approval

Click here to access/download
Supporting Information
Ethics_Extension_Approved.pdf

Click here to access/download
Supporting Information
20250408_Inclusivity-in-global-research-questionnaire.docx

A cluster randomised controlled trial of community groups using Participatory Learning and Action to prevent and control diabetes and intermediate hyperglycaemia in rural Bangladesh.

Short Title: The DClare trial of PLA to prevent and control diabetes in Bangladesh.

Edward Fottrell PhD[1*] (0000-0003-0518-7161), Abdul Kuddus MPH[2], Joanna Morrison PhD[1], Tasmin Nahar MSS[2], Carina King PhD[3], Sanjit Kumer Shaha MSS[2], Malini Pires MPH[1], Sarker Ashraf Uddin Ahmed MPH[2], James Beard BSc[4], Naveed Ahmed MPH[2], Andrew Copas PhD[1], Hassan Haghparast-Bidgoli PhD[1], A.K. Azad Khan D.Phil[2], Kishwar Azad FCPS[2]

1 University College London Institute for Global Health, London, United Kingdom
2 Centre for Health Research and Implementation, Diabetic Association of Bangladesh, Dhaka, Bangladesh
3 Department of Global Public Health, Karolinska Institutet, Stockholm, Sweden
4 Independent Consultant, Guildford, United Kingdom

*Correspondence to: Edward Fottrell e.fottrell@ucl.ac.uk

Author contributions: EF was Principal Investigator, wrote the first draft, conducted analyses and led the design of the study. AK oversaw the implementation of implementation and evaluation activities; JM led the process evaluation and drafted relevant parts of the manuscript; TN was responsible for intervention implementation; CK contributed to study design and led in data processing; SKS led evaluation and survey activities; MP contributed to study design and process evaluation; SAUA implemented process evaluation ~~and implementation~~ research activities; JB contributed to data processing; NA contributed to intervention and survey design; AC was the trial statistician; HHB led the economic evaluation; AKAK and KA facilitated project activities and contributed to project design and implementation. All authors contributed to interpretation of the data and revisions of the manuscript.

Abstract

Objectives

To evaluate the impact of Participatory Learning and Action (PLA) community mobilisation intervention for diabetes prevention and control when implemented in a new area in rural Bangladesh and in the context of COVID-19.

Design

Cluster randomised controlled trial.

Setting

Rural communities in Alfadanga Upazila, Faridpur District, Bangladesh.

Participants

Adults aged ≥30 years who permanently reside in study villages. A random cross-sectional sample of 1392 adults at baseline (pre-intervention) and 1343 at endline (post-intervention) was selected to evaluate intervention impact.

Intervention

Community mobilisation through PLA, which works through facilitated community groups actively engaging communities in identifying the causes of health problems~~,~~ and working together to design and implement ways to address these health problems, and reflect on their progress. ~~108~~One hundred and eight PLA groups were created with the intention to run for 18 months, with groups meeting once a month. Due to COVID-related interruptions, however, implementation was modified to allow a minimum of 13 meetings held over a total implementation period of 20 months.

Main outcome measures

Prevalence of intermediate hyperglycaemia and diabetes assessed through fasting blood glucose concentrations and two-hour oral glucose tolerance tests. Secondary outcomes included blood pressure, knowledge of diabetes and behavioural outcomes, including diet, physical activity, and care-seeking.

Results

There was no evidence of an intervention effect on the primary outcome of prevalence of intermediate hyperglycaemia and diabetes between study arms (adjusted difference -1.19% (95% CI -10.74, 8.36), p=0.784). Large increases in diabetes knowledge were recorded and the intervention was associated with a significant reduction in mean diastolic blood pressure (-2.98 (-5.55, -0.41), p=0.028) and possible reductions in the prevalence of hypertension (-7.40% (-16.03, 1.24), p=0.0845) and abdominal obesity (-15.23% (-33.02, 2.56), p=0.085).

Conclusion

After a total of 20 months of intervention delivery there was no evidence of impact of PLA on blood glucose outcomes, but large impacts on diabetes knowledges were seen and likely impacts on some health outcomes, including mean diastolic blood pressure. Interruptions to implementation of PLA for diabetes prevention and control during the evolving COVID-19 pandemic required a reduction in the total number of group meetings and other adaptations that may have affected intervention effectiveness. Nevertheless, the PLA approach to non-communicable disease prevention and control continues to show promise in resource-poor settings.

Funding

This work was funded by the Medical Research Council UK (MR/T023562/1) under the Global Alliance for Chronic Diseases Scale-Up Programme.

Trial registration: ISRCTN42219712 – registered 31st October 2019. Status: complete.

Introduction

~~Between 2015 and 2018, the DMagic (Diabetes Mellitus Action through community Groups or mHealth Information for better Control) trial used a participatory learning and action (PLA) approach for type-2 diabetes mellitus (T2DM) prevention and control directed at the general population in rural Bangladesh.[1] After 18 months of implementation of 122 monthly PLA meetings, the trial showed significant increases in diabetes knowledge and awareness and large reductions in the prevalence of diabetes and intermediate hyperglycaemia, with an absolute reduction of -20.1% (95% CI: 26.1, -14.0).[1, 2] Among individuals identified with intermediate hyperglycaemia, the two-year cumulative incidence odds of diabetes was 59% lower (OR: 0.41; 95% CI:0.24, 0.67), equating to an absolute reduction of -8.4% (95% CI: -13.8, -2.99).[1] Impact was shown to be highly cost effective, equitable and,[3] though diabetes outcomes were no longer observed at 5-years post randomisation, other longer-term potential health impacts were notable, including reductions in population hypertension outcomes.[4]~~

Globally, an estimated 537 million adults aged 20-79 have diabetes and more than three quarters of these people live in low- and middle-income countries.[1-3] By 2045, the International Diabetes Federation predicts that 1 in 8 adults in this age range – approximately 783 million – will be living with diabetes, an increase of 46% and with the largest increases occurring in resource-poor settings.[3] The majority of people living with diabetes in low- and middle-income settings are undiagnosed and unaware of their condition, and health systems in resource-poor settings are ill-equipped to respond to the growing burden of diabetes risk and morbidity.[4] There is therefore a pressing need for effective, scalable and sustainable population-level initiatives to raise awareness and to prevent and control diabetes globally, and particularly in resource-poor settings.

In Bangladesh, the prevalence of diabetes is estimated to range from 2% to 13% and the estimated prevalence of intermediate hyperglycaemia (impaired fasting glucose or impaired glucose tolerance indicating an increased risk of diabetes) varies between 2% and 22%.[5] Between 2015 and 2018, the DMagic (Diabetes Mellitus Action through community Groups or mHealth Information for better Control) trial used a participatory learning and action (PLA) approach for type-2 diabetes mellitus (T2DM) prevention and control directed at the general population in rural Bangladesh.[6] After 18 months of implementation of 122 monthly PLA meetings, the trial showed significant increases in diabetes knowledge and awareness and large reductions in the prevalence of diabetes and intermediate hyperglycaemia, with an absolute reduction of -20.1% (95% CI:-26.1, -14.0).[6, 7] Among individuals identified with intermediate hyperglycaemia, the two-year cumulative incidence odds of diabetes was 59% lower (OR: 0.41; 95% CI:0.24, 0.67), equating to an absolute reduction of -8.4% (95% CI: -13.8, -2.99).[6] Impact was shown to be highly cost effective, equitable and,[8] though diabetes outcomes were no longer observed at 5-years post randomisation, other longer-term potential health impacts were notable, including reductions in population hypertension outcomes.[9]

Given the need for context-appropriate interventions to prevent diabetes at scale, the Diabetes: Community-led Awareness, Response and Evaluation (D:Clare) trial assesses the impact of PLA on T2DM and other non-communicable disease and risk outcomes when implemented across a sub-district ('upazila') in Bangladesh and the context of COVID-19, with pragmatic adaptations to intervention delivery and evaluation.[~~5, 6~~10, 11]

Methods

Setting

The D:Clare trial took place between January 2020 and December 2022 in Alfadanga upazila, Faridpur district, Bangladesh. Alfadanga was purposefully selected as the trial location as it had not been exposed to a PLA intervention previously and is close to an existing field office. Alfadanga has an estimated population of 120,000 people, divided into six administrative unions and has a predominantly agricultural economy.[~~7~~12]

Field Code Changed

Study design and participants

Originally designed as a stepped-wedge cluster randomised controlled trial~~[6]~~[11] we changed our design to a parallel-arm two group cluster RCT with 1:1 allocation~~[5]~~[10] due to COVID-19-related project interruptions and potential impacts on health and contextual factors.~~[8]~~[13]

Each of the six *unions* in Alfadanga were split in two, forming 12 clusters (population approx.=10,000 each). Evaluation was based on data collected at baseline (post randomisation but pre-intervention delivery) and endline (post intervention) cross-sectional surveys. To minimise inter-cluster contamination, we employed a 'fried-egg' design for our evaluation surveys, with participants residing in border areas excluded from surveys. Everyone in intervention clusters was eligible to participate in the intervention but evaluation was restricted to non-pregnant female and male permanent residents aged 30 years and above.

Ethics

The trial received ethical approval from University College London (4199/007) and the Diabetic Association of Bangladesh (BADAS-ERC/E/19/00276). All survey participants indicated informed consent through signature or thumbprint.

Randomisation and masking

Randomisation took place at a project orientation meeting in Faridpur town attended by community representatives and independent observers. ~~12~~Twelve folded pieces of paper, each with a name one of the clusters, were placed into a container and then drawn by meeting participants. The first six clusters were allocated to intervention, and the subsequent six were allocated to control.

Intervention

Participatory Learning and Action

The intervention was community mobilisation through PLA, which works through facilitated community groups actively engaging communities in identifying the causes of health problems, and working together to design and implement ways to address these health problems, and reflect on their progress.[~~9~~14]

Field Code Changed

~~108~~One hundred and eight PLA groups, 9 men's and 9 women's in each intervention cluster - approximately 1 per 200 adults aged 30 years and above – were created with the intention to work through four phases of PLA: 1) problem identification where participants identify and prioritise causes of T2DM and T2DM risk in their community; 2) planning together where groups and communities collectively design strategies to address the causes of T2DM that can be implemented by communities; 3) strategy implementation; 4) participatory evaluation of the strategies which they have implemented. The four-phase cycle was planned to take 18 months, with groups meeting once a month. Due to COVID-related interruptions, however, implementation was modified to allow a minimum of 13 meetings held over a total implementation period of 20 months. Additional COVID-related modifications to the intervention delivery are summarised in Supplementary Table 1. Those attending groups were encouraged to share ~~learnings~~learning and key messages with other members of the community who were unable to attend.

PLA groups were led by 12 facilitators (6 males, 6 females) – two in each cluster – recruited from the intervention areas. They received training on group facilitation and basic health messages related to non-communicable disease prevention and control, with particular focus on T2DM. Facilitators were equipped with a picture card, flip chart and PLA manual with topic-specific information and suggested group activities. The intention was for groups to cover one topic in each meeting, but due to COVID-19 adaptations, multiple topics needed to be covered in single meetings. Topics included information on T2DM, its causes, symptoms, prevention, and control. The PLA manual was adapted from the DMagic trial, aligned with standard recommendations for awareness, prevention and control of T2DM and updated to incorporate information about preventing COVID-19. Male groups were led by a male facilitator and female groups were led by a female facilitator. Facilitators were salaried and each was expected to coordinate and facilitate an average of 18 group meetings per month. Facilitators were mentored and supported by two participatory group coordinators and, a District Manager based locally, and a Senior Group Intervention Manager based in Dhaka.

Piloting & interruption

We conducted a 1-month pilot in intervention areas in December 2020. Experiences from the pilot were shared with study Community Advisory Groups – groups based in each of the study upazilas and comprised of community stakeholders – who were asked to provide input on the acceptability of implementing the PLA intervention after COVID-19 lockdown. ~~Routine intervention implementation began in January 2021.~~Details of this community consultation are published elsewhere and describe key adaptations to the intervention, including restrictions on group size and the implementation of COVID-19 screening and hygiene measures.[13] Routine intervention implementation began in January 2021. A further government COVID-19 lockdown was imposed between April 2021 and August 2021, during which time all field activities, including intervention implementation were paused. Intervention implementation resumed in November 2021 and then continued uninterrupted until September 2022.

Control

The 6 control clusters received usual care, which in this context is little or no preventative public health campaigning and care seeking in government or private facilities, often associated with out-of-pocket payment for diagnostics, consultations, and treatments. Commitment to deliver the intervention to all clusters was made at the public orientation meeting (reflecting the original stepped-wedge design). Control clusters started to receive the intervention after the trial evaluation endline survey in November 2022.

Procedures

Sampling for evaluation

We used a three-step sampling approach. Prior to randomisation, we purposefully selected two to six evaluation villages from a central area in each cluster, with the aim to have between 800-1000 households present, and assuming an average of two people aged ~~over~~ >30 years in each household. Eligible villages were those which do not sit on a border with a neighbouring study cluster, do not act as a major trading centre or administrative centre, and had a minimum of 50 households. The list of villages, and their estimated population sizes was derived from the 2011 Bangladesh census, and administrative maps.

A sampling frame of all the households with at least one non-pregnant permanent resident aged 25 years or above within the selected evaluation villages was generated by household census completed in the evaluation villages in January 2020. Each member of the household aged 30 years or older was listed and a sample of households with at least one adult aged ≥30 years was selected using simple random sampling. Next, a one adult aged ≥30 years was selected for inclusion in the survey, again using simple random sampling. New samples of households and individuals were generated from the same sampling frame (same villages) for the baseline survey (4th January to 25th

February 2021) and endline survey (5th September and 15th November 2022). This general population sample was used to evaluate population-wide intervention impacts.

In addition, individuals aged 30 years or older identified with intermediate hyperglycaemia in the 2021 baseline survey were followed-up at endline (i.e. an intermediate hyperglycaemia cohort) and this sample was used to evaluate intervention impacts on diabetes incidence in a high-risk population. Therefore, we describe the total endline collection as including a cross-sectional random population sample, and a cohort sample of those with intermediate hyperglycaemia at baseline. Some individuals with baseline intermediate hyperglycaemia were also randomly sampled in the cross-sectional survey at endline – these individuals were considered to belong to both samples.

Survey Procedures

All sampled individuals in a single village were informed of the requirements of the study and were requested to prepare for data collection in the morning of a specified day following an overnight fast. Data collection took place at respondents' homes or at a convenient location in their village.

Data were collected by six teams of locally recruited fieldworkers comprised of one male and one female with at least secondary education. They underwent 10 days training on survey methods and how to take physical measurements, followed by one-week supervised field practice and piloting in non-study villages in Faridpur district. Data collectors were supervised by two field supervisors with experience in survey methods. Questionnaire data were gathered in Bangla on Android tablets using ODK Collect.

Individual Questionnaire

Fieldworkers used a structured survey instrument adapted from the WHO Stepwise tool~~[10] and 2014 Bangladesh Demographic and Health Survey[7] to measure the background demographic and socio-economic characteristics, lifestyle and behavioural risk factors, T2DM awareness indicators and health seeking behaviour and costs of care seeking among study participants. The Patient Health Questionnaire-9 (PHQ-9) and General Anxiety Disorder-7 (GAD-7) mental health screening tools were used to measure depression and anxiety, respectively.[11, 12]~~[15] and 2014 Bangladesh Demographic and Health Survey[12] to measure the background demographic and socio-economic characteristics, lifestyle and behavioural risk factors, T2DM awareness indicators and health seeking behaviour and costs of care seeking among study participants. Given the established links between diabetes, depression[16] and anxiety, we used the Patient Health Questionnaire-9 (PHQ-9) and General Anxiety Disorder-7 (GAD-7) mental health screening tools to measure depression and anxiety, respectively.[17, 18] Both PHQ-9 and GAD-7 have been applied previously in Bangladesh and similar populations.[19, 20]

Physical measurements

Fieldworkers measured blood glucose using the One Touch Verio Flex Glucometer (Lifescan, Inc., Milpitas, USA) in whole blood obtained by finger prick from capillaries in the middle or ring finger. Individuals with a self-reported prior medical diagnosis of T2DM were invited to provide random blood glucose readings whereas all other participants were asked to provide readings after an over-night fast. All individuals without a prior diagnosis of T2DM then received a 75g glucose load dissolved in 250 ml of water and had a repeat capillary blood test within 5 minutes of 120 minutes post ingestion to determine glucose tolerance status and differentiate between individuals with intermediate hyperglycaemia and those with T2DM according to WHO criteria (Supplementary Table 2).

Blood pressure was measured using the OMRON HBP 1100 Professional Blood Pressure Monitor (Kyoto, Japan). Two measurements were taken at approximately 5-minute intervals and the respondent's blood pressure obtained by averaging these measurements. Measurements of height, weight, and waist and hip girth were taken with light clothes without shoes and using standard

methods~~.~~ .[21] Survey participants were provided with their blood glucose and blood pressure results and were signposted to health services for further testing, advice and treatment.

Data Management & Quality Control

Data quality control was ensured through ODK form design, supervisor observation, and data checking in Dhaka, after which data were uploaded to a cloud server where project data leads (King/Beard) ran Stata scripts for further data verification.

The same procedures were applied at the baseline and endline surveys. Data collectors, supervisors and managers were unaware of randomisation assignments at baseline but field staff may have been able to deduce assignment during data collection at endline. Access to endline data was restricted until collection was complete, at which point the data were available for blinded analysis by the PI (EF) and trial statistician (AC). Data collection, management and analytical procedures were monitored by an independent Data Monitoring Committee (DMC). Trial management was also reviewed by an independent Trial Steering Committee (TSC).

Process evaluation

~~Process evaluation data will be published in detail elsewhere. Briefly, we conducted a mixed methods process evaluation following the MRC guidelines to describe the context of intervention and control areas, implementation of the intervention, and mechanisms of how the intervention might have affected health outcomes. In intervention areas, we used project monitoring documents, group meeting minutes, observations, interviews and focus group discussions with the intervention team and group members.[13] Qualitative interviews and discussions were conducted by a process evaluation officer, transcribed and translated to English and analysed using the framework approach.[14]~~

Process evaluation data has been published in detail elsewhere.[22] Briefly, we conducted a mixed methods process evaluation following the MRC guidelines to describe the context of intervention and control areas, implementation of the intervention, and mechanisms of how the intervention might have affected health outcomes. In intervention areas, we used project monitoring documents, group meeting minutes, observations, interviews and focus group discussions with the intervention team and group members.[23] Qualitative interviews and discussions were conducted by a process evaluation officer, transcribed and translated to English and analysed using the framework approach.[24]

Outcomes

Our primary outcome was the combined prevalence of intermediate hyperglycaemia (i.e. impaired fasting glucose or impaired glucose tolerance) and T2DM among adults aged 30 years or older. We used WHO definitions and blood glucose cut-offs for normoglycaemia, impaired fasting glucose, impaired glucose tolerance and T2DM as summarised in Supplementary Table 2. Individuals with a self-reported prior diagnosis of T2DM were classified as living with diabetes in our analysis, irrespective of blood glucose readings. The primary outcome is defined only for those in the cross-sectional sample at endline.

Secondary outcomes are defined in Supplementary Table 3 and relate to self-awareness of diabetic status, knowledge of T2DM, utilisation of diabetic services, depression, anxiety and common non-communicable disease risk factors. We also calculated the cumulative incidence of T2DM among the cohort of individuals with intermediate hyperglycaemia.

We also included several pre-specified exploratory and explanatory outcome measures designed to identify potential mechanisms of intervention effect on population behaviours, health and wellbeing. Among individuals with a self-reported prior diagnosis of T2DM, these included: diabetes control (random blood glucose $\leq$11.1 mmol/L), self-reported diabetes-related health complications,

at least monthly blood glucose testing, perceptions of social stigma, measures of family support or abuse related to diabetes diagnosis, and psychological distress and ability to self-manage diabetes assessed using the Appraisal of Diabetes Scale (ADS).[~~15~~25] Among all participants, we measured self-rated health (score 0-100 on a visual analogue scale), tobacco use, betel nut use, sedentary time, time engaged in brisk walking within the previous 7 days, and perceptions of social norms related to dietary practices and female physical activity.

Field Code Changed

Sample size and statistical analysis

Due to COVID-19 adaptations, we updated our sample size calculation in 2022 using methods described by Copas and Hooper (2020).[~~16~~26] With an average of 116 participants per cluster in the baseline survey (n=1392), we estimated that 125 participants per cluster in the endline survey would give 78% power to detect a 30% reduction in the primary outcome of combined prevalence of T2DM and intermediate hyperglycaemia, assuming a baseline primary outcome prevalence of 40%, an intra-cluster correlation of 0.02 and an estimated autocorrelation between baseline and endline of 0.4. We therefore sampled 132 respondents for the endline survey, to account for non-participation (n=1584). Our goal was to also collect data from a cohort of participants with intermediate hyperglycaemia at baseline, giving an endline target sample of 1868.

The main analysis of the primary outcome includes all individuals who provided blood glucose measures at endline from the cross-sectional survey. For the cumulative incidence secondary outcome, analysis includes all individuals in the cohort for whom a baseline blood glucose measurement of intermediate hyperglycaemia was taken and an endline blood glucose measurement was taken. We did not impute any values for missing data since key predictors we might use for imputation (e.g. age, sex, baseline hyperglycaemia) were already included in model adjustments.

The primary analyses for each outcome were undertaken on an intention-to-treat basis. For each outcome, unless otherwise specified, we conducted an 'unadjusted' and 'adjusted' analysis. All analyses included baseline data alongside the endline data to gain precision and account for any baseline imbalance. The 'unadjusted' analysis included adjustment only for the baseline prevalence of the outcome at the cluster level. 'Adjusted' analysis included further adjustment for age as a linear and quadratic term, gender, and baseline hyperglycaemia, as specified *a priori* as likely predictors of intermediate hyperglycaemia and T2DM.

We applied a two-stage cluster summary approach for all outcomes because of the modest number of clusters.~~[17]~~[27] In the first stage of the unadjusted analysis a cluster summary of the outcome was calculated for each cluster at baseline and endline. In the second stage the endline cluster summary values were analysed using linear regression including trial arm and baseline cluster summary value as predictors. The coefficient for trial arm represents an intervention effect on the difference scale.

For the adjusted analysis, we first calculated adjusted cluster summary values at baseline and endline by, separately for each time point, fitting a model to predict the outcome including as predictors age, gender and (for endline model only) baseline survey participation, but excluding trial arm and ignoring the clustering in the data. The outcome was then predicted for all participants (as a probability for binary outcomes) and the cluster mean of the predicted values was calculated for all clusters. Next a residual term was calculated for each cluster – the observed cluster mean minus the mean predicted value. In the second stage the residuals at endline were modelled using linear regression including trial arm and baseline residual as predictors. The coefficient for trial arm represents the adjusted intervention effect on the difference scale. We consider the adjusted analysis primary for all outcomes.

Primary analysis of the primary outcome was conducted blind and independently by the trial PI (EF) and trial statistician (AC) who reported these results to the DMC and Chair of the TSC, after which the identities of the trial arms were revealed, and analysis continued un-blinded.

Pre-specified exploratory analyses of possible interactions between intervention and the following characteristics were undertaken using individual level analysis of endline data: (1) gender, (2) household wealth, (3) age, (4) village size (~~smaller~~less than mean village size vs. ~~larger villages~~greater than or equal to mean village size), and (5) inclusion in baseline survey. Specifically, we used independence estimating equations with robust standard errors through the use of the complex survey 'svy' functions in Stata and added the interaction term between allocated group and subgroup variable into a logistic regression (odds) model.

To assess effects of possible misclassification in the primary outcome based on self-reported diagnoses, we also conducted sensitivity analysis of intervention effect on the primary outcome whereby a) all cases of self-reported T2DM were dropped from the analysis and b) all cases were defined based on blood glucose measures only (i.e. disregarding self-reported diagnoses) where individuals with self-reported diabetes with random blood glucose values of ≥11.1 mmol/L were classified as living with diabetes and those with values <11.1 mmol/L were classified as normoglycaemic.

To assess effects of possible blood glucose measurement bias we assessed intervention impact on continuous blood glucose measurements and by separately applying different arbitrary fasting blood glucose cut-offs of 5·5 mmol/L, 6·3 mmol/L, and 7·8 mmol/L and 2-h blood glucose cut-offs of 6·8 mmol/L and 10·4 mmol/L for classifications of intermediate hyperglycaemia or T2DM.

Total cost and cost-effectiveness analysis of the PLA intervention was conducted from a programme provider perspective. Costs were collected prospectively from project accounts and input into a customised excel-based costing tool designed for this purpose. Time horizon for costing was 36 months, including 16 months start-up and 20 months implementation. Costs were estimated. The incremental cost-effectiveness ratio (ICER) was calculated in terms of cost per 1-mmHg reduction in diastolic blood pressure (statistically significant secondary outcome). All costs were adjusted for inflation, discounted at 3% per year and converted to 2022 US dollar (US$) using the exchange rate of 1US$=91.75 Bangladeshi Taka.[~~18~~28] A detailed analysis will be presented in a separate publication.

Field Code Changed

Statistical analysis was conducted in ~~Stata~~StataSE version 15 and StataMP version 18, and a 5% significance level used for all analyses.

Patient & public involvement

~~Members of the public and community representatives were engaged in a community orientation meeting at the start of the study and in regular community advisory groups to inform the processes and methods used in the study.~~

Members of the public and community representatives were engaged in a community orientation meeting at the start of the study and in regular community advisory groups to inform the processes and methods used in the study. This included representatives from each of the study upazillas acting as a link between the project team and communities and providing direct input into the design of study tools and methods, intervention delivery (including interruptions and re-starting PLA groups in the context of COVID-19[13]), and, at the end of the trial, in dissemination and interpretation of study findings.

Role of the funding source

The funder of the study had no role in study design, data collection, data analysis, data interpretation, or writing the report.

Results

Baseline data were collected across all 12 study clusters from 1392/1584 (87.9%) individuals, of whom 326 individuals were identified with intermediate hyperglycaemia. Endline data were collected from 1343/1584 (84.8%) randomly sampled individuals, and 272/326 (83.4%) of the individuals with intermediate hyperglycaemia at baseline (Figure 1). Sociodemographic characteristics and blood glucose classifications in the baseline and endline random survey samples by study arm are shown in Table 1.

Responders at baseline and endline were more likely to be female (baseline: 92.7% female vs 80.7% male, p<0.001; endline: 89.3% female vs. 78.7% male, p<0.001) than non-responders. Male responders were older than male non-responders (baseline mean difference 4.5 years, p=0.0026; endline mean difference 1.0 year, p=0.1333) and female responders were younger than female non-responders (baseline mean difference=2.4 years, p=0.1464; endline mean difference 2.2 years, p=0.0009). The same patterns of non-response were observed across trial arms.

A total of 108 groups were held by 12 facilitators, with each facilitator responsible for eight to eleven groups per month. Each group had an average of 26 participants and~~,~~ based on our COVID protocol, groups were divided into two separate meetings of around 13 participants in each. Around 60% of the attendees were between 30-40 years old, and the remaining were over 50 years of age. COVID-19 disruptions prevented fidelity to the PLA intervention approach and limited the duration of the intervention. Individual and household actions to prevent diabetes were reported ~~to have been undertaken~~, and group members indicated that they had learned enough information over 10 months to implement individual change. However, PLA group attendance was low after the wider community meeting and data suggest that restricting the number of people attending groups, masking, social distancing, and the discomfort around household visits in a COVID-19 pandemic context did not engender community or group cohesion necessary to trigger mechanisms of community-wide actions. Table 2 shows that 31.6% of the intervention arm sample reported ever participating in a PLA group and those who did participate reported attending a median of 2 out of a maximum of 13 meetings. Approximately 1 in 4 respondents who did not attend PLA groups reported knowing someone who did and 16% of respondents participated in the wider community meeting.

There was no evidence of an intervention effect on the prevalence of intermediate hyperglycaemia and T2DM (adjusted difference, intervention minus control -1.19 (-10.74, 8.36), p=0.7838) (Table 3). Findings were similar in pre-specified sensitivity analyses (Supplementary Table 4).

Amongst our secondary outcomes, large increases in ability to report one or more valid causes, symptoms, complications and strategies for prevention and control of T2DM were observed in the intervention arm relative to control (Table ~~4~~3). PLA intervention was associated with a significant reduction in diastolic blood pressure (-2.98 (-5.55, -0.41), p=0.0277), but little effect on systolic blood pressure, and possible reductions in the prevalence of hypertension (-7.40 (-16.03, 1.24), p=0.0845) and abdominal obesity (-15.23 (-33.02, 2.56), p=0.0847). Two-year cumulative incidence of T2DM among the intermediate hyperglycaemia cohort was lower in intervention clusters relative to control, but the difference was not statistically significant (-8.60 (-20.09, 2.89), p=0.1265). A potential small negative intervention impact on physical activity is noted.

438 **Figure 1: Trial consort profile**

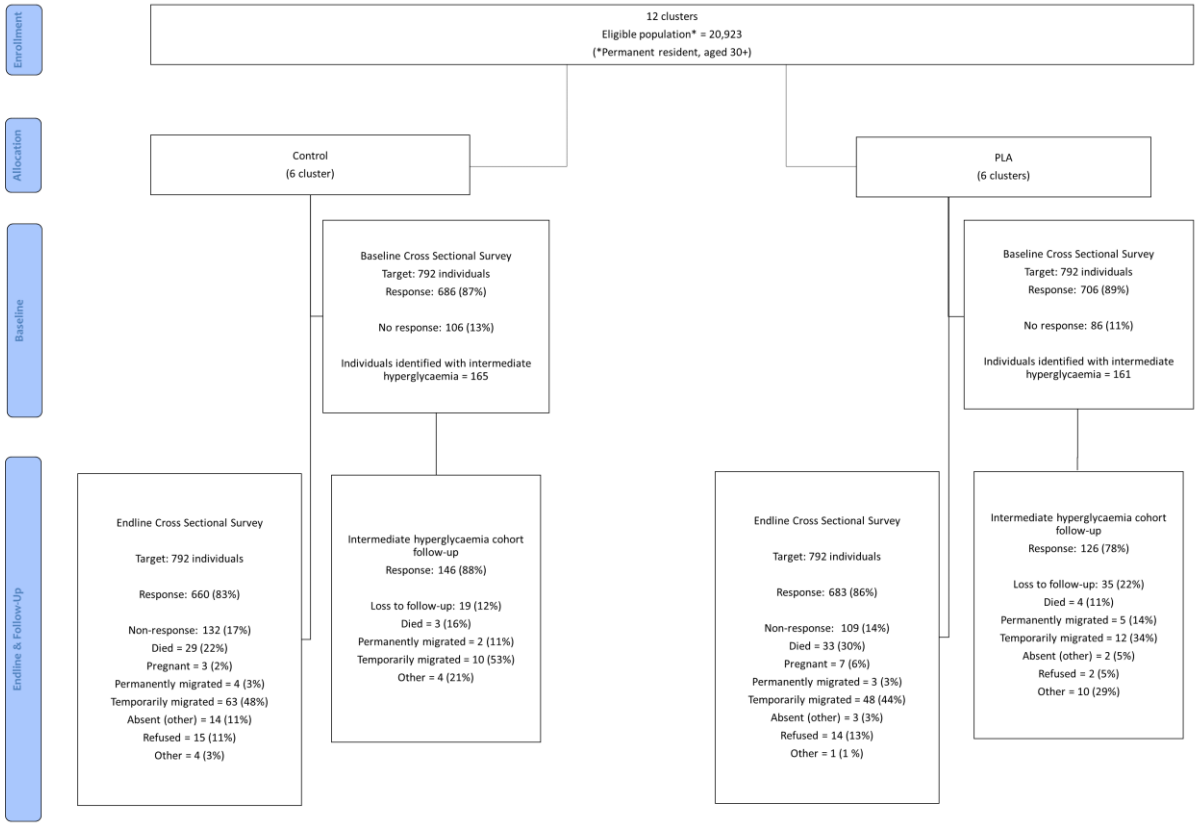

439

Table 1: Count and cluster mean sociodemographic characteristics and blood glucose classification by trial arm at baseline and endline random population samples.

*IFG=Impaired Fasting Glucose; IGT=Impaired Glucose Tolerance

		Baseline		Endline	
		Control	Intervention	Control	Intervention
Clusters		6	6	6	6
Average cluster population aged ≥30 years (sd)		1750.7 (330.8)	1782.5 (166.7)	1818.7 (342.2)	1840.3 (169.2)
Average number of households with eligible members per cluster (sd)		975.7 (76.9)	971.0 (151.8)	990.3 (76.9)	991.4 (153.9)
Total (survey)		686	706	660	683
Age	30-39 years	198 (28.9%)	216 (30.6%)	116 (17.6%)	129 (18.9%)
	40-49 years	183 (26.7%)	169 (23.9%)	196 (29.7%)	163 (23.9%)
	50-59 years	123 (17.9%)	121 (17.1%)	161 (24.4%)	162 (23.7%)
	60-69 years	118 (17.2%)	125 (17.7%)	90 (13.6%)	119 (17.4%)
	70-100 years	64 (9.3%)	75 (10.6%)	97 (14.7%)	110 (16.1%)
Sex	Male	248 (36.2%)	268 (38.0%)	243 (36.8%)	264 (38.6%)
	Female	438 (63.9%)	438 (62.0%)	417 (63.2%)	419 (61.4%)
Education	None	369 (53.8%)	457 (64.7%)	364 (55.2%)	387 (56.7%)
	Primary	231 (33.7%)	192 (27.2%)	235 (35.6%)	231 (33.8%)
	Secondary	71 (10.4%)	44 (6.2%)	47 (7.1%)	48 (7.0%)
	Tertiary	15 (2.2%)	13 (1.8%)	14 (2.1%)	17 (2.5%)
Illiterate	Literate	385 (56.1%)	354 (50.1%)	359 (54.4%)	408 (59.7%)
	Illiterate	301 (43.9%)	352 (49.9%)	301 (45.6%)	275 (40.3%)
Marital status	Married	586 (85.4%)	596 (84.4%)	554 (83.9%)	545 (79.8%)
	Not married	100 (14.6%)	110 (15.6%)	106 (16.1%)	138 (20.2%)
Religion	Muslim	660 (96.2%)	688 (97.5%)	614 (93.0%)	670 (98.1%)
	Other	26 (3.8%)	18 (2.6%)	46 (7.0%)	13 (1.9%)
Occupation	Not working	468 (68.2%)	457 (64.7%)	448 (67.9%)	428 (62.7%)
	Manual labour	183 (26.7%)	224 (31.7%)	187 (28.3%)	229 (33.5%)
	Non-manual labour	35 (5.1%)	25 (3.5%)	25 (3.8%)	26 (3.8%)
Wealth quintile	Most poor	109 (15.9%)	170 (24.1%)	131 (19.9%)	141 (20.6%)
	Very poor	148 (21.6%)	148 (21.0%)	145 (22.0%)	125 (18.3%)
	Poor	143 (20.9%)	118 (16.7%)	153 (21.2%)	121 (17.7%)
	Less poor	167 (24.3%)	121 (17.1%)	119 (18.0%)	142 (20.8%)
	Least poor	119 (17.4%)	149 (21.1%)	112 (17.0%)	154 (22.6%)
Total (diabetes outcomes)		681	704	660	683
Diabetes outcomes	Normal	374 (54.9%)	435 (61.8%)	331 (50.2%)	372 (54.5%)
	IFG*	30 (4.4%)	34 (4.8%)	31 (4.7%)	45 (6.6%)
	IGT*	135 (19.8%)	127 (18.0%)	154 (23.3%)	160 (23.4%)
	Diabetes	142 (20.9%)	108 (15.3%)	144 (21.8%)	106 (15.5%)

Table 2: PLA intervention coverage process indicators by trial arm.

Intervention exposure	Allocation	
	Control	Intervention
Ever participated in PLA group (%)	0 (0%)	216 (31.6%)
Median number of meetings attended (if>0), (IQR; min; max)	NA	2 (2-5; min.=1, max.=13)
Respondent knows someone who attended PLA group if respondent did not attend (%)	0 (0%)	115 (24.6%)
Participated in community meeting (%)	1 (0.2%)	109 (16.0%)

Formatted Table

Formatted Table

Table 3 Frequencies and proportions, or means/medians, and absolute (coefficient) effects and 95% confidence interval comparing primary and secondary outcome measures between trial arms (intervention – control)

OUTCOMES		Baseline		Endline		Crude Difference, mean or % (95%CI)*	Adjusted Difference, mean or % (95%CI)**
		Control	Intervention	Control	Intervention		
**PRIMARY**							
**Combined prevalence of intermediate hyperglycaemia and diabetes**		307 (45.1%)	269 (38.2%)	329 (49.9%)	311 (45.5%)	-1.85 (-11.63, 7.92,); p=0.6777	-1.19 (-10.74, 8.36,); p=0.7838
**SECONDARY**							
**Blood pressure**	**Systolic blood pressure (mmHg), mean (sd)**	133.5 (22.6)	133.1 (22.1)	127.0 (20.7)	126.8 (19.1)	0.02 (-3.20, 3.24); p=0.9900	-0.15 (-3.33, 3.04); p=0.9176
	**Diastolic blood pressure (mmHg), mean (sd)**	75.7 (11.4)	79.2 (11.4)	75.8 (11.1)	77.6 (10.8)	-2.78 (-5.24, -0.32,); p=0.0308	-2.98 (-5.55, -0.41); p=0.0277
	**Hypertension, n (%)**	274 (39.9%)	274 (38.8%)	236 (35.8%)	196 (28.7%)	-6.47 (-14.87, 1,93,); p=0.1153	-7.40 (-16.03, 1.24,); p=0.0845
**Overweight & obesity**	**Body Mass Index (BMI), mean (sd)**	23.4 (4.0)	22.7 (3.9)	23.0 (4.0)	22.8 (3.7)	0.38 (-0.30, 1.07); p=0.2404	0.39 (-0.39, 1.17); p=0.2837
	**Overweight or obese, n (%)**	346 (50.4%)	319 (45.3%)	318 (48.2%)	304 (44.5%)	2.59 (-4.84, 10.0), p=0.4504	3.04 (-4.75, 10.83); p=0.4008
	**Waist:Hip ratio, mean (sd)**	0.90 (0.06)	0.89 (0.07)	0.91 (0.05)	0.90 (0.07)	-0.01 (-0.04, 0.02); p=0.4133	-0.01 (-0.04, 0.02); p=0.4420
	**Abdominal obesity, n (%)**	471 (68.7%)	425 (60.2%)	529 (80.2%)	436 (63.8%)	-15.29 (-33.94, 3.36); p=0.0966	-15.23 (-33.02, 2.56); p=0.0847
**Dietary diversity score, mean (SD)**		5.4 (2.2)	5.0 (1.5)	4.7 (1.6)	4.7 (1.4)	-0.10 (-0.59, 0.39); p=0.6566	-0.09 (-0.59, 0.41); p=0.7000
**Log transformed minutes spent engaged in physical activity per week, mean (sd)**		6.4 (0.8)	6.3 (1.0)	6.2 (0.7)	5.9 (0.9)	-0.26 (-0.53, 0.01); p=0.0569	-0.25 (-0.54, 0.04); p=0.0871
**Diabetes knowledge**	**Ability to report one or more valid *causes* of diabetes (%)**	346 (50.4%)	291 (41.2%)	336 (50.9%)	576 (84.3%)	33.73 (17.67, 49.79); p=0.0010	34.07 (18.05, 50.10); p=0.0010
	**Ability to report one or more valid *symptoms* of diabetes (%)**	417 (60.8%)	321 (45.5%)	374 (56.7%)	595 (87.1%)	35.4 (18.21, 52.59); p=0.0012	35.82 (18.67, 52.98); p=0.0011
	**Ability to report one or more valid *complications* of diabetes (%)**	213 (31.1%)	225 (31.9%)	196 (29.7%)	534 (78.2%)	48.17 (28.70, 67.64); p=0.0003	48.66 (29.32, 68.00); p=0.0003
	**Ability to report one or more valid ways to *prevent* diabetes (%)**	407 (59.3%)	385 (54.5%)	451 (68.3%)	601 (88.0%)	21.26 (10.51, 32.01); p=0.0015	21.82 (11.06, 32.58); p=0.0013
	**Ability to report one or more valid ways to *control* diabetes (%)**	453 (66.0%)	410 (58.1%)	498 (75.5%)	606 (88.7%)	14.75 (3.64, 25.86); p=0.0149	15.27 (4.16, 26.39); p=0.0125
**PHQ-9 score ≥10 (moderate or severe depression), n (%)**		51 (7.4%)	33 (4.7%)	27 (4.1%)	24 (3.5%)	-0.12 (-2.93, 2.69); p=0.9232	-0.40 (-3.22, 2.42); p=0.7553
**GAD-7 score ≥10 (moderate or severe anxiety), n (%)**		29 (4.2%)	31 (4.4%)	28 (4.2%)	18 (2.6%)	-1.57 (-4.63, 1.49); p=0.2762	-1.72 (-4.83, 1.38); p=0.2409
**Self-awareness of diabetic status among individuals identified with diabetes by blood glucose measures, n (%)**		70 (49.3%)	47 (43.5%)	69 (47.9%)	51 (48.1%)	0.77 (-20.28, 21.81); p=0.9361	3.61 (-14.89, 22.10); p=0.6694
**Utilisation of services for treatment or advice for diabetes among individuals with prior diagnosis of diabetes**		63 (90.0%)	40 (85.1%)	66 (95.7%)	46 (90.2%)	-4.33 (-15.66, 6.99); p=0.4093	-3.92 (-14.63, 6.79); p=0.4288
**Two-year diabetes incidence among individuals with intermediate hyperglycaemia at baseline, n (%)**				25 (17.1%)	12 (9.5%)	-8.60 (-20.91, 3.72); p=0.1509	-8.60 (-20.09, 2.89); p=0.1265

* Adjustment for baseline outcome measure at the cluster level. **Adjusted for cluster-level baseline outcome measure, gender and age as linear and quadratic terms. Intraclass correlation (ICC) of primary outcome at endline is 0.01.

Formatted Table

Table 4: Frequencies, proportions, means/medians and absolute (coefficient) effects and 95% confidence interval comparing explanatory outcome measures by trial arm (intervention – control)

OUTCOMES		Baseline		Endline		Crude Difference (95%CI)*	Adjusted Difference (95%CI)**
		Control	Intervention	Control	Intervention		
Self-rated health, mean (sd)		76.1 (17.6)	86.4 (12.8)	69.2 (16.6)	78.0 (18.4)	8.33 (1.91, 14.75); p=0.0167	9.12 (2.58, 15.6); p=0.0116
Daily smoking or use of smokeless tobacco (%)		218 (31.8%)	275 (39.0%)	242 (36.7%)	259 (37.9%)	-2.26 (-10.87, 6.35); p=0.5670	-1.44 (-11.1, 8.23); p=0.7439
Betel nut use (%)		207 (30.2%)	261 (37.0%)	212 (32.1%)	235 (34.4%)	1.26 (-11.96, 14.5); p=0.8344	0.25 (-12.61, 13.1); p=0.9657
Log sedentary time in previous 24 hours, median (IQR)		4.7 (4.4-5.0)	4.7 (4.1-5.2)	4.7 (4.1-5.1)	5.0 (4.8-5.2)	0.26 (0.05, 0.47); p=0.0206	0.24 (0.0, 0.43); p=0.0225
Log time engaged in brisk walking, median (IQR)		5.3 (4.8-6.0)	5.1 (4.8-5.6)	5.3 (4.8-5.5)	5.2 (4.8-5.5)	-0.15 (-0.52, 0.22); p=0.3777	-0.13 (-0.49, 0.24); p=0.4454
Among individuals with prior diagnosis of diabetes	Diabetes control (%)	15 (21.4%)	7 (14.9%)	15 (21.7%)	15 (29.4%)	3.56 (-12.16, 19.28); p=0.6208	1.80 (-15.00, 18.60); p=0.8139
	Diabetes affect (ADS) score, mean (sd)	14.9 (3.3)	13.8 (3.2)	16.1 (3.8)	16.5 (3.5)	0.90 (-1.47, 3.26); p=0.4134	0.78 (-1.58, 3.14); p=0.4716
	Diabetes-related complications (%)	55 (78.6%)	28 (59.6%)	60 (87.0%)	33 (64.7%)	-22.54 (-48.23, 3.15); p=0.0784	-22.46 (-48.22, 3.30); p=0.0801
	At least monthly blood glucose testing (%)^	17 (24.3%)	17 (36.2%)	18 (26.1%)	17 (33.3%)	25.06 (-23.55, 73.68); p=0.2735	19.97 (-29.74, 69.68); p=0.3871
	Stigma 1: Perceived inability to fulfil responsibilities+	31 (44.3%)	12 (25.5%)	32 (46.4%)	25 (49.0%)	22.90 (-18.29, 64.09); p=0.2402	23.18, (-17.25, 63.62); p=0.2269
	Stigma 2: Perceived to be seen as a lesser person+	18 (25.7%)	4 (8.5%)	13 (18.8%)	14 (27.5%)	18.41 (-35.51, 72.32); p=0.4598	18.08 (-32.23, 68.39); p=0.4373
	Stigma 3: Embarrassed in social situations+	29 (41.4%)	5 (10.6%)	20 (29.0%)	17 (33.3%)	38.40 (-8.21, 85.01); p=0.0953	37.17 (-8.32, 82.65); p=0.0976
	Stigma 4: Ashamed of having diabetes+	18 (25.7%)	4 (8.5%)	11 (15.9%)	9 (17.7%)	8.79 (-16.79, 34.38); p=0.4567	10.42 (-17.29, 38.13); p=0.4171
	Feels supported by family in managing diabetes~	58 (82.9%)	28 (59.6%)	49 (71.0%)	36 (70.6%)	-5.64 (-27.55, 16.27); p=0.5747	-5.05 (-27.15, 17.04); p=0.6173
	Ever experienced physical, social, or mental abuse due to diabetic status	27 (38.6%)	5 (10.6%)	20 (29.0%)	17 (33.3%)	7.45 (-31.09, 45.99); p=0.6723	6.81 (-32.38, 46.00); p=0.7035
Social norms: Perceived negative feelings or family or social reactions to:	1) Going on a morning walk alone (women only)	35 (8.0%)	87 (19.9%)	26 (6.2%)	105 (25.1%)	4.47 (-5.12, 14.06); p=0.3192	4.19 (-5.77, 14.1); p=0.3662
	2) Going for a morning walk with a female relative (women only)	10 (2.3%)	26 (5.9%)	15 (3.6%)	74 (17.7%)	7.87 (-2.43, 25.2); p=0.3302	8.12 (-8.90, 25.15); p=0.3084
	3) Eating less than usual or refusing oily or sugary foods and drinks at social gatherings	494 (72.0%)	580 (82.2%)	462 (70.0%)	612 (89.6%)	14.36 (2.40, 26.32); p=0.0237	14.59 (2.25, 26.93); p=0.0254
	4) Providing healthy snacks to guests	392 (57.1%)	305 (43.2%)	356 (53.9%)	392 (57.4%)	4.02 (-28.58, 36.62); p=0.7866	4.07 (-28.49, 36.63); p=0.7839

* Adjustment for baseline outcome measure at the cluster level. **Adjusted for cluster-level baseline outcome measure, gender and age as linear and quadratic terms. ^Missing data for 9 cases – 1 in control arm at baseline, 4 in each arm at endline. +Missing data for 7 cases – 3 in intervention arm and 1 in control arm at baseline, 2 in intervention arm and 1 in control arm at endline ~Reports at least moderate family support

Formatted Table

There was a significant increase in self-rated health in the PLA arm relative to control and a large but non-significant reduction the prevalence of self-reported diabetes-related health complications (Table 4). A small potential negative intervention effect on sedentary time was observed, as was a significant increase in perceived negative social ~~feelings~~ or family ~~or social~~ reactions to eating less than usual or refusing oily or sugary food and drinks at social gatherings, though wide confidence intervals are noted.

Given the observed intervention effect on diastolic blood pressure, we conducted post-hoc analyses of blood pressure measures. Namely, we evaluated intervention impact on pulse pressure, calculated as the difference between systolic and diastolic blood pressure, and mean arterial pressure, which accounts for flow, resistance and pressure in arteries and is calculated as diastolic blood pressure plus one third of the difference between diastolic and systolic blood pressure. Analysis showed a significant reduction in pulse pressure in intervention clusters relative to control (adjusted difference -1.97 (-3.77, -0.16), p=0.0358). There was also a reduction in mean arterial pressure, though not statistically significant (-1.27 (-3.57, 1.02), p=0.2403). Considering the demonstrated association between anxiety and hypertension in other contexts~~[19]~~[29], and in recognition of the hypothesised effects of PLA via mechanisms of enhanced social support~~[20]~~[30] we further analysed the GAD-7 measure of anxiety as a log-transformed continuous outcome as opposed to the categorical outcome included in our pre-specified analysis. This showed that there was a significant reduction in transformed GAD-7 score (-0.22 (-0.41, -0.04), p=0.0201) in intervention clusters ~~(median 2, IQR 1 to 4)~~ compared to control arm ~~(median 3, IQR 1.5 to 5)~~ at endline ~~even though measures were equivalent at baseline~~. Data from post-hoc analyses are presented in Supplementary Table 5.

There was no evidence of interaction between the intervention and gender, household wealth, age, village size, or inclusion in baseline survey in relation to the primary outcome (Supplementary Table ~~5~~6). Some potential interactions were identified, especially in relation to wealth and outcomes related to diabetes awareness, control, and complications, as well as anxiety and depression. Results suggest intervention effect on incidence of diabetes in the cohort of individuals with intermediate hyperglycaemia at baseline may be modified by wealth index, with poorer individuals benefiting more.

Total and average annual costs of the intervention were US$318,706 and US$157,696, respectively. The total cost per target population (adults ≥30 years) covered was US$12. Staff costs constituted 68% of the total intervention cost, followed by other recurrent costs (e.g., overheads) and COVID-19 protection measures, accounting for 13% and 11%, respectively. Costs of the activities conducted in the intervention start-up period (16 months) accounted for around 12% of total costs. Supplementary table ~~4~~7 provides breakdown of the intervention costs by inputs and activities. ICER was US$4.2 per 1-mmHg reduction in diastolic blood pressure, ranging from $2.2 to $30.3 using the confidence interval around the effect size.

Discussion

We evaluated the effects of facilitated PLA community mobilisation on T2DM and non-communicable disease risk among a general population when horizontally scaled-up and in a dynamic context of COVID-19 in rural Bangladesh. There is no evidence of intervention effect on the primary outcome of prevalence of T2DM and intermediate hyperglycaemia. The intervention is associated with large improvements in diabetes knowledge as well as changes in diastolic blood pressure and self-rated health. Potentially important reductions in the prevalence of hypertension and abdominal obesity, as well as self-reported diabetes-related health complications are also noted, though these do not reach statistical significance. Among a higher-risk population cohort (i.e. individuals with intermediate hyperglycaemia at baseline), the direction and magnitude of intervention effect on incidence of diabetes among is very similar to that observed in our former

DMagic trial (-8.7 (-3.5, -14.0))~~[1]~~[6], though given our smaller sample size in D:Clare, the wider confidence intervals included the null.

The absence of a clear intervention effect on the prevalence of intermediate hyperglycaemia and T2DM differs from what we observed in the DMagic trial, which showed large significant effects.~~[1]~~[6] However, the intervention also differed in important ways as a result of COVID-19 disruptions and adaptations. Our process evaluation allows us to examine what worked well and, crucially, to analyse fidelity to the PLA intervention that we planned, and factors affecting this.

There were three main ways that the intervention was not implemented as planned because of COVID-19 disruptions and delays: 1. Phases of the intervention were shorter; 2. Group size, average attendance at PLA groups, interaction between group members and facilitators, and between group members was restricted; 3. Community action was not initiated on a large scale. The lack of pre-existing social cohesion in our study context, which was exacerbated by COVID-19 restrictions, has been reported in other studies as a factor which makes it challenging to mobilise communities for social change.[~~21, 22~~31, 32] The shorter time frame for the first phase of the intervention and interruptions to meeting schedules may also have prevented the development of a core group of members to drive action. ~~We noted a~~A lack of community cohesion in intervention areas ~~which~~ fed into a lack of group cohesion, and when combined with restrictions on group size and less consistency in group membership, it was difficult to develop collective responsibility for prevention and control of diabetes. Our intervention was conducive to participatory learning and awareness raising as reflected in diabetes-related awareness outcomes but may not have allowed sufficient time for collective action which usually occurs after the wider community meeting. Compared to DMagic, where 74% of survey respondents in intervention areas reported ever participating in PLA meetings, repeat attendance was high, and 72% knew someone who participated~~[1], participation in and wider awareness of the intervention was low in D:Clare (Table 2).~~[6], participation in and wider awareness of the intervention was low in D:Clare. Details and results from an in-depth mixed-methods process evaluation of the D:Clare intervention are presented elsewhere.[22]

We have previously described one of the key mechanisms of PLA as a process of enabling individuals to act within enabled, supportive environments.[~~20, 23~~30, 33] It may be that without adequate time devoted to development of community action and implementation of locally agreed strategies our D:Clare implementation was unable to translate raised awareness into effective collective actions. The findings of increased population awareness without impact on T2DM outcomes is similar to the effect of the mHealth health promotion intervention component of DMagic, which similarly lacked opportunity for collective action.~~[24] Nevertheless, the importance of knowledge and awareness as key components of health literacy, which in turn supports individuals and communities in preventing and controlling non-communicable diseases and on overall wellbeing is well-established and recognised as a key enabler to accelerating progress towards the non-communicable disease targets of the Sustainable Development Goals.[25, 26]~~[34] Nevertheless, knowledge and awareness are well-established components of health literacy.[35] This, in turn, supports individuals and communities in preventing and controlling disease, and is necessary to accelerate progress towards global non-communicable disease targets.[35, 36]

We observed a statistically significant reduction in diastolic blood pressure and a suggested reduction in hypertension prevalence in D:Clare, though we note some caution in our certainty of effect due to the number of outcomes tested. Concurrent PLA impact on abdominal obesity and log-transformed anxiety score may have been important mechanisms for this though further exploration of this and the possible role of salt consumption and physical activity changes are needed. While no effects on blood pressure outcomes were observed in the DMagic trial, we did observe similar impacts in our five-year post-randomisation follow-up of DMagic communities.~~[4]~~[9] Raised blood pressure and hypertension is the leading modifiable risk factor for future morbidity and mortality

from cardiovascular diseases, specially heart attacks and stroke[~~27, 28~~37, 38] and blood pressure lowering provides broadly generalisable benefits.~~[29]~~[39] Diastolic blood pressure, pulse pressure and mean arterial pressure are related to peripheral vascular resistance and loss of arterial vasodilatory mechanisms and have been associated with ischaemic stroke.[~~30-32~~40-42]. Data from the Framingham study in the 1990s suggest that even modest reductions in mean population diastolic blood pressure could result in substantial reductions in the prevalence of hypertension and associated risks of cardiovascular events.~~[33]~~[43] The potential to achieve such reductions in blood pressure through population-based interventions of health messaging and community-based strategies such as PLA warrants further exploration, especially in settings that lack screening and treatment for raised blood pressure.

The large, rural, population-based surveys with high response rates at baseline and endline, and the high follow-up of individuals identified with intermediate hyperglycaemia at baseline, with assessment of glycaemic status through fasting and two-hour blood glucose tests are notable strengths of our trial. The inability to ensure blinding of enumerators and participants in the endline survey is a potential weakness. Not all of the same enumerators worked on the endline and baseline surveys and enumerators worked within specific clusters, therefore some inter-observer variation is possible. The use of self-reported measures of behaviours is also a potential weakness of our design and may be subject to recall and social desirability bias and it is plausible that this would vary by trial arm. Measures of physical activity, dietary practice, care-seeking and attitudes must therefore be interpreted with caution. Finally, there was overlap between the timing of baseline data collection and the start of intervention implementation, however, it unlikely that the PLA intervention would have any impacts in this early stage.

The D:Clare intervention ~~costs~~cost $12 per target population (adults ≥30 years~~) covered was US$12.~~). This is slightly higher than DMagic intervention, mainly due to longer implementation period (20 vs 18 months) and cost of COVID-19 protection measures. ICER was US$4.2 per 1-mmHg reduction in diastolic blood pressure, ranging from $2.2 to $30.3. ICER for D:Clare is significantly lower than previous community-based hypertension control interventions.~~[34]~~[44]

Formatted: Underline

Conclusion

Scale-up of PLA for T2DM prevention and control during the evolving COVID-19 pandemic required adaptations to intervention implementation and evaluation approach. There was no evidence of impact of the adapted intervention on blood glucose outcomes, but large and important impacts on diabetes knowledges and likely effects on other health outcomes, including blood pressure. Process evaluation combined with cluster RCT data provide important insights into intervention and implementation features which are necessary for the success of the intervention. Critical to this is providing sufficient time for intervention delivery that allows communities to develop and share collective knowledge and plan and implement collective action. The PLA approach to non-communicable disease prevention and control continues to show promise.

Contributors

EF, the project PI, led in the design of the study, conducted statistical analyses and drafted the manuscript. AK coordinated project activities in Bangladesh, co-led the project and contributed to intervention development and study design. JM led the process evaluation component of the study and contributed to intervention development. TN developed and coordinated the implementation of PLA group activities. CK provided technical coordination of the surveys and data management processes. SKS coordinated quantitative data collection activities. MP contributed to process evaluation data processing and analysis. SAUA gathered and analysed qualitative process ~~and implementation~~ data. JB was responsible for endline survey data processing. NA contributed to intervention development and survey methods. HHB led the economic evaluation component of the project. AC was the trial statistician and contributed to the design, development of the statistical

analysis plan and verification of results. AKAK and KA provide technical oversight of all project activities and facilitated community engagement and intervention development activities. All authors contributed to the interpretation of study findings reported here.

Declaration of interests

We declare no competing interests.

Data sharing

Deidentified data collected for this study and a data dictionary are available from the ~~corresponding author on reasonable request~~UCL Research Data Repository DOI:10.5522/04/28751210.

Acknowledgements

The study team would like to thank the D:Clare Trial Steering Committee (David Beran (Chair), Graham Hitman, Justine Davies, Edward Gregg, Aaron Holiday, Jennifer Thompson, Calum Davey, Audrey Prost and Anthony Costello) and the Data Monitoring Committee (Mohammod Shahidullah (Chair), Meena Daivadanam, and A.T.M Iqbal Anwar) for their valuable contributions to the project. We are grateful to our Community Advisory Groups and Md. Golam Azam for contributions to field activities.

Supplementary Table 1: PLA group design and adaptations in response to COVID-19

Design parameter	Original design	Adaptation
Group composition	Open to all community members, with no restrictions on numbers in meetings. DMagic's average group size was 32.	Open to all community members, but meeting size restricted to maximum of 20 participants or less per meeting.
Meeting frequency	Once a month per village	Twice a month per village, to allow for smaller groups but with the same population coverage
Meeting conduct	Participants sit in a circle, and active participation is encouraged.	Participants were asked to wash their hands on arrival/departure, to wear a mask, and try to keep a minimum of 1-meter distance from group members outside of their households. All materials used during the meeting were wiped with alcohol between meetings.
Facilitator role	Facilitators hold 9 meetings per month	Facilitators held 18 meetings per month. Facilitators travelled using well-ventilated transport (e.g. rickshaw~~)~~) and adhered to infection control practices.
Group communication	Meetings arranged in-person	Phone numbers of group members were collected (after consent) to facilitate communication around meetings, and if needed, to cancel meetings. Numbers were stored locally by facilitators.
Total number of meetings	18 meetings per group delivered over 18 months	Minimum of 13 meetings per group delivered over 20 months. This required more topics to be covered in each meeting.
Community meetings	Large community meetings with several hundred participants held	Smaller stakeholder meetings and use of alternative methods to disseminate group information to wider community, e.g. loudspeaker announcements.

Formatted Table

Formatted Table

618 Supplementary Table 2: Glycaemic definitions and diagnostic criteria used in the D:Clare trial, adapted
619 from WHO 2006

**Definition**		**Diagnostic Criteria**
Normoglycaemia		Fasting plasma glucose ≤6.0 mmol/L
Intermediate Hyperglycaemia	Impaired Fasting Glucose (IFG)	Fasting plasma glucose ≥6.1 mmol/L to <7.0 mmol/L **AND** two-hour post ingestion of 75g glucose load plasma glucose <7.8 mmol/L
	Impaired Glucose Tolerance (IGT)	Fasting plasma glucose <7.0 mmol/L **AND** two-hour post ingestion of 75g glucose load plasma glucose ≥7.8 mmol/L to <11.1 mmol/L
Type 2 Diabetes Mellitus (T2DM)		Fasting plasma glucose ≥7.0 mmol/L **OR*** two-hour post ingestion of 75g glucose load plasma glucose ≥11.1 mmol/L **OR** Self-reported prior medical diagnosis of diabetes

620 **Diabetes cannot be excluded without 2-h post oral glucose load test*
621 *Source: http://apps.who.int/iris/bitstream/10665/43588/1/9241594934_eng.pdf*

622

Formatted Table

623 Supplementary Table 3: D:Clare trial secondary outcome measures and definitions

Outcome	Definition	Denominator	Data type
awareness of diabetic status	self-reported diabetes	endline cross-sectional sample with valid endline blood glucose measurements that meet the classification of diabetes and who complete the endline questionnaire	Binary
physical activity	average time spent engaged in physical activity per week	endline cross-sectional sample with valid self-reported physical activity data	Continuous
blood pressure	diastolic and systolic blood pressure	endline cross-sectional sample with valid blood pressure measurements	Continuous
Hypertension	systolic blood pressure ≥ 140 mmHg or a diastolic blood pressure ≥ 90 mmHg or current treatment with antihypertensive medication	endline cross-sectional sample with valid blood pressure measurements	Binary
Body Mass Index	body mass index (weight (kg)/height(m)^2	endline cross-sectional sample with valid weight and height measurements	Continuous
abdominal obesity	waist:hip ratio (WHR)	endline cross-sectional sample with valid waist and hip measurements	Continuous
dietary diversity	Dietary Diversity Score (DDS)	endline cross-sectional sample with valid DDS data	Continuous
knowledge about diabetes symptoms and complications (five separate outcomes)	proportion of adults aged 30 years and above who are able to: a) name at least one cause of diabetes; b) report at least one symptom of diabetes; c) report at least one complication of diabetes; d) report at least one way to reduce the risk of getting diabetes; e) report at least one way to control diabetes if diagnosed.	endline cross-sectional sample of with valid knowledge data	Binary
utilisation of services for treatment or advice for diabetes	proportion of diabetics with self-reported diagnosis of diabetes reporting current or ever receipt of care or advice from a medical professional	endline cross-sectional sample of known (pre-diagnosed) diabetic individuals	Binary
depression	proportion of adults aged 30 years and above with a PHQ score ≥10 (i.e. moderate to severe depressive symptoms).	endline cross-sectional sample of with valid PHQ data.	Binary
anxiety	proportion of adults aged 30 years and above with a GAD-7 score ≥10.	endline cross-sectional sample of with valid GAD-7 data	Binary
18 month cumulative incidence of T2DM among individuals with intermediate hyperglycaemia at baseline	proportion of adults aged 30 years or older with plasma glucose cut-off categorisations for intermediate hyperglycaemia at baseline who are categorised as T2DM at endline	cohort sample of adults aged 30 years or older with plasma glucose cut-off intermediate hyperglycaemia at baseline and followed-up to endline	Binary

624

Formatted Table
