# Supplementary material for: A cluster randomised controlled trial of community groups using Participatory Learning and Action to prevent and control diabetes and intermediate hyperglycaemia in rural Bangladesh
Source: PLOS Glob Public Health. 2025 Aug 14;5(8):e0005049. doi: 10.1371/journal.pgph.0005049 (PMC12352636; doi:10.1371/journal.pgph.0005049)
Supplement: Attachment — Submitted filename: Review comments.docx [file pgph.0005049.s013.docx]

**Major comments**

**Introduction**

The authors are suggested to include global and national data related to the title and provide a rationale for the study, which is currently missing from the background.

**Method**

In the **randomisation and masking subheading**, the authors stated that ‘Randomisation took place at a project orientation meeting in Faridpur town attended by community representatives and independent observers.’

The authors are suggested to clarify on that whether the community representatives and independent observers were involved in the project at a later stage.

In the **piloting & interruption subheading**, ‘Experiences from the pilot were shared with study Community Advisory Groups – groups based in each of the study upazilas and comprised of community stakeholders – who were asked to provide input on the acceptability of implementing the PLA intervention after COVID-19 lockdown.’

Did the authors revised the PLA intervention based on their feedbacks? Please mention that.

In the **individual questionnaire subheading**, the authors stated that they used a mental health screening tool, but they did not mention their rationale for using it earlier in the manuscript. Was the tool validated from the perspective of Bangladesh? If not, how did they validate it in the context of Bangladesh? Please provide clarification.

In the **physical measurement subheading**, the authors mentioned Measurements of height, weight, and waist and hip girth were taken with light clothes without shoes and using standard methods. Need to cite a reference there.

In the **sample size subheading**, the authors mentioned that (4) village size (smaller vs. larger villages). How did they categorize it? For example: 4 or fewer villages considered a small and more than that considered large villages?

**Result**

From Line 377, page no. 9, the authors showed some data but it’s not clear which table represents the data as they described Table 4 from their previous sentence. The data shown is from Table 3. Please check it.

On page 14, starting from Line 405, the authors discussed post-hoc analyses of blood pressure and presented the results. However, they did not refer to any supplementary table or provide one. Therefore, it is recommended that the authors add a supplementary file with the relevant blood pressure data.

From Line 432, page no. 14, the authors provided breakdown of the intervention costs though the supplementary file 4 showed the sensitivity analysis data.

From Line 432, page no. 14, the authors detailed the intervention costs, while the supplementary file 4 showed the sensitivity analysis data. Please check it. However, Supplementary Table 6 displayed intervention costs data. Please verify and make the necessary corrections.

**Discussion**

The authors referred to Table 2 in the discussion section, but it is recommended not to include result tables in the discussion section.

From Lines 477-481, on page 15, the authors are advised to break it into two sentences, which will make it easier to understand.

**Conclusion**

The authors are suggested to include recommnedation on how to make their project sustainable and how to integrate it into the health policy system.

**Minor comments**

Abstract

Participants

The authors mentioned Adults aged >30 years who permanently reside in study villages, some places they mentioned ≥30 years. Please check this all over the manuscript and correct it.

Intervention

The authors started with 108 PLA groups were created. The authors are suggested to splled it out. Example: One hundred and eight.

Method section of the main manuscript

The authors may include the study duration if the word count permits.

In the main manuscript, in the randomisation and masking subheading, the authors mentioned 12 folded pieces of paper. Please spell out 12 with Twelve.

In the main manuscript, in the intervention subheading, the authors mentioned 108 PLA groups, 9 men’s and 9 women’s in each intervention cluster, please spell out 108 with one hundred and eight.

In sattistical analysis subheading, please add the Stata version.
