# Supplementary material for: A cluster randomised controlled trial of community groups using Participatory Learning and Action to prevent and control diabetes and intermediate hyperglycaemia in rural Bangladesh
Source: PLOS Glob Public Health. 2025 Aug 14;5(8):e0005049. doi: 10.1371/journal.pgph.0005049 (PMC12352636; doi:10.1371/journal.pgph.0005049)
Supplement: Attachment — Submitted filename: 20250318_ReviewerResponses.docx [file pgph.0005049.s016.docx]

10^th^ April 2025

Dear Editors,

We are grateful for the reviews of our manuscript and the opportunity to respond. We provide a point-by-point response to the comments provided and detail any revisions to our manuscript below. We are confident that these reviews and revisions have strengthened our paper.

We look forward to hearing from you in due course.

Yours faithfully,

Professor Ed Fottrell

Editor Comments:

The authors have presented a compelling topic with relevant data, but there are critical issues that need to be addressed. These include some revisions in the introduction, some clarifications in the method, result and conclusion sections. Additionally, some sections of the manuscript require better clarity to improve readability and coherence.

We are grateful to the editorial team and reviewers for their comments and for the opportunity to respond and revise our manuscript accordingly. We provide a point-by-point response below and believe that our revised manuscript if now clearer.

Journal Requirements:

1. Please include a complete copy of PLOS’ questionnaire on inclusivity in global research in your revised manuscript. Our policy for research in this area aims to improve transparency in the reporting of research performed outside of researchers’ own country or community. The policy applies to researchers who have travelled to a different country to conduct research, research with Indigenous populations or their lands, and research on cultural artefacts. The questionnaire can also be requested at the journal’s discretion for any other submissions, even if these conditions are not met.  Please find more information on the policy and a link to download a blank copy of the questionnaire here: <https://journals.plos.org/globalpublichealth/s/best-practices-in-research-reporting>. Please upload a completed version of your questionnaire as

Supporting Information when you resubmit your manuscript.

We have completed the questionnaire.

2.  In the online submission form, you indicated that "Deidentified data collected for this study and a data dictionary are available from the corresponding author on reasonable request.".

All PLOS journals now require all data underlying the findings described in their manuscript to be freely available to other researchers, either 
1. In a public repository, 
2. Within the manuscript itself, or 
3. Uploaded as supplementary information.

This policy applies to all data except where public deposition would breach compliance with the protocol approved by your research ethics board. If your data cannot be made publicly available for ethical or legal reasons (e.g., public availability would compromise patient privacy), please explain your reasons by return email and your exemption request will be escalated to the editor for approval. Your exemption request will be handled independently and will not hold up the peer review process, but will need to be resolved should your manuscript be accepted for publication. One of the Editorial team will then be in touch if there are any issues.

Data are now available in a public repository and we have updated the manuscript to indicate this.

Reviewer #1: Major comments

Introduction

The authors are suggested to include global and national data related to the title and provide a rationale for the study, which is currently missing from the background.

We have expanded the Introduction to provide more global and national context and rationale for our study.

Method

In the randomisation and masking subheading, the authors stated that ‘Randomisation took place at a project orientation meeting in Faridpur town attended by community representatives and independent observers.’

The authors are suggested to clarify on that whether the community representatives and independent observers were involved in the project at a later stage.

We report in our original manuscript (under *Patient & Public Involvement*) that community members were engaged in regular community advisory group meetings to inform the processes and methods used in the study. We have expanded this section to provide further detail on the role of community advisors and our engagement at different stages of the trial, some of which is detailed in other publications cited in our manuscript.

In the piloting & interruption subheading, ‘Experiences from the pilot were shared with study Community Advisory Groups – groups based in each of the study upazilas and comprised of community stakeholders – who were asked to provide input on the acceptability of implementing the PLA intervention after COVID-19 lockdown.’

Did the authors revised the PLA intervention based on their feedbacks? Please mention that.

We have previously published a paper describing the specific engagement with Community Advisory Groups in relation to delivery of our intervention in the context of COVID-19. This paper is cited in our manuscript. We have now also added a summary of key adaptations to the intervention that resulted from this community consultation.

In the individual questionnaire subheading, the authors stated that they used a mental health screening tool, but they did not mention their rationale for using it earlier in the manuscript. Was the tool validated from the perspective of Bangladesh? If not, how did they validate it in the context of Bangladesh? Please provide clarification.

We have added rationale for screening for depression and anxiety in our study population and provide additional references detailing application of the PHQ-9 and GAD-7 tools in Bangladesh.

In the physical measurement subheading, the authors mentioned Measurements of height, weight, and waist and hip girth were taken with light clothes without shoes and using standard methods. Need to cite a reference there.

Citation now added.

In the sample size subheading, the authors mentioned that (4) village size (smaller vs. larger villages). How did they categorize it? For example: 4 or fewer villages considered a small and more than that considered large villages?

We clarify in the revised manuscript that this relates to comparisons between villages with a population size less than the mean population size of all villages vs. those with a population size greater than or equal to the mean population size.

Result
From Line 377, page no. 9, the authors showed some data but it’s not clear which table represents the data as they described Table 4 from their previous sentence. The data shown is from Table 3. Please check it.

We have corrected the table number in the text.

On page 14, starting from Line 405, the authors discussed post-hoc analyses of blood pressure and presented the results. However, they did not refer to any supplementary table or provide one. Therefore, it is recommended that the authors add a supplementary file with the relevant blood pressure data.

We have now added a supplementary table which presents these data.

From Line 432, page no. 14, the authors provided breakdown of the intervention costs though the supplementary file 4 showed the sensitivity analysis data. From Line 432, page no. 14, the authors detailed the intervention costs, while the supplementary file 4 showed the sensitivity analysis data. Please check it. However, Supplementary Table 6 displayed intervention costs data. Please verify and make the necessary corrections.

We have checked and corrected all table numbers.

Discussion
The authors referred to Table 2 in the discussion section, but it is recommended not to include result tables in the discussion section.

We have removed reference to results in the Discussion section.

From Lines 477-481, on page 15, the authors are advised to break it into two sentences, which will make it easier to understand.

We have edited and split the sentence to improve clarity.

Conclusion
The authors are suggested to include recommnedation on how to make their project sustainable and how to integrate it into the health policy system.

Sustainability and integration into health systems and policy is certainly an important consideration, however we believe it is beyond the scope of this trial paper’s objectives and presented data to make recommendations here.

Minor comments
Abstract
Participants
The authors mentioned Adults aged >30 years who permanently reside in study villages, some places they mentioned ≥30 years. Please check this all over the manuscript and correct it.

We have checked the manuscript and corrected all to ≥30.

Intervention
The authors started with 108 PLA groups were created. The authors are suggested to splled it out. Example: One hundred and eight.

We have corrected this.

Method section of the main manuscript
The authors may include the study duration if the word count permits.

The study duration is reported at the start of the Methods section.

In the main manuscript, in the randomisation and masking subheading, the authors mentioned 12 folded pieces of paper. Please spell out 12 with Twelve.

We have corrected this.

In the main manuscript, in the intervention subheading, the authors mentioned 108 PLA groups, 9 men’s and 9 women’s in each intervention cluster, please spell out 108 with one hundred and eight.

We have corrected this.

In sattistical analysis subheading, please add the Stata version.

We have added this detail.

Reviewer #2: Many thanks for a clear, well-written article.

Where are the data able to be viewed?

We now provide details of the data repository where data can be viewed.

Line 512: please re-read this line as I think it has an extra “covered was $12”.

We have corrected this.

Reviewer #3:

The manuscript is written well. The background section is missing and should be included.

We have expanded the Introduction section to provide more global and national context for our study.

On the ethical concerns please indicate what happened to individuals who had higher blood glucose and blood pressure that needs treatment, at both clusters in baseline and endline.

We have added details to Methods section of the manuscript (under the *Physical Measurements* heading). Briefly, all participants were provided with the results from the physical measurements they participated in and were signposted to health services for further testing, advice and treatment.

If possible strengthen the discussion with other similar studies beside the DMagic study output.

Other than DMagic, we are not aware of and are unable to identify any other studies of population-level, community-based participatory interventions for diabetes prevention and control.

And explore what could other possible reasons in addition to the COVID-19 adaptations have an effect in the outcome measures. Support some of the results with qualitative findings if available at hand.

We report findings from our detailed mixed-methods process evaluation in another paper now published in BMJ Open and now cited in our revised manuscript. This paper describes key contextual factors and their potential impacts on intervention implementation and effectiveness.
