# Supplementary material for: A cluster randomised controlled trial of community groups using Participatory Learning and Action to prevent and control diabetes and intermediate hyperglycaemia in rural Bangladesh
Source: PLOS Glob Public Health. 2025 Aug 14;5(8):e0005049. doi: 10.1371/journal.pgph.0005049 (PMC12352636; doi:10.1371/journal.pgph.0005049)
Supplement: Attachment — Submitted filename: 20250721_ReviewResponse.docx [file pgph.0005049.s017.docx]

21^st^ July 2025

Dear Dr Robinson,

Thank you for your intervention with our paper and for the opportunity to respond to the latest round of reviews. We provide a point-by-point response to editorial comments and reviewer comments below. This has resulted in only very minor changes to our manuscript, which have uploaded as a clean and tracked-changes version.

We hope that our responses and the minor changes made will help you to reach a speedy decision on publishing our manuscript.

Yours sincerely,

Ed Fottrell

**Editorial Comments**

Please review your reference list to ensure that it is complete and correct. If you have cited papers that have been retracted, please include the rationale for doing so in the manuscript text, or remove these references and replace them with relevant current references. Any changes to the reference list should be mentioned in the rebuttal letter that accompanies your revised manuscript. If you need to cite a retracted article, indicate the article’s retracted status in the References list and also include a citation and full reference for the retraction notice.

We have not made any changes to the references.

Please provide separate main figure files in .tif or .eps format only and remove any figures embedded in your manuscript file. Please also ensure that all files are under our size limit of 10MB. Please leave the figure captions or legends in the manuscript.

For more information about how to convert your figure files please see our guidelines: <https://journals.plos.org/globalpublichealth/s/figures>

We now provide our figure as a .tif file and have left the figure caption in the manuscript.

We notice that your supplementary tables are included in the manuscript file. Please remove them and upload them with the file type 'Supporting Information'. Please ensure that each Supporting Information file has a legend listed in the manuscript before or before the references list.

We have provided all supplementary tables in a single file and uploaded this as ‘Supporting Information’. Each supplementary table has a legend listed in the manuscript after the references.

We have noticed that you have uploaded Supporting Information files, but you have not included a list of legends. Please add a full list of legends for your Supporting Information files before or after the references list.

Our revised manuscript includes a full list of headings for the supplementary tables, provided as supporting information files.

Please amend your online detailed Financial Disclosure statement. This is published with the article. It must therefore be completed in full sentences and contain the exact wording you wish to be published.

a) State the initials, alongside each funding source, of each author to receive each grant, if applicable. For example: "This work was supported by the National Institutes of Health (####### to AM; ###### to CJ) and the National Science Foundation (###### to AM)."

b) State what role the funders took in the study. If the funders had no role in your study, please state: “The funders had no role in study design, data collection and analysis, decision to publish, or preparation of the manuscript.”

The Financial Disclosure Statement is complete and accurate.

Please ensure that the funders and grant numbers match between the Financial Disclosure field and the Funding Information tab in your submission form. Note that the funders must be provided in the same order in both places as well.

The information is correct and the same in both places.

**Response to reviewer comments**

We are grateful to all reviewers for their appraisal of our work. Only Reviewer 5 had further comments. In places these are quite difficult to understand, but we have attempted to respond to each comment and provide details of any revisions made.

In response to question 5 (“Is the manuscript presented in an intelligible fashion and written in standard English?”) reviewer 5 responded “No” but does not provide clear description of the problem. The lead author of this paper, and many of the co-authors, are native English speakers and educated to advanced degrees in the UK. We believe our written English is of a high standard. Any further guidance on this from the Editor will be welcomed.

Summarize the title. suggestion of short title: (effect of participatory learning and action in regulation of blood glucose)
• using of abbreviation in title is not preferable and ambiguous.

The title of the study, including the abbreviation ‘DClare’ used in our short title, is linked to our trial registration and related communications and publications. Our current title is precise and informative. We have spelled out PLA in the short title for clarity but otherwise do not feel it is necessary to change our title.

Covid 19- is one of the two important study objectives?

It is unclear what the reviewer means here. COVID-19 in our abstract relates to study context, not to any specific study objective. We have edited the abstract slightly by removing reference to COVID in our Objectives section, but still describing the need to adapt intervention delivery due to COVID in the Methods section of the Abstract.

Key word must be novel or infrequent.
Suggestion: diabetes, community intervention

Keywords were developed with thought and by consulting common MESH terms to enable readers to find our article. We have provided just six key terms and do not believe that they need to be revised.

With regards to our Methods section, Reviewer 5 makes a number of suggestions detailed below:

1. write in specific titles with brief and meaning ful [sic] manner.

2. rearrange methods as to give complete idea in each paragraph regarding Study design and area, Study subjects, Inclusion criteria, Exclusion criteria, methods for data Collection and Preparation of Samples and grouping of study participants.

3. In each sub title author must write all date regarding the specific paragraph for one time to avoid repetition of information and to divide the research paper in well known title(abstract, introduction, methods….etc)

We do not feel these edits are necessary nor would they improve our manuscript. Our presentation and sub-headings meet convention for a detailed methods description, aligned with CONSROT reporting standards for cluster randomised controlled trials.

4. set your participants in methodology as groups i.e.; diabetic group, , control group, intervention group, other non communicable disease group

5. write how the author group the study participant.

6. ignore any participant who were not in the inclusion criteria. Write the exact number of study participants only.

Our study is a cluster randomised controlled trial and we have reported it using CONSORT standards, including clear description or randomisation and allocation of intervention and control groups. We do not believe any further edits are needed here.

7. Give explanation for covid 19 include its impact in study participant

It is not entirely clear what the reviewer is requesting here. We have written in detail about the effect of COVID-19 on our study. We are clear in our abstract and Introduction that our study was delivered in the context of the evolving COVID-19 pandemic. We have published separately on how COVID-19 may have impacted our intervention and cite these papers in our manuscript (citations 13 and 22).

8. If the covid 19 effect the sample size

Our methods section details that due to COVID-19 adaptations to our study design (moving from a stepped-wedge cluster randomised controlled trial to a parallel arm trial), we updated our sample size calculation. We have no way of knowing whether survey response rates were impacted by COVID-19, but have no reason to believe they were. Our Results describe survey response rates, which were excellent.

9. mention the noncommunicable disease included in current study.

These are detailed in our Methods section, supplementary table and in Results table in our submitted manuscript.

Results:
• write every result of specific table above the table then jump to the next table.

It is unclear what is being requested here. We feel that our presentation of results is complete, efficient and in line with standard reporting practice.

Discussion:
Good, but there is elaboration in this section.

The meaning here is unclear, but we will take it as a compliment that our discussion successfully elaborates on, contextualises and seeks to interpret our results.
